# Acknowledgment to the Reviewers of *Nanomaterials* in 2022

**DOI:** 10.3390/nano13020356

**Published:** 2023-01-16

**Authors:** Nanomaterials Editorial Office

**Affiliations:** MDPI AG, St. Alban-Anlage 66, 4052 Basel, Switzerland

High-quality academic publishing is built on rigorous peer review. *Nanomaterials* was able to uphold its high standards for published papers due to the outstanding efforts of our reviewers. Thanks to the efforts of our reviewers in 2022, the median time to first decision was 12.7 days and the median time to publication was 2.9 days. Regardless of whether the articles they examined were ultimately published, the editors would like to express their appreciation and thank the following reviewers for the time and dedication that they have shown *Nanomaterials*: Aabid, AbdulLu, MingyangAamir, MuhammadLu, QilinAbabei, GabrielLu, QiongqiongAbad, BegoñaLu, ShanAbadel, ArefLu, ShixiangAbbas, HaiderLu, ShulongAbbas, NaseemLu, SiyuAbbas, Syed MuzahirLu, TianAbbasi Moud, ArefLu, WanhengAbbrent, SabinaLu, WeibangAbd El-Mageed, Ahmed I.A.Lu, XiaAbdel-Aty, Abdel-HaleemLu, XiaoquanAbdel-Aty, MahmoudLu, YangchengAbdElgawad, HamadaLu, YanghuaAbdel-Hady, Esam El-SayedLu, YuexiangAbd-Elhameed, Waleed MLü, YupengAbdelhamid, HaniLu, ZhilunAbdeljawad, Fadi FLuan, BinquanAbdelkarim, AydiLubczak, JacekAbdelsalam, Sara I.Lucaciu, Constantin MihaiAbderrahmane, AbdelkaderLucchetta, Daniele EugenioAbdollahifar, MozaffarLuchian, IonutAbdollahramezani, SajjadLuchian, TudorAbdollahzadeh Jamalabadi, Mohammad YaghoubLuciani, GiuseppinaAbdolrazzaghi, MohammadLuciano, GiorgioAbdul, Wahab MohammadLuck, RudyAbdulaziz, BatiLugo, Jesus EduardoAbdulhalim, IbrahimLukaszewski, ZenonAbdul-Hamid, Muhammad AzmiLukoyanov, AlexeyAbdulwahid, RebarLuk’yanchuk, IgorAbe, KentaroLukyanov, Pavel A.Abedi, Hamid RezaLuliński, PiotrAbejón, RicardoLuminita, IonescuAbenojar, EricLuna, CarlosAbouelmagd, Elbaz I.Luo, BinbinAbou-Neel, Ensanya AliLuo, DaibingAbramkin, DemidLuo, DanAbramov, PavelLuo, DeyingAbrosimova, G.Luo, FubinAbu-Reziq, RaedLuo, HaoAccorsi, GianlucaLuo, JianAcharya, NilankushLuo, JianbinAcken, John M.Luo, JingAdachi, MichaelLuo, KuiAdachi, SadaoLuo, LiqiangAdamiano, AlessioLuo, WenAdams, AlinaLuo, XiangangAdamski, AndrzejLuo, ZhuAdányi, NóraLuongo, GiuseppeAdawy, AlaaLuther, Joseph M.Adhikari, RajdeepLutsyk, PetroAdil, Syed FarooqLuty-Błocho, MagdalenaAdnan, MdLuxbacher, ThomasAdnan, MuhammadLuzi, FrancescaAdriana, CambónLv, HongjinAflori, MagdalenaLv, LinAfzalian, AryanLv, SongweiAga, OmerLv, ZhibinAgarwala, ShwetaLv, ZiyuAgha, ImadLy, Thuc HueAgrawal, AshishLyashenko, IakovAguila, BrianaLyer, StefanAgustin, DominiqueLyons, John L.Ahamed, MaqusoodLysova, Anna A.Ahammad, N.AmeerLyu, HonghongAhmad, AjazLyu, YezheAhmad, AkilLyu, ZhaoyuanAhmad, AnasM Gómez, ClaraAhmad, AwaisM. Mostafa, AymanAhmad, IjazMa, Chih-MingAhmad, JavedMa, Fa-JunAhmad, KhursheedMa, GangAhmad, MashkoorMa, GuangyiAhmad, Mohd ZamidiMa, JieAhmad, RafiqMa, LonglongAhmadi, NimaMa, LongtaoAhmadivand, ArashMa, RuijieAhmed, Atallah F.Ma, ShaojieAhmed, BilalMa, WanhongAhmed, Hawreen HasanMa, WenjingAhmed, MaryaMa, XiaolingAhmed, MohammadMa, XiaoqianAhmedova, AnifeMa, YufeiAhn, Chang WonMa, Yunn-HwaAhn, DoyeolMa, ZengshengAhn, Jae-HyukMa, ZhenAhn, NamyoungMa, ZhuoranAhn, SeokhoonMa, ZichuanAhn, Sung HoonMaas, MichaelAi, BinMaasilta, IlariAikawa, ShinyaMacagnano, AntonellaAissat, AbdelkaderMacak, JanAivaras Kareiva, HabilMaccaferri, NicolòAjmal, MuhammadMaccato, ChiaraAjori, ShahramMace, CharlesAkbari, AliMachado, RicardoAkbaş, Şeref DoǧuşcanMaciej, ThomasAkhavan, OmidMackinnon, Ian D.R.Akhtaruzzaman, Md.Macoas, ErmelindaAkhter, FirozMacucci, MassimoAkimoto, IkukoMacutkevic, JanAkimov, AlexeyMączka, WandaAkitsu, TakashiroMadejczyk, PawelAkyurtlu, AlkimMadhyastha, HarishkumarAl Zoubi, WailMadrid, RossanaAl-Ahmad, AliMadsen, Steen J.Alak, GoncaMaduraiveeran, GovindhanAlam, ManawwerMaeda, HiroakiAlam, MehebubMaeda, YutakaAlarco, JoseMaenosono, ShinyaAlbano, GianluigiMaesato, MitsuhikoAlbano, MarcoMaezawa, KoichiAlberta, LuccheseMagalhães, SérgioAlbo, JonathanMagalhães, Washington Luiz EstevesAlbonetti, CristianoMagasinski, Alexandre N.Albulescu, RaduMagdalena, SałdykaAl-Buriahi, M.S.Magdysyuk, Oxana V.Alcántara-Ávila, Jesús RafaelMagkoev, Tamerlan T.Alcaraz, Jean-PierreMaglio, MelaniaAlcudia, AnaMagna, GabrieleAlegre, EstíbalizMagnani, AgneseAlegret, NuriaMagomedov, ArtiomAleksandrov, AlexeyMagoulas, GeorgeAlekseeva, Anastasia M.Mahanthesh, B.Aleshin, AndreyMahapatra, ChinmayaAleshkin, VladimirMahapatra, Manoj K.Alessandri, IvanoMahata, ChandreswarAlexiev, UlrikeMahato, NeelimaAlexiou, ChristophMahdi, Jasim M.Alexis, JoelMahmood, Mastani JoybariAlexovič, MichalMahmoodi, NiyazAlfano, BrigidaMahmoud, Amouzadeh TabriziAlfè, MichelaMahmoudi, FarzanehAlfei, SilvanaMahmud, SakilAl-Gheethi, AdelMahy, Julien G.Algorri, José FranciscoMaier, Stelian SergiuAlhammadi, SalhMaile, NageshAli, BaghMaimistov, Andrei I.Ali, Gomaa A.M.Mair, Lamar O.Ali, Hayssam M.Maireles Torres, Pedro JesusAli, ImranMaiti, BinoyAli, KhatibiMaiti, SaikatAli, LiaqatMaiti, SandipanAli, Mohamed El-SayedMajak, JüriAli, Mohamed Mamdouh M.Majdan, MarekAli, SajadMajeed, AbdulAlim, Hakan OrmeciMakarov, DmitryAliofkhazraei, MahmoodMakarov, SergeyAlique Amor, DavidMakarov, Vladimir IgorevichAllen, Norman S.Makaryan, TaronAllizond, ValeriaMakky, AliAlloin, FannieMaksimchuk, NataliyaAlloncle, Anne Patricia B.Maksimov, Dmitrii N.AlMangour, BandarMalandrino, GraziellaAlmáši, MiroslavMalandrino, MeryAlmásy, LászlóMalankowska, AnnaAl-Mdallal, Qasem M.Malara, PietroAlmeida, Bernardo GonçalvesMalatesti, NelaAlmeida, ManuelMaldonado, José-LuisAlmeida, RuiMale, David K.Almpanis, E.Malecka, KamilaAlmquist, CatherineMálek, PřemyslAloi, DanielMalekshah, Emad HasaniAloisio, AlbertoMalik, MaqsoodAlonso, FranciscoMalikan, MohammadAlonso-Vante, NicolasMalinowski, RafałAlotaibi, Mohammad HayalMaliszewska, Irena H.Alpuche-Aviles, Mario A.Malka, DrorAlqadami, AyoubMałkowski, EugeniuszAlshehri, Saad M.Malliakas, ChristosÁlvarez Centeno, TeresaMałolepszy, ArturÁlvarez-Alonso, PabloMalucelli, GiulioAlves Galico, DiogoMamat, Mohamad HafizAlves, HelenaMamidi, NarsimhaAlves, LuisMamiński, MariuszAlves, PatríciaMamiyev, ZaminAlwarthan, AbdulrahmanManaila-Maximean, DoinaAly, Arafa HManakhov, AntonAmaterz, ElhassanManavalan, ShaktivelAmeen, FuadManca, Maria LetiziaAmekura, HiroshiMancheño Real, María JoséAmendola, VincenzoMancini, MarcoAmghouz, ZakariaeMancuso, RaffaellaAmin, HardikManda, RameshAmin, MuhammadMandal, ArkajitAmine, AzizMandal, GopinathAmirov, AbdulkarimMandal, SumitAmit, KumarMandal, TapasAmjad, Muhammad WahabMandelli, DavideAn, CuihuaMandić, VilkoAn, QinyouMandracci, PietroAn, SangminMandzhieva, Saglara S.An, XingyeMane, SagarAna Belén, Soldado CabezueloManea, FloricaAnanth, AntonyManeiro, MarcelinoAnanthakrishnan, Soundaram JeevarathinamManera, Maria GraziaAnanyev, Ivan V.Mani, GovindasamyAnanyev, MaximManini, PaolaAnashkina, ElenaManjunatha Reddy, G.N.Anastassiu, Hristos T.Mann, Rajinder S.Anca, Roseanu ConstantinescuManna, SantanuAnđelić, NikolaManokar, A.MuthuAndo, TakashiMansfield, ElisabethAndo, YoshitoMansourpanah, YaghoubAndraca, Jose AlbertoMantione, DanieleAndrade, LuísaManuel Ortiz, JuanAndreani, TatianaManuel, Algarra GonzálezAndreev, Vladimir V.Manukovskaya, Diana V.Andreeva, MarinaMao, YanchaoAndreoli, EnricoMarabelli, FrancoAndreou, ChrysafisMaravić, TatjanaAndres, JuanMarc, PawelAndrianov, AlexeyMarcano, AristidesAndronic, LuminitaMarchuk, OleksandrAndroš Dubraja, LidijaMarco, Vittori AntisariAndroulidaki, MariaMarconcini, PaoloAndrulevicius, MindaugasMarcos, Paula M.Andrzejak, MarcinMarcu, AurelianAngelini, EmmaMarcu, Ioan CezarAngelo, AngeliniMarcus, Matthew A.Angelopoulou, AthinaMardare, Andrei IonutAngelov, BorislavMareev, E.I.Anggraini, Sri AyuMarek, TkaczAnghel, Elena MariaMargueron, SamuelAnia-Castanon, Juan DiegoMarí, MontserratAnicai, LianaMaria Joseph Raj, Nirmal PrashanthAnimansaun, Isaac LareMaria, Vera LúciaAnioł, MirosławMariani, PaoloAnis, BadawiMariani, StefanoAnitas, EugenMariappan, Vimal KumarAnitas, Eugen MirceaMarić, MilanAnnese, Valerio FrancescoMarichy, CatherineAnni, MarcoMariello, MassimoAnoop, GopinathanMarín, Josefa IsasiAnpo, MasakazuMarin, LuminitaAnsari, D.Mohd.AzamMarin, Nicoleta MirelaAnsari, Sajid AliMarin, PilarAnson, ChristopherMarin, Ricardo MallaviaAntal, IstvánMarinkovic, DraganAntohe, ŞtefanMarinkovic, NebojsaAntonietta, ParracinoMarinov, Daniil L.Antonino, FamulariMarinova, VeraAntonio, CarellaMariotti, DavideAntonio, Riveiro RodriguezMariya, SedelnikovaAntonova, Irina V.Marken, FrankAnttu, NicklasMarkoli, BoštjanAnwar, SabiehMarković, ZoranAnwar, ZahidMarkowicz-Piasecka, MagdalenaAnwar-ul-Haq, Ali ShahMarkus, FerencAoyagi, ShinobuMarlinda, A.R.Apicella, AntonioMarmiroli, BenedettaApostolova, Margarita D.Marotti De Sciarra, FrancescoApostoluk, Aleksandra (Alexandra)Marousek, JosefAquilano, DinoMarović, DanijelaAra, IreneMarques, CarlosArakawa, TakahiroMarques, LuisArampatzis, IoannisMarques, Manuel Joaquim BastosAranda, DanielMárquez, FranciscoAraya-Hermosilla, Esteban AlejandroMarquina, ClaraArbiol, JordiMarra, FabrizioArce, PedroMarradi, MarcoArdelean, Lavinia CosminaMarrazzo, Vincenzo RomanoArena, MaurizioMarrero-López, DavidArévalo, M.CarmenMarri, Anil ReddyArguilla, MaxxMars, KrzysztofArgyle, MorrisMarsili, EnricoArief, InjamamulMarszałek, MartaAriga, KatsuhikoMarszewski, MichalArita, MasanoriMartella, DanieleArkas, MichaelMartí-Centelles, VicenteArmaghani, TaherMartín Benenzuela, Inocencio RafaelArmentano, IlariaMartín, CristinaArmetta, FrancescoMartín, Eduardo L.Arnaiz, BlancaMartin, Francis LArnould, MarkMartin, Inocencio R.Arsenin, Aleksey V.Martin, PascalArtal, EduardoMartin, PatrickArteaga-Pérez, Luis E.Martinelli, MichelaArtem’ev, Alexander V.Martines, EmilioArthur, YelonMartinez Pedrero, FernandoAruchamy, KanakarajMartínez Rodrigo, JavierArun Kumar, Narayanan NairMartínez, José IgnacioArunachalam, PrabhakarnMartinez, LidiaArutyunyan, N.R.Martinez, VirginiaAsadi, AminMartínez-Campos, EnriqueAsai, DaisukeMartínez-Lillo, JoséAsandei, AlinaMartínez-Pastor, JuanAsaro, FiorettaMartinez-Triguero, JoaquinAsgari, SomayyehMartin-Garcia, BeatrizAsghar, Muhammad AdnanMartín-Gil, JesusAshida, MasaakiMartini, ElisabettaAshkarran, AliakbarMartino, MarcoAshour, HossamMartins Amaro, Ana Paula BetencourtAshrafi, AmirmansoorMartins, Jorge N.R.Ashuri, MaziarMartins, José A.Aslfattahi, NavidMartins, Luis R.M.Ašmontas, SteponasMartins, Mónia A.R.Asokan, ArunchanderMartins, RodrigoAssadi, HusseinMartins, Rui C.Assael, Marc J.Martucciello, NadiaAssimakopoulos, VasilikiMartynenko, IrinaAssis, MarceloMartyushev, Nikita V.Assmann, MarcMaruccio, GiuseppeAstolfi, PaolaMarullo, SalvatoreAsubar, Joel T.Maruschak, PavloAta, SeisukeMaruyama, ShingoAtabaev, Timur Sh.Masaaki, AbeAtanase, LeonardMascher, PeterAtanase, Leonard-IonutMaserati, LorenzoAtanasov, VladimirMashaan, NuhaAtaullakhanov, FazoilMasi, SofiaAtchudan, RajiMaslov, MikhailAtienzar, PedroMaslov, Mikhail MAtkinson, IrinaMasoodi, AmirrezaAtobe, MasakazuMasoumilari, ZohrehsadatAtrei, AndreaMasrour, RachidAttanasio, CarmineMassaro, MarinaAttia, Nour F.Mastelaro, Valmor R.Atuchin, VictorMastria, RosannaAtula S.D., SandanayakaMasud, JahangirAusanio, GiovanniMasui, ToshiyukiAutieri, CarmineMatarese, GiovanniAvadanei, MihaelaMatczuk, MagdalenaAvakyan, LeonMatea, Cristian TudorAvallone, BiceMatei, CristianAverin, Alexei DmitrievichMatejec, VlastimilAvetissov, IgorMaterer, Nicholas F.Avgouropoulos, GeorgeMather, GlennÁvila, JorgeMathwig, KlausAvilov, VadimMatijošius, JonasAvram, MarioaraMatko, VojkoAvram, Marius AndreiMatras-Postołek, KatarzynaAvramopoulos, AggelosMatsoukas, ThemisAvramova, IvalinaMatsuda, IwaoAvrutin, Eugene A.Matsuda, JunkoAvrutina, OlgaMatsuda, ShoichiAvti, PramodMatsumura, SyoAw, Kean C.Matsutani, AkihiroAwad, Mohamed M.Mattea, FacundoAwni, RashaMatteis, Fabio DeAwual, Md RabiulMatvienko, DmitryAxet, M.RosaMátyás, HunyadiAyaso, Francisco-JavierMatykiewicz, DanutaAyub, Prof. KhurshidMauclair, CyrilAzam, MuhammadMaulini, RichardAzlan, M.N.Maurya, KamleshAzuma, TakashiMautner, FranzBaba, KoumeiMayakrishnan, GopiramanBabaev, A.A.Maybeck, VanessaBabelova, AndreaMaye, Mathew M.Babeva, TsvetankaMayer, Alexander E.Babilas, RafałMayer, ThomasBaby, André RolimMazalski, PiotrBaccarani, GiorgioMazare, AncaBadea, NicoletaMažeika, DaliusBadri, Seyed HadiMazeika, KestutisBae, DowonMazierski, PawełBae, Jae YoungMazilkin, AndreyBae, JinboMazur, LeszekBae, Jin-HyukMazurkiewicz-Pawlicka, MartaBae, JoonhoMcGlynn, EndaBae, SeunghwanMcIlroy, David N.Baena, Francisco ManuelMcIntosh, RuaraidhBaev, AlexanderMcIntyre, Dustin L.Bagattini, MariaMcKay, Tina B.Bagdziunas, GintautasMcKendry, Jonathan James DonaldBahadorikhalili, SaeedMcLean, RobertBahadur, AliMcMurray, MichaelBahiraei, MehdiMcQuaid, Raymond G.P.Bahlawane, NaoufalMedaglia, Pier GianniBai, GongxunMedić, Mina M.Bai, JingMedvedev, DmitryBai, LichenMeena, Sher SinghBai, QingguoMeeyoo, VissanuBai, ShiMegiel, ElżbietaBai, ShuoMehra, Neelesh KumarBai, YuchaoMehryan, S.A.M.Bai, ZhenyuMehta, AkanshaBaia, MonicaMei, BastianBaibarac, MihaelaMei, ZengxiaBaidakova, Marina VMeier, CedrikBaimova, Julia A.Meier, Robert J.Baindara, PiyushMelchionna, MicheleBaïri, AbderrahmaneMeleppat, Ratheesh KumarBakardjieva, SnejanaMelguizo-Guijarro, ManuelBakhtiyarov, SayavurMélinon, PatriceBakina, Olga V.Melinte, VioletaBaklanov, Mikhail R.Mellau, GeorgBakos, IstvanMelnikov, PavelBakr Mohamed, MohamedMelnikov, StanislavBaksi, AnanyaMelnyk, InnaBakulin, AlexanderMeloni, EugenioBakurskiy, SergeyMenaa, FaridBalaev, Dmitrii A.Ménard-Moyon, CéciliaBalandin, AlexanderMenazea, AbdelrhmanBalandin, Alexander A.Mendes, Adriano A.Balaș, MihaelaMendez, Ana MariaBalasingam, Suresh KannanMéndez, E.R.Balasoiu, MariaMendoza, CarolinaBalayeva, Narmina O.Meneghini, CarloBalch, AlanMenendez, BeatrizBaldelli, AlbertoMeng, ChaoBaldino, LuciaMeng, DongBaldomir, DanielMeng, FanliBaldovi, JoseMeng, GuowenBalen, BiljanaMeng, QingbangBalijapalli, UmamaheshMeng, ShujuanBallarin, BarbaraMeng, YonggangBallinger, JimMengtao, SunBalogh, György T.Menichetti, LucaBalzer, FrankMensi, MounirBalzer, Jan C.Menyhárd, Dóra K.Ban, TakahikoMera, GabrielaBanach, MarcinMerabia, SamyBanciu, Marian GabrielMerano, MicheleBandara, Y.M. Nuwan D.Y.Meraviglia, SerenaBandyopadhyay, SupriyoMercante, LuizaBanerjee, WritamMercier, DavidBanerji, SourangsuMere, ArvoBanks, Craig E.Mereshchenko, AndreyBannov, AlexanderMergler, StefanBano, ShaziaMerkel, CoryBansmann, JoachimMerkle, RotrautBanstola, AsmitaMerkulov, Daniela ŠojićBao, JimingMerli, MarcelloBao, WeizhaiMertens, StijnBarabas, RekaMerwid-Ląd, AnnaBarai, Hasi RaniMesa, CamiloBaran, WojciechMesaros, AmaliaBaranauskiene, LinaMescola, AndreaBaranchikov, AlexanderMessina, AntoninoBaranek, PhilippeMessori, LuigiBarani, MahmoodMestre, Ana S.Baranović, GoranMeyer, Jerry R.Baranovskii, Evgenii S.Meyyappan, M.Baranovskii, SergeiMeziani, MohammedBaranowska-Korczyc, AanaMezzi, AlessioBaranowski, Jacek M.Mi, ChangwenBarbara, Šetina BatičMi, LanBarbasz, JakubMiakonkikh, Andrey V.Barberis, AntonioMiao, GuoxingBarbiellini, BernardoMiao, QingqingBarbinta-Patrascu, M. E.Micciulla, FedericoBarbinta-Patrascu, Marcela-ElisabetaMichalska, MonikaBarbu-Tudoran, LucianMichalska-Domańska, MartaBarea, Eva M.Michna, AnetaBarek, JiriMiclǎu, MarinelaBarek, JiříMiclaus, SimonaBarille, RegisMiculescu, MarianBarker, Philip JMicutz, MarinBaron, Camilo FlorianMidya, RivuBarreca, DavideMie, YasuhiroBarretta, RaffaeleMiecznikowski, KrzysztofBarry, CarolMiglietta, Maria LuciaBârsan, NarcisMigliore, AgostinoBarszczewska-Rybarek, IzabelaMihailescu, CarmenBartczak, PrzemyslawMihalik, MarianBarth, SvenMikhaylov, AlexeyBartha, CristinaMikheenko, PavloBartha, Maria CristinaMikheev, IvanBartoli, MattiaMikolajczak, RenataBartolomeo, Antonio DiMikšová, RomanaBartosz, GrzegorzMikulics, MartinBaruffaldi, FilippoMikuriya, MasahiroBasak, Animesh KumarMilanesio, MarcoBasavegowda, NagarajMilani, PaoloBaselga Llidó, JuanMilčius, DariusBasheeruddin, Syed MohammedMilcovich, GesmiBaskoutas, SotiriosMilekhin, AlexanderBasletić, MarioMileo, PauloBastidas, Jose-MariaMiletín, MiroslavBastola, EbinMileva, ElenaBastos Arrieta, JulioMileva, MilkaBastrzyk, AnnaMilia, EgleBasu, ShibomMilić, MirtaBasuray, SagnikMiller, Catherine M.Batasheva, SvetlanaMills, DavidBathula, Chinna DMiloshev, GeorgeBatish, Daizy R.Milowska, KarolinaBatoo, Khalid MujasamMilton, Kimball A.Bator, GrażynaMiluski, PiotrBattabyal, ManjushaMilyaev, MihkailBattiato, AlfioMin, JuBattiato, Sergio OrazioMin, RuiBattocchio, ChiaraMinaev, BorisBaumann, WolfgangMinakov, Alexander A.Baumgarten, MartinMinakshi, ManickamBäumler, HansMinakshi, MannyBaun, AndersMinami, IchiroBausells, JoanMinami, KosukeBautista, OscarMinardo, AldoBayat, MaryamMinea, AlinaBazaga-García, MontseMinea, Alina AdrianaBazzan, MarcoMineo, PlacidoBazzarelli, FabioMing, WuYiBeatriz, BarrocasMing, YangBecucci, MaurizioMinigalieva, Ilzira A.Bednarikova, ZuzanaMinin, Igor V.Beedasy, VimanyuMinisy, IslamBegaud, XavierMintz, Keenan J.Beghetto, ValentinaMio, Antonio MassimilianoBégin, DominiqueMiolo, GiorgiaBegoña, AbadMiotello, AntonioBehnamian, YasharMirabella, SalvoBeke, DezsőMiranda, AdelaideBeklemishev, Mikhail K.Miranda-Castro, RebecaBelashov, AndreyMiranowicz, AdamBelavič, DarkoMiritello, MariaBelcarz, AnnaMirkhani, Seyyed AlirezaBelenov, SergeyMirsky, VladimirBelik, Alexei A.Mirsky, Vladimir M.Belim, SergeyMirzaei, AliBeljebbar, AbdelilahMirzaei, MasoudBell, Nelson S.Mishakov, IlyaBella, FedericoMishra, AdityaBellardita, MariannaMishra, AwdheshBellucci, AlessandroMishra, RajeshBellucci, StefanoMishra, Ranjeet KumarBelousov, YuryMishra, Satyendra KumarBelskaya, Olga B.Misko, Vyacheslav R.Beltrami, RiccardoMisoguti, LinoBeltran, Juan IgnacioMissell, Frank P.Belyakov, SergeyMissyul, AlexanderBender, DirkMitchell, Brian S.Bending, SimonMitchell, GeoffreyBenea, DianaMitchell, John S.Benedetti, AlessioMititelu Tartau, LilianaBenedetti, StefaniaMitomo, HideyukiBenedikovic, DanielMitoraj, MariuszBenesperi, IacopoMitraki, AnnaBenis, Emmanouil P.Mitran, GheorghitaBenitez De La Torre, AlmudenaMitrofanov, AnatolyBennett, Steven PMitropoulos, Athanasios C.Benoît, JouaultMittova, Irina YaBenzarti, ZohraMitus, Antoni C.Berciu, MonaMityushev, VladimirBerendzen, Kenneth W.Miyazaki, Hideki T.Bergamaschini, RobertoMiyazawa, TaikiBergmann, RalfMiyoshi, HiroakiBerillo, DmitriyMizoshiri, MizueBerkó, AndrásMizoshita, NorihiroBermudez, María DoloresMizsei, JanosBernard, GeffroyMjakin, SergeyBernard, HumbertMladenov, ValeriBernardeschi, MargheritaMo, FunianBernardi, EttoreMo, SongpingBernascon, LeonardoMo, ZunliBernasconi, RobertoMobedi, MoghtadaBernatová, SilvieMoccia, MassimoBerndt, Christopher C.Mochán, W. LuisBernik, SlavkoModec, BarbaraBernstein, ElliotMoehrle, MartinBernstein, Elliot R.Moghanlou, Farhad SadeghBerrie, CindyMohaček-Grošev, VlastaBerríos, CristhianMohajernia, ShivaBert, NikolayMohamad Ibrahim, Mohamad NasirBerthebaud, DavidMohamad, Ahmad AzminBerto, MarcelloMohamed, Ahmed A.Bertoncello, PaoloMohamed, El-SaadonyBertram, FrankMohamed, IbrahimBertsch, PaulMohamed, Mohamed GamalBesenhard, MaximilianMohammadehtisham, KhanBesford, Quinn A.Mohanta, AntaryamiBeşleaga, CristinaMohapatra, JeotikantaBesnard, AurélienMohapatra, S. C.Bespalko, Yulia N.Mohd Salleh, Mohd Arif AnuarBessa, LucindaMohd-Yasin, FaisalBessais, LotfiMoiseev, KonstantinBessmertnykh-Lemeune, AllaMoiseev, NikitaBetke, UlfMoiseev, SergeyBettini, SimonaMokrushin, ArtemBettmer, JorgMolak, AndrzejBeugeling, WouterMolčan, MatúšBhadra, Biswa NathMolchanov, VyacheslavBhalla, NikhilMolinari, AlessandraBhandari, KhagendraMolíns-Legua, CarmenBhanvase, Bharat A.Möller, KarinBhat, Mahesh P.Molnar, ArpadBhatia, DivijMomoniat, EbrahimBhattacharjee, SouravMonai, MatteoBhatti, M.MMondal, AniruddhaBhuyan, Manoj KumarMondal, KunalBi, HongMondal, Pranab KumarBi, JingtaoMondal, SubhadipBi, MingshuMonfort, OlivierBi, SaiMontagne, AlexBi, ShengMontañes, Rosa RodriguezBian, FenggangMontaño, Gabriel A.Bian, WenjuanMonteiro Baptista, António PauloBian, ZhoufengMontemurro, DomenicoBianchi, MassimilianoMontenegro, LuciaBiczak, RobertMontes, VicenteBielas, RafałMontoncello, FedericoBienkowski, KrzysztofMontoya, Oscar DaniloBiernat, JanMoon, Cheon WooBiesuz, MattiaMoon, JanghyukBietti, SergioMoon, Kyoung IlBiffis, AndreaMoore, Matthew D.Bihlmayer, GustavMooste, MarekBikiaris, DimitriosMoraczewski, KrzysztofBilad, Muhammad RoilMorales, AlfredoBilal, MuhammadMorant, CarmenBilal, SalmaMorasso, CarloBiliuta, GabrielaMorávková, ZuzanaBillah, Mohammad MasumMoreau, JulienBilusic, AnteMoreira, André FerreiraBini, MarcellaMoreira, Joaquim AgostinhoBirke, Ronald L.Morel, AdrienBírošová, LuciaMoreno, CésarBischof, HelmutMoreno, Karla J.Bischof, SandraMoreno-Tost, RamónBisello, DarioMoreva, EkaterinaBisio, FrancescoMorgiel, JerzyBisson, Jean-FrançoisMori, WasukeBiswas, ChandanMorikawa, OsamuBittencourt, CarlaMorimoto, MasakazuBittner, AlexanderMorimoto, YasuoBizzarri, Anna RitaMoritake, YutoBjorklund, RobertMoriya, MakotoBlach, Jean-FrançoisMoros, MaríaBłachowicz, TomaszMorozov, Sergey V.Blackman, ChrisMorozov, Vladimir N.Blaikie, RichardMorozyuk, TetyanaBlake, GraemeMortazavi, BohayraBlanquer, Sébastien B.G.Mortimer, MonikaBlase, XavierMosavi, Amir H.Blaszczuk, ArturMoshnyaga, VasilyBlau, WernerMosiałek, Michał AndrzejBlaudeck, ThomasMoskalik, Mikhail YuBlázquez, ManuelMosquera, MartaBleotu, CoraliaMossi, KarlaBloch, WitoldMostafavi, MehranBlonkowski, SergeMostovoy, AntonBocchetta, PatriziaMotogaito, AtsushiBoccuni, FabioMotoyama, MunekazuBocharov, DmitryMott, Derrick M.Bodaghi, MahdiMotyka, MaciejBodzenta, JerzyMouchet, SébastienBoeglin, AlexMouchliadis, LeonidasBoffito, MonicaMoulaee, KavehBogdanov, Kirill V.Moulick, AmitavaBoguta, PatrycjaMoura Aouada, Marcia Regina DeBohinc, KlemenMourdikoudis, StefanosBohman, BjörnMourzenko, ValeriBoi, Filippo S.Mourzina, YuliaBojarski, PiotrMousa, Hamouda M.Bojinov, MartinMousavi, Mohsen BeladiBokach, NadezhdaMousavi, Seyyed MojtabaBokhari, AwaisMousdis, George A.Bokias, GeorgiosMoustafa, HeshamBollani, MonicaMovahedi, NimaBolotov, LeonidMoya, Antonio A.Bonadies, IreneMu, LiBonardd, SebastianMu, XiaojingBonasera, AurelioMu’azu, Nuhu DalhatBoncel, SlawomirMucalo, Michael R.Bončina, TonicaMuhammad Ali, HafizBondarev, Andrey V.Muhammad Noor, Ahmad ShukriBoni, MihaiMuhammad, Mazhar IqbalBonomo, MatteoMuhammad, RazBonvicini, FrancescaMühlberger, MichaelBonyár, AttilaMuhmood, TahirBoqi, XiaoMukai, MasaruBorderon, CarolineMukherjee, SoumyaBordet, AlexisMula, GuidoBordo, VladimirMulders, J.J.L.Borek, WojciechMuller, GerhardBorelli, Deva Prasad RajuMüller, Thomas ErnstBoreman, Glenn D.Mullins, Oliver C.Borgekov, DarynMuneshwar, TriratnaBorges, JoelMunir, KhurramBorghesani, Armando FrancescoMunnik, FransBorghi, FrancescaMuñoz, EnriqueBorhan, Adrian IulianMuñoz-Bonilla, AlexandraBormashenko, EdwardMuñoz-Santiuste, Juan E.Boroica, LucicaMuntean, SimonaBorri, SimoneMunteanu, Florentina-DanielaBorriello, AnnaMurab, SumitBorse, PramodMurali, ArunBorská, LenkaMurali, G.Bosacka, MonikaMurali, GunasekaranBose, Rajendran JCMuralt, PaulBosnar, SanjaMuramatsu, AtsushiBossu, MaurizioMuramatsu, HiroyukiBotta, ChiaraMuravsky, AlexanderBotti, SabinaMurcia Mesa, Julie JoseaneBoubeta, CarlosMurcia, PedroBouchemal, KawtharMureseanu, MihaelaBoudart, BertrandMurias, MarekBouhala, LyazidMurshed, SmsBoulet, PascalMurthy, Tammana S.R.C.Bourillot, EricMusiał, AnnaBoury, BrunoMusmarra, DinoBousfield, Douglas W.Mussi, ValentinaBowen, ChrisMustafina, AsiyaBoybay, Muhammed SaidMusto, MarilenaBoyles, Matthew S.P.Mustoe, George E.Boytsova, OlgaMusuc, Adina MagdalenaBraconi, DanielaMuthupandian, SaravananBraga, HelenaMuzykantov, Vladimir R.Braga, Susana SantosMynbaev, KarimBranca, CaterinaMyrick, Michael L.Branda, FrancescoMyrovali, EiriniBrandi, FernandoNa, KyungsuBrasiello, AntonioNabae, YutaBratashov, Daniil N.Nabiałek, MarcinBratskaya, SvetlanaNabil, BouaziziBray, David J.Nabinejad, OmidBreazu, CarmenNabwey, Hossam A.Brechtl, JamiesonNaccache, Mônica FeijóBrenier, AlainNada, AmrBrennan, Anthony B.Nádaždy, VojtechBrescia, RosariaNadeem, SohailBriatico-Vangosa, FrancescoNaftaly, MiraBrichkin, SergeyNag, AbhishekBrigante, MarcelloNagai, MasayaBriganti, MatteoNagamine, KuniakiBrinck, ToreNagane, SatyawanBrintakis, KonstantinosNagappagari, Lakshmana ReddyBriois, PascalNagashima, KazukiBrkic, DejanNaghshvarianjahromi, MahdiBrogi, SimoneNagy, Attila TiborBrook, DavidNagy, EndreBrouzgou, AngelikiNagy, Janos B.Brouzgou, ΑngelikiNagy, LászlóBrown, Wendy ANai, JianweiBrudzisz, Anna M.Naia, MarcoBrühwiler, DominikNaidu, Kadiyala Chandra BabuBruña, SoniaNaik, RamakantaBrunel, DamienNair, Anroop B.Brunel, Damien BernardNair, SelvakumarBrunetti, GiuseppeNaito, ToshioBrunklaus, GuntherNakagawa, NobuyoshiBruno, ElenaNakajima, TakayukiBruno, GiovanniNakamura, YoshiyukiBrycki, BogumilNakanishi, IkuoBryja, LeszekNakashima, NaotoshiBrza, Mohamad AliNakate, UmeshBrzhezinskaya, MariaNakato, TeruyukiBu, LintaoNakayama, TomonobuBubnov, AlexeyNakhchi, Mahdi ErfanianBuchinskaya, Irina I.Nakonieczny, DamianBuckner, Steven W.Nalimov, Anton G.Bucur, BogdanNallal, MuthuchamyBudaeva, Vera V.Nam, InhoBuendia, Carlos DoñateNam, Ki HyunBugaev, AramNam, MinwooBuica, George-OctavianNamburi, DevendraBuiu, OctavianNan, AlexandrinaBujoreanu, Leandru GheorgheNan, HaiyanBukhtiyarov, AndreyNapoleão, Thiago HenriqueBulai, GeorgianaNappini, SilviaBulánek, RomanNáprstková, NatašaBulavchenko, OlgaNaranjo, Fernando B.Bulgariu, LauraNarasimharao, KatabathiniBulow, LeifNarciso, JavierBunge, AlexanderNarducci, DarioBunghez, RalucaNarra, SudhakarBunker, Christopher E.Nashchekin, Alexey V.Buntinx, MiekeNasimuddin, N.Burbelko, AndriyNasiri, NoushinBurg, ArielaNasrollahzadeh, MahmoudBurganos, Vasilis N.Nastasi, BenedettoBurilov, VladimirNastulyavichus, AlenaBurlakov, VictorNasui, MirceaBurlakovs, JurisNataf, GuillaumeBurokur, Shah NawazNatale, PaoloBury, PeterNathanael, A.JosephBury, WojciechNatsuki, ToshiakiBuryi, MaksymNattich-Rak, MałgorzataBusby, YanNaumenko, EkaterinaBuscaglia, VincenzoNaumov, AndreyBuskens, PascalNaumov, Anton V.Busquets, Maria AntòniaNavarro Martinez, Rosa MariaBussolati, OvidioNavarro, AmparoBusuioc, CristinaNavarro, ElenaButkute, RenataNavarro, GabrieleButler, Ian RobertNavarro, María VictoriaButman, MikhailNavarro-Contreras, HugoButt, HaiderNavarro-Quezada, AndreaButt, M.A.Navimipour, Nima JafariButtinoni, IvoNawaz, MuhammadBuzio, RenatoNayak, Manoj KumarByrne, HughNayak, PranatiBystrov, VladimirNazarian-Samani, MasoudCabaleiro, DavidNazemi, EhsanCaballero, AlvaroNazir, GhazanfarCaballero, OlgaNdao, AbdoulayeCabanelas Valcárcel, Juan CarlosNeacsu, Ionela AndreeaCabrera, HumbertoNeamtu, B.V.Cābulis, UģisNebel, ChristophCacciotti, IlariaNechifor, Aurelia CristinaCada, MartinNechifor, GheorgheCadar, OanaNeda, IonCadatal-Raduban, MarilouNedelcu, LiviuCahay, Marc M.Neganova, Margarita E.Cai, LuluNegrea, AdinaCai, NingNegri, R.M.Cai, PeiyuNegro, CarlosCai, YongqingNegut, IrinaCalabria, DonatoNekvindova, PavlaCalamante, MassimoNemes, Norbert M.Calcabrini, AnnaricaNemeth, KarolyCaldas, PauloNemnes, George AlexandruCalina, DanielaNemova, DaryaCallone, EmanuelaNemudry, AlexanderCalucci, LuciaNenadovic, MilosCalvete, MarioNenov, ValentinCalzà, LauraNeophytou, NeophytosCalzada, M.LourdesNergis, BanuCamarillo, RafaelNeri, IgorCamblor, Miguel A.Nersisyan, HaykCameron, DavidNesterenko, Dmitry V.Cametti, CesareNesterenko, Dmitry VladimirovichCaminati, GabriellaNesterova, Oksana V.Camino, FernandoNesvizhevsky, ValeryCammarata, AntonioNesvizhevsky, Valery V.Campajola, MarcelloNetskina, OlgaCampbell, Sawyer D.Neu, JensCampello, PaulaNeuman, Eric W.Campisi, SebastianoNeuman, TomasCampodoni, ElisabettaNeupane, Yub RajCampos, Joaquín MaríaNeverov, Vladimir N.Campos-Martínez, J.Neves, Ana I. S.Canales Vázquez, JesúsNeves, RuiCandiani, GabrieleNevill, Gonzalez SzwackiCanejo, João PauloNevshupa, Roman ACanet-Ferrer, JosepNewcomb, RobertCaniato, MarcoNewton, Graham N.Cannatà, DomenicoNeyman, Konstantin M.Cannilla, CatiaNezamzadeh-Ejhieh, AlirezaCano Luna, ManuelNg, Qin XiangCao, DuxiaNg, Wei LongCao, JunNgo Thi, Yen-LinhCao, KeNgo, Chi-VinhCao, LidongNgo, Ha DuongCao, LingyanNguyen, Anh HCao, QiNguyen, Ha LacCao, ShengNguyen, Ngoc DuyCao, ShunshengNguyen, Nhung H.A.Cao, TunNguyen, Thanh-BinhCao, XudongNguyen, Van DuCao, YanNguyen, Van-ChucCao, YuNiaura, GediminasCaprarescu, SimonaNiazi, SobiaCaprari, ClaudioNicholson, John W.Carabineiro, SóniaNicolais, LuigiCaramês, JoãoNicoletta, Fiore PasqualeCaravella, AlessioNiculescu, VioletaCaravelli, FrancescoNie, LeiCarbajo, JaimeNie, ShengqiangCarbas, RicardoNie, YuanCarbone, FrancescoNieckarz, DamianCarbonnier, BenjaminNiedzialkowski, PawelCardenas-Morcoso, DrialysNieto, AndyCargnoni, FaustoNievergelt, Adrian PascalCaridad, José ManuelNiewa, RainerCarja, GabrielaNifosì, RiccardoCarleer, RobertNikishin, SergeyCarli, StefanoNikolaidis, Alexandros K.Carlin, DanielleNikolić, BiljanaCarlos Sant’Ana, AntonioNikolic, MariaCarlotti, GiovanniNikolova, RositsaCarlotto, SilviaNikos Karathanasopoulos, NikosCarmona, FranciscoNisar, Kottakkaran SooppyCarmona-Ribeiro, Ana M.Nishibori, EijiCarné, ArnauNishimura, AkiraCaro, CarlosNishitsuji, ShotaroCarosio, FedericoNistor, AlexandraCarotenuto, GianfrancoNita, Loredana E.Carrasco, ElisaNitschke, MirkoCarreon, MoisesNitulescu, GeorgianaCarretero, LuisNiu, LiCarroll-Portillo, AmandaNiu, RanCaruntu, GabrielNiu, XiaofengCarvalho, SilviaNiu, ZhiqiangCasado-Coterillo, ClaraNix, WilliamCasarejos, EnriqueNizovtsev, AntonCasarin, MaurizioNobili, Luca G.Caseiro, ArmandoNobre, Marcos Augusto LimaCaseri, WalterNocca, GiuseppinaCasida, MarkNocchetti, MorenaCassani, Maria CristinaNoce, CanioCássio, FernandaNocito, FrancescoCassone, GiuseppeNogales, EmilioCastagna, RiccardoNogueira, AntónioCastaldi, GiuseppeNogueira, DiegoCastaldo, AnnaNoji, MasahiroCastaldo, RacheleNoma, NaokiCastán, HelenaNonnenmann, Stephen S.Castanheira, Elisabete M.S.Noor, NuruzzamanCastell, PereNordblad, PerCastellanos-Gomez, AndresNorek, MałgorzataCastellino, MicaelaNoréus, DagCastelló-Lurbe, DavidNorizan, Mohd NurazziCastellón, Enrique RodríguezNorouzi Banis, MohammadCastillo, OscarNorouzzadeh, PayamCastñeiras, AlfonsoNorton, MichaelCastriotta, Luigi AngeloNosova, Emiliya V.Castro-Muñoz, RobertoNotargiacomo, AndreaCasula, MicheleNotomi, MitsuoCataldi, AmeliaNoubactep, ChicgouaCatalin, IanasiNovikov, Alexander S.Catita, JoséNovikov, Sergey M.Cattoën, XavierNovio, FernandoCauwe, MaartenNovotna, VladimiraCavaliere, EmanueleNovotný, FilipCavallaro, GiuseppeNowak, AdrianaCavalleri, OrnellaNugroho, Ferry Anggoro ArdyCavallini, MassimilianoNunes, MartaCavalu, SimonaNyushkov, BorisCavigli, LuciaO’Rear, EdgarCazacu, Marius MihaiOane, MihaiCazan, CristinaObaidat, IhabCea Mingueza, PilarObermayer, BenediktCecilia, HodurObraztsov, AlexanderCecilia, Juan AntonioObraztsov, Alexander N.Celano, UmbertoOchieng, JosiahCelasco, EdvigeOćwieja, MagdalenaCeleiro, MaríaOdegard, GregoryCelia, FernandesOdriozola, JoséCeliberto, Francesco GiovanniOehler, FabriceCenian, AdamOezkan, SeldaCennamo, NunzioOgawa, ShinichiCepeda, JavierOgawa, ShinpeiČermák, JanOh, DanielCerqueira, FatimaOh, EunkeuCervino, GabrieleOh, HongseokCesco, StefanoOh, JonghyunCeulemans, ArnoutOh, JunhoCevik, EmreOh, PilgunCha, ChaenyungOh, Seung JaeChabert, FranceOh, Won ChunChachkov, Denis V.Ohe, TomoyukiChae, HeeyeopOhmann, RobinChae, Sang YounÖhrström, LarsChaer, IssaOhsedo, YutakaChaitoglou, StefanosOhshiro, TakahitoChalashkanov, NikolaOhta, NarumiChaldyshev, VladimirOhto, KeisukeChalmers, Jeffrey JohnOjha, Gunendra PrasadChamkha, Ali. J.Oka, KengoChampagne, BenoîtOkabe, SeiichiChan, Yau KeiOkada, NarihitoChandra, AbhijitOkamoto, MasamiChandran, RakkiyappanOkazaki, ToshiyaChandrasekar, MurugesanOkazaki, YasumasaChandrasekaran, SwethaOkhay, Olena I.Chandrasekhar, ArunkumarOkitsu, KenjiChang, AiminOkonogi, SiripornChang, ChengOlaizola, Santiago M.Chang, Chia-ChenOlar, RodicaChang, Chi-jungOlar, Rodica MarioaraChang, Chun-ChiehOlchowka, JacobChang, Dong WookOlczyk, PawełChang, FeiOlejnicek, JiriChang, HaixinOlejnik, MalgorzataChang, Hung-ShenOlewnik-Kruszkowska, EwaChang, HyejinOlfs, Hans-WernerChang, LeiOliva, AndrésChang, Sheng-PoOliveira, ElisabeteChang, Yao-FengOliveira, Marcelino MiguelChang, Yi-TsungOliveira, Victor Manuel BarbasChang, Yuan-JenOlivero-Verbel, JesusChang, Yu-ChengOller, Jose AntonioChanthai, SaksitOlmos, DaniaChaput, FrédéricOlugbade, Temitope OlumideCharas, AnaOluwoye, IbukunCharbgoo, FahimehO’Malley, SeanCharisiou, NikolaosOmar, Nur Alia ShehCharles Andrew, DowningOmelyanchik, Alexander S.Charrier, JoelOnaizi, SagheerChartoumpekis, DionysiosOnaka, SusumuChatterjee, AbhijitOnčák, MilanChatterjee, SurajitOndřej, KováříkChaudhry, Mohammed QasimOniga, IlioaraChauhan, Mohinder SinghOnn, Tzia MingChauvin, NicolasOno, AtsushiChava, Rama KrishnaOñorbe, ElviraChavel, PierreOnuta, Marie-ChristineChavez-Angel, EmigdioOnwudiwe, Damian C.Chechenin, Nikolay G.Opra, DenisChecheriţă, Ionel AlexandruOprea, Ovidiu CristianCheedarala, Ravi KumarOrecchioni, MarcoCheepu, Murali MohanOreshonkov, AleksandrChen, HaohongOrgaz, Eva VargasChen, AlexanderOrge, CarlaChen, AnminOrgiani, PasqualeChen, BinOrian, LauraChen, BingdiOrion, ItzhakChen, Bin-HueiOrkoula, MalvinaChen, BoOrlande, Hélcio Rangel BarretoChen, ChengOrmeci, AlimChen, Cheng-LungOrooji, YasinChen, Chia-YunOrozco, JahirChen, ChuanruiOrsi, DavideChen, DapengOrsini, AndreaChen, DazhengOrtega, JuanChen, DongOrtega-Liebana, M. CarmenChen, EnguoOrtenzi, Marco A.Chen, Fang-ChungOrtgies, Dirk H.Chen, FeiOrtiz, Gregorio FChen, George Y.Ortiz-Medina, JosueChen, GuangOsajima, Josy AnteveliChen, GuangmingOsán, JánosChen, GuanjunOscurato, Stefano LuigiChen, GuifengO’shaughnessy, Patrick T.Chen, GuohaiOshikiri, TomoyaChen, His-ChaoOsial, MagdalenaChen, Hone-ZernOsinkin, D.A.Chen, HuanOsipov, AndreyChen, I-Wen PeterOsipov, ArtemChen, JianOsipov, VladimirChen, JianbinOsmieri, LuigiChen, JiangzhaoOsminkina, LiubovChen, Jian-ZhangOsouli Bostanabad, KarimChen, JihuaOstasevicius, VytautasChen, JingdiØstergaard, Martin B.Chen, JunOstrauskaite, JolitaChen, KaiOstrovidov, SergeChen, KaihongOswald, JiriChen, Kai-HuangOszako, TomaszChen, KeO’Toole, Martin G.Chen, Kuan-RenOtsuka, HidenoriChen, KunjiOtsuka, IsseiChen, LeiOtsuki, AkiraChen, Liang-YuOu, JianzhenChen, LianqingOu, XingChen, Li-JunOujja, MohamedChen, LingenOuvrard, AimericChen, LipingOuyang, LiuzhangChen, Lung-ChienOuyang, Xiao-kunChen, MeijieOves, MohammadChen, MengjieOwais, MohammadChen, MingOya, TakahideChen, MingfuOzaltin, KadirChen, MingyuanOzdemir, OrhanChen, Pang-ShiuOztop, HakanChen, Pao ChiP. C. Alves, SérgioChen, QiangPacana, AndrzejChen, RongPacheco, Pedro Manuel Calas LopesChen, ShangshangPacilé, D.Chen, ShaojuanPaczesny, JanChen, ShaoweiPadnya, PavelChen, ShengPadrão, JorgeChen, Sheng-ChiPaek, SanghyunChen, ShunhuaPagano, SergioChen, ShuoPagano, StefanoChen, Shyi-TienPagliara, StefaniaChen, SiPagot, GioeleChen, SuziePai, Chi-FengChen, Szu-yuanPaier, JoachimChen, Tao-HsingPaik, UngyuChen, WangchaoPajootan, ElmiraChen, WanghuaPakhomov, Alexey AChen, WanpingPal, SudiptoChen, Wan-TingPala, NezihChen, Wei-HsiangPalacios-Santander, José MaríaChen, WeihuaPalau, AnnaChen, Wen-ChengPalchoudhury, SoubantikaChen, WenhuaPalego, CristianoChen, Wen-JauhPalei, MilanChen, WenmiaoPalem, RamasubbareddyChen, WenwuPaleo Vieito, António JoseChen, Xiang-BaiPalermo, GiovannaChen, XiaoPalestri, PierpaoloChen, XiuguoPalma, Alessandro L.Chen, XueyePalmer, Robert J.Chen, XuweiPalmieri, ValentinaChen, YanliPalumbo, OrieleChen, Yi-ChengPalyi, GyulaChen, YifangPalys, BarbaraChen, YimingPan, Ci-LingChen, YirenPan, Guo-HuiChen, YuPan, JianChen, Yue-XingPan, LujunChen, YunPan, PanpanChen, Yung-ChungPan, Qing-JiangChen, Yu-WenPan, ShuaihangChen, ZhangjianPan, ZhaoChen, ZhenxiaPan, Zheng ZeChen, ZhijianPana, IulianChen, ZhijunPanagiotis, BousoulasChen, ZhizhongPanajotov, KrassimirChen, ZupengPanchal, HiteshChenevier, PascalePanda, Dillip KumarCheng, Chih-ChiaPanda, Siva S.Cheng, Chih-HsienPandey, PuranCheng, Chung-WeiPandey, SadanandCheng, FengPandith, AnupCheng, GangPang, BoCheng, HanlinPang, GuangchangCheng, Hao-WenPang, HuanCheng, I.Pang, QiangCheng, I-ChungPang, YajunCheng, JinLuoPang, YoonsooCheng, JipingPani, MarcellaCheng, JunyePanich, Alexander M.Cheng, Kong-WeiPanigrahi, Prasanta K.Cheng, QilinPanja, SudiptaCheng, TaoPankratov, AndreyCheng, XiyuePankratov, DenisCheng, YaPankratov, VladimirCheng, YongzhiPanneerselvam, RajapandiyanCheng, YouliangPanniello, AnnamariaCheng, Zhe​Panomsuwan, GasiditChenrayan, SenthilPant, BishweshwarCheong, ByounghoPantano, PaulCheong, In WooPanyukov, SergeyCheraghian, GoshtaspPaolicelli, GuidoChern, EdwardPaolone, AnnalisaChernyak, SergeiPaone, EmiliaChernyshev, VladimirPap, József S.Chernysheva, Maria G.Pap, ZsoltCherunova, IrinaPapa, FloricaChetwynd, Andrew J.Papadakis, StamatiosChhetri, KisanPapadimitriou, Dimitra N.Chhetri, ManjeetPapadimitriou, VassilikiChi, HongPapadopoulos, ChrisChi, YunPapagiannakopoulos, PanosChiadò, AlessandroPapalazarou, EvangelosChianese, SimeonePapamichael, Emmanuel M.Chiang, Kin SengPapan, JelenaChiang, Ming-HsiPapanikolaou, NikosChiantia, SalvatorePapari, Gian PaoloChiarotto, IsabellaPapathanasiou, Thanasis D.Chiesa, EnricaPapatriantafyllopoulou, ConstantinaChigo-Anota, ErnestoPapatzani, StylianiChinappi, MauroPappas, DimitriChinnam, Ajay KumarPappas, George S.Chinnathambi, ShanmugavelPapurello, DavideChiriac, Aurica P.Papynov, EvgeniyChiu, Chih-WeiParadowska-Stolarz, AnnaChiu, Nan-FuParaïso, TaofiqChiu, Tai-ChiaParajuli, DurgaChiu, Wei-HaoParale, Vinayak GChizhik, AlexanderParchin, Naser OjaroudiChladek, GrzegorzParchur, AbdulChlanda, AdrianParfenov, EvgenyChmielarz, LucjanParfenova, LyudmilaChmielewski, TomaszParia, SarbaranjanCho, Deok-YongParida, DambarudharCho, Eun-BumParigi, GiacomoCho, Hak DongParisi, FilippoCho, Jae YoulPark, Chang-SooCho, Jung SangPark, Deok-YongCho, Jun-HyungPark, Gi DaeCho, NamchulPark, GyungsoonCho, Won-JuPark, Hee ChulCho, Young TaePark, Helen HejinChoi, DukhyunPark, HyunChoi, Jae-HongPark, Jea-gunChoi, Jeong RyeolPark, JeewoongChoi, Jong-ryulPark, Jeong YoungChoi, Kwang-YongPark, JinsungChoi, Myong YongPark, Jong HwanChoi, Nag JungPark, Jong-hyeonChoi, Seok KiPark, Jong-MinChoi, ShinhyunPark, JongseungChoi, Sung-SeenPark, Jung HwanChoi, Yong ChanPark, JunyongChoi, YongManPark, Ki SooChoi, Yoon SukPark, Kwi-IlChoi, Young WhanPark, Kyu ChangChoi, YunhyukPark, MinChokkalingam, AnandPark, Min BumChoonara, Yahya E.Park, Min SooChopart, Jean-PaulPark, MiraChou Chau, Yuan-FongPark, SangbaekChou, Kan-senPark, Sang-ShinChou, YachangPark, SeonhwaChoudhuri, IndraniPark, Seung-KeunChoupina, Altino BrancoPark, Su CheongChow, DesmondPark, Sun YoungChow, JamesPark, SungjuneChow, James C.L.Park, SungsuChowdhury, DevasishPark, Tae JooChowdhury, ReazPark, WoojinChoy, Wallace C.H.Park, Yoo SeiChrissafis, KonstantinosPark, Young WookChrissopoulou, KiriakiPark, Young-GeunChristian, MeganneParker, Stewart F.Christoforidis, KonstantinosParodi, AlessandroChristol, PhilippeParra, Duclerc FernandesChristy, MariaParravicini, JacopoChrobak, ArturParrino, FrancescoChronopoulou, LauraParsons, JasonChruszcz-Lipska, KatarzynaParveen, TabassumChu, FuxiangParvinzadeh Gashti, MazeyarChu, HungParvulescu, VioricaChu, S.H.Pascu, Mihail LucianChu, XiakunPascual, Juan PabloChuaicham, ChitiphonPascual, LauraChuang, Cheng-HsinPashchenko, DmitryChubarov, AlexeyPashkin, OleksiyChudoba, DorotaPasini, DarioChumakova, N. A.Pasini, MariaceciliaChun, FengPaskaleva, AlbenaChun, JiongPasqua, LuigiChun, SungwooPasquali, LucaChung, DeborahPassante, RobertoChung, Hyun-JoongPašti, IgorChung, Jae-HyunPasuk, IulianaChung, Jin SukPasupathi, Manoj KumarChung, Ren-JeiPaszula, Józef M.Chung, Sheng-HengPatanè, SalvatoreChurchill, David G.Patel, SanjayChvojka, JiriPatella, BernardoChychłowski, MiłoszPaternò, Giuseppe MariaCiampi, SimonePaternoster, CarloCiardelli, FrancescoPathak, PawanCicchi, StefanoPathak, SaurabhCichosz, MarcinPathan, HabibCid-Samamed, AntonioPatience, GregoryCiepluch, KarolPatil, NagarajCieśliński, JanuszPatil, Santosh SCimalla, VolkerPatra, JagabandhuCiminelli, CaterinaPatra, Jayanta KumarCimpean, AnisoaraPatrick, Dominic LomenzoCimpoesu, FanicaPatrick, ErinCimpoesu, NicanorPatterson, JenniferCini, ElenaPattisson, SamuelCinteza, OtiliaPaul, AnupCiobanu, Romeo CristianPauliukaite, RasaCiobanu, Steluta CarmenPavel, Ioana E.Ciolaciu, DianaPavel, Octavian DumitruCiprian, DaliborPavese, FrancoCiríaco, LurdesPavesi, MauraCiriello, RosannaPavic, LukaCirillo, GiuseppePavitra, EluriCirillo, MatteoPavlík, ZbyšekCirlin, GeorgePawlak, AndrzejCirtoaje, CristinaPawlak, MichałCitro, RobertaPawlikowska-Pawlęga, BożenaCiura, KrzesimirPaz-García, Juan ManuelCivalek, ÖmerPazhamalai, ParthibanClark, CasparPeale, RobertClaverie, AlainPecharromán, CarlosClay, Rudolf TorstenPeddireddy, Karthik ReddyClemens, TorstenPedras, BrunoClement, JoachimPedrini, JacopoClemente, IlariaPedro Acuña, GuillermoClougherty, Dennis P.Pedrosa, Valber A.Cobianu, CornelPedullà, EugenioCochis, AndreaPedzich, ZbigniewCochrane, CédricPeera, Shaik GouseCoclite, Anna MariaPei, ZingwayCoduri, MauroPeiner, ErwinCoelho, João Carlos MesquitaPeixoto De Almeida, MiguelCohen, BoikoPelella, AnielloCohen, Sidney R.Pelicano, Christian MarkCohen, YachinPellegrini, Massimiliano MatteoCoisson, MarcoPellegrino, PaoloColangelo, GianpieroPellejero, IsmaelColeman, Anthony WilliamPeltzer, MercedesColeman, NicholaPeña-Garcia, Ramón RaudelColin, JeromePendharkar, MihirColin, XavierPeng, DengfengCollings, NeilPeng, GuotaoCollins, ScottPeng, HaiyanColombeau, LudovicPeng, HaoColone, MarisaPeng, HaonanColter, PeterPeng, JianComan, CristinaPeng, JunbiaoComan, VasilePeng, LishanComanescu, CezarPeng, RuwenComor, MirjanaPeng, ShupingComuzzi, ClaraPeng, XiaoniuConcepción, PatriciaPeng, YuandongCondorelli, MarcelloPeng, Yung-KangConesa, Juan A.Pengo, PaoloCong, ShanPensa, EvangelinaConnolly, James P.Penuelas, JoséConstantin, DoruPepe, AntoniettaConstantin, LucianPeppel, TimConstantinescu, CatalinPeralta, Juan E.Constantinescu, Catalin-DanielPerche, FédéricoConsumi, MarcoPerduca, MassimilianoConti, BicePerebeinos, VasiliConti, PaoloPereira, Fernando J.Conti, StefaniaPereira, Jose Carlos GarciaCooksey, Gregory A.Pereira, LucianaCoondoo, IndraniPereira, LuísCopolovici, Dana MariaPereira, LuizCoppola, FrancescaPereira, Mauro FernandesCoppola, SaraPereiro, RosarioCoradin, ThibaudPerelomov, Leonid V.Corciovă, AndreiaPeres, Nuno Miguel ReisCormia, Robert D.Perevyazko, IgorCorneliu, MunteanuPerez Del Pino, AngelCoronas Ceresuela, JoaquínPérez Puyana, Víctor ManuelCorpino, RiccardoPerez, RaulCorreia, IlidioPérez-Castrillón, José LuisCorreia, José HiginoPérez-Flores, Juan CarlosCorte-Leon, PaulaPerez-Maqueda, LuisCortés-Arriagada, DiegoPérez-Maqueda, Luis AllanCortés-Hernández, D.A.Pérez-Mendoza, Manuel J.Cosma, PinalysaPerez-Mitta, GonzaloCosta Fernández, José ManuelPérez-Prieto, JuliaCosta, Benilde F.O.Perinelli, Diego RomanoCosta, Carlos MiguelPeriwal, PriyankaCosta, EduardoPerlado, Jose M.Costa, José A.P.DaPerov, Nikolai S.Costa, Jose C.S.Perova, Tatiana S.Costa, Manuel Filipe Pereira Cunha MartinsPerrin, Charles L.Costa, Nuno RicardoPerrin, LaraCosta, PedroPersaud, KrishnaCostales, AuroraPersichetti, LucaCostantino, FerdinandoPersson, AxelCotrufo, MichelePerucchi, AndreaCoulombe, SylvainPerumal, SugunaCoutino-Gonzalez, EduardoPervez, Md. NahidCouto, CristinaPescetelli, SaraCox, AlysiaPessoa, Rodrigo SávioCox, Charles D.Pestryakov, AlexeyCozzoli, P.DavidePetcu, CristianCraciun, Ana-MariaPetelska, AnetaCraciun, Eduard-MariusPeter, LaszloCraciunescu, IzabellPetersen, ElijahCraft, AndrewPetit, TristanCreemers, LauraPetkov, Jordan T.Criado-Gonzalez, MiryamPetr, KorusenkoCrisan, AdrianPetriev, IliyaCrisan, OvidiuPetrik, PeterCristache, Corina MarilenaPetrini, MorenaCristea, DanPetrisor, Alexandru-IonutCristea, Dana MihaelaPetrov, Yuri V.Cristian, PetcuPetrucci, ElisabettaCristiani, PierangelaPetrussa, ElisaCroitoru, Mihail D.Pettinari, GiorgioCruz Fernandes, VirgíniaPetukhov, Anton N.Csete, MáriaPetzelt, JanCtistis, GeorgiosPeukert, WolfgangCuberes, TeresaPezeshki, AtiyeCuesta, RafaelPezoldt, JörgCuevas, FranciscoPezzotti, GiuseppeCui, Bin-BinPfattner, RaphaelCui, HaoPfnür, HerbertCui, WanzhaoPham, Thi HuongCui, YanxiaPham, Tien DucCui, YanyanPham, Tien V.Cui, YongpengPham, Tuan AnhCui, Zhi-HaoPham, Van TrinhCulita, Daniela CristinaPham, Xuan-HungCunningham, ShawnPhan, Manh-HuongĆurković, LidijaPhilipose, UshaCurti, ChristophePhilippe, GalyCurtis, Anthony D.M.Philippopoulos, Athanassios I.Curulli, AntonellaPhilipsen, HaroldCvikel, DeborahPhillippa, BronsonCzeppe, TomaszPhuc, Nguyen H.H.Czernecki, RobertPhung, Thanh KhoaCzigany, ZsoltPiano, DarioCzuba, ZenonPiazza, FabriceCzwartos, JoannaPica, MonicaD, Ranjith KumarPiccirillo, BrunoD. Howe, JoshuaPiccirillo, ClaraD’Agosta, RobertoPickering, EdD’Orazio, FrancoPicollo, FedericoD’souza, AnishaPiedade, Ana PaulaDa Costa Ribeiro, Ana PaulaPielichowski, KrzysztofDa Silva Figueira, Flávio AlbertoPierini, FilippoDa Silva, Luís PintoPiesik, DariuszDa Silva, Pedro RaposeiroPietrzak, PiotrDąbrowska, AgnieszkaPilo, Maria I.Dacarro, GiacomoPilozzi, LauraD’accolti, LuciaPimenov, DanilD’Acunto, MarioPina, Maria PilarDagli, NadirPinchetti, ValerioDahl, Jon E.Pinchuk, AnatoliyDahm, MatthewPineda Rodríguez, TeresaDai, BoPineider, FrancescoDai, HongxingPingitore, NicholasDai, JiyanPinho, SoniaDai, LeiPinna, AndreaDai, NingPinto Da Silva, LuísDai, ShengPiosik, JacekDai, WenxinPiotrowski, PiotrDai, YihuPiprek, JoachimDai, ZhengfeiPiro, BenoîtDai, ZhenghongPisani, MichelaDale, SaraPisarek, MarcinD’Alfonso, LauraPisarkiewicz, TadeuszDambrine, GillesPiskunov, SergeiD’Amico, CiroPissas, MichaelDamin, AlessandroPita, MarcosDammak, LasaadPitchappa, PrakashD’Amora, UgoPitilakis, AlexandrosD’Amore, MarcelloPitto-Barry, AnaïsDana, GingasuPizzoferrato, RobertoDang, AleiPlamper, FelixDang, DongbinPlaninšek, OdonDaniele, SalvatorePlatonenko, AleksandrsDaniele, StephanePlaušinaitienė, ValentinaDaniele, StéphanePlech, AntonDaniels, Kevin MPlesu, NicoletaDanielson, Neil D.Plewa, JulianDanielsson, ÖrjanPliatsikas, NikolaosDaniil, NikitinPliquett, UweDanila, OctavianPlotniece, AivaDantan, AurélienPlummer, John ChristopherDante, SilviaPoater, AlbertDanti, SerenaPobiega, KatarzynaDao, Van-DuongPoccia, NicolaDaoush, Walid M.Poddel’sky, AndreyDargahi, AbdollahPodgurskiy, VitaliDarwish, MohamedPodlewska, SabinaDas, BhaskarPodlipnik, ČrtomirDas, HenaPodor, RenaudDas, MahuyaPodvorica, FetahDas, PoushaliPoiana, MarcoDas, RaselPolanczyk, AndrzejDas, SachindranathPoletaev, Gennady M.Das, Tapan KumarPolicicchio, AlfonsoDas, Tushar KantiPolimeni, AntonioDasari, Bosu BabuPolimeni, John M.Dascalescu, LucienPolimeno, AntoninoDasgupta, IndraPolisetti, VeerababuDashevsky, ZinoviPolitakos, NikolaosDastgeer, GhulamPolitano, AntonioDaub, MichaelPoliwoda, AnnaDaulatabad, NarsimuluPolizos, GeorgiosD’Auria, FrancescoPoljak, MirkoDávalos, Juán Z.Pollard, Shawn DavidDavaran, SoodabehPolo-Parada, LuisDavid, ChristinPolosan, SilviuDavidson, IritPomastowski, Paweł P.Davies, ChrisPominova, DariaDavis, PaulPonomarev, Igor I.Davoodi, Elham (Emma)Pons, JosefinaDavoodi, ShadfarPons, RamonDavydov, RomanPonsinet, VirginieDawidowski, WojciechPontikis, VassilisDawoud, BelalPontremoli, CarlottaDe Almeida, José Manuel Marques MartinsPoodt, PaulDe Andrés, AliciaPoole, Philip J.De Bonis, AngelaPoon, JosephDe Cesare, GiampieroPoornesh, P.De Cumis, Mario SicilianiPop, Ioan M.De Falco, AnnaPop, ViorelDe Falco, GianluigiPopa, AdrianaDe Fazio, DomenicoPopa, AlexandruDe Feyter, StevenPopa, FlorinDe Gouveia, Luis Filipe BaptistaPopa, LacramioaraDe Julian Fernandez, CesarPopa, Vlad T.De Keersmaecker, MichelPopescu, Ileana NicoletaDe Leo, VincenzoPopielarz, RomanDe Lima Nobre, Marcos AugustoPopko, Ewa.De Lorenzo, GiuseppePopov, AnatoliDe Luca, GiorgioPopov, MikhailDe Luca, PierantonioPopov, SergeiDe Maria, LetiziaPopova, MargaritaDe Matteis, FabioPorat, Ze’evDe Matteis, ValeriaPorfireva, A.V.De Mey, GilbertPorotnikova, NataliaDe Moure-Flores, FranciscoPorrelli, DavideDe Nalda, RebecaPortesi, ChiaraDe Oliveira, Helinando PequenoPosati, TamaraDe Oliveira, Severino CarlosPostigo, Pablo AitorDe Padova, PaolaPostnikov, Pavel S.De Padova, Paola IrenePotapov, AndreiDe Paula, Haroldo César BeserraPotgieter, HermanDe Santis, RobertoPothula, Karunakar ReddyDe Santis, SerenaPou, JuanDe Sequeira, César Augusto CorreiaPoudel, Milan BabuDe Sio, LucianoPoulopoulos, PanagiotisDe Souza, SivoneyPoumellec, BertrandDe Stefano, LucaPourrahimi, Amir MasoudDe Tommasi, LucianoPoushali, DasDe Wergifosse, MarcPovstenko, YuriyDe, RanjitPozdnyakov, Aleksandr S.Dec, JanPozdnyakov, Ivan P.Dedkov, G.V.Pozo Perez, DavidDedov, AlexeyPrabhakaran, DharmalingamDefay, EmmanuelPrade, RolfDeferme, WimPradhan, NiharDei, MichelePrado-Gonjal, JesúsDeivanayagam, RamasubramonianPrajzler, VaclavDel Hierro, IsabelPrakash, Annigere S.Delattre, FrançoisPrakash, ChanderDeleanu, Iuliana-MihaelaPrakash, JaiDelgado, Ángel V.Prakash, RaviDelichatsios, Michael A.Pramanik, ArindamDelie, FlorencePramanik, AtinDell’Olio, FrancescoPramanik, SubhamayDella Pergola, RobertoPramanik, TanmoyDella Porta, GiovannaPramann, AxelDell’Anna, Maria MichelaPrashanth, Konda GokuldossDell’Anna, RossanaPrata, José V.Delle Cave, DonatellaPratsinis, Sotiris E.Delouei, Amin AmiriPravica, Michael G.Delville, Jean-PierrePreda, NicoletaDelville, Marie-HelenePredoi, DanielaDemartino, CristoforoPreziosi, ValentinaDemetrescu, IoanaPrida, Victor M.Demin, AlexanderPrieto, AliciaDemontis, ValeriaPrieto, GerardoDendisová, MarcelaPrikhodchenko, Petr V.Deng, HaohuaPrincipe, MariaDeng, QianchunPristovsek, MarkusDeng, QiboPrivitera, AlbertoDeng, TongProchazka, IvanDeniaud, AurélienProchazka, MarekDenisenko, Yuriy G.Pronin, ArtemDenizli, AdilProsa, MarioDenizot, BenoitProtchenko, OlgaDennis, T.John S.Protsenko, Vyacheslav S.Deonikar, VirendrakumarProux, OlivierDeore, Prashant S.Prucnal, SlawomirDequidt, AlainPrudnikova, Svetlana V.D’Ercole, SimonettaPruna, Alina IulianaDerkatch, SvetlanaPruncu, Catalin I.Derosa, PedroPryamikov, AndreyDesai, AamodPrysiazhnyi, VadymDesgranges, LionelPrzesmycki, RafalDespotovic, InesPshenay-Severin, Dmitry A.Deuermeier, JonasPshyk, OleksandrDewapriya, NuwanPsilodimitrakopoulos, SotirisDey, AmritaPtok, AndrzejDhakshinamoorthy, AmarajothiPu, HaihuiDi Bartolomeo, AntonioPu, MingboDi Bella, SantoPucci, Monica FrancescaDi Bitonto, LuigiPuech, PascalDi Bucchianico, SebastianoPuertas, Antonio M.Di Corato, RiccardoPuglia, DeboraDi Costanzo, LuigiPuglisi, RosariaDi Digioacchino, DanielePuigdollers, JoaquimDi Fonzo, FabioPujo, Mariacinta C.Di Franco, CinziaPuli, VenkataDi Ilio, GiovanniPullman, David P.Di Martino, PieraPurcar, VioletaDi Michele, AlessandroPurkait, TaniyaDiaconu, BogdanPurwidyantri, AgnesDiale, MmantsaePussi, KatariinaDiao, ZhuPuszkiel, JuliánDias, CristianoPutero, MagaliDíaz, EvaPutz, Ana MariaDiaz, MarcosPutz, Ana-MariaDiaz, Sebastian AndresPyatakov, Alexander P.Dìaz-Guerra, CarlosPyda, WaldemarDickert, Franz L.Pyrgioti, EleftheriaDidangelos, TriantafyllosQayyum, FaisalDiep, Hung T.Qi, ChengduDietliker, KurtQi, CongDiez, EnriqueQi, DianpengDíez-Pascual, Ana MaríaQi, HaitaoDiko, PavelQi, HongDiliberto, SebastienQi, JiDilonardo, ElenaQi, JianDima, OvidiuQi, JunleiDima, Ştefan OvidiuQi, KezhenDimakis, EmmanouilQi, WeiDimandja, Jean-MarieQi, YanfeiDimitratos, NikolaosQian, DongDimitrijev, SimaQian, DongjinDimos, KonstantinosQian, Jin-yuanDimov, NikolayQian, KunDina, Nicoleta ElenaQian, LihuaDinadayalane, TandabanyQian, TaoDinarvand, SaeedQiang, YingDinca, ValentinaQiao, YancongDinelli, FrancoQiao, YanxinDinesh, Veeran PonnuveluQiao, YuqinDing, DongyanQin, AimiaoDing, FeiQin, DashanDing, GuqiaoQin, DonghuanDing, JingQin, KaiqiangDing, KanQin, LeiDing, MingYeQin, XiaoyingDing, ShichaoQin, YuyueDing, Shinn-JyhQin, ZhaoyeDing, Si-JingQing, YongquanDing, TianpengQingguo, ShaoDing, YichunQiu, FengDing, ZhaoQiu, JingDinh, ToanQiu, KeqiangDinischiotu, AncaQiu, LinDintcheva, Nadka TzankovaQiu, TengfeiDippong, ThomasQu, ManDirisala, AnjaneyuluQu, SongnanDistaso, MonicaQu, XiaozhongDittrich, ThomasQuan, BinDivitini, GiorgioQuaranta, SimoneDixit, MarmQuarta, GianlucaDixon, DavidQuiñones, BeatrizDixon, David A.Quintanilla, AsuncionDjeffal, FayçalQuintino, AlessandroDjellabi, RidhaRabiee, NavidDjordjevic, AleksandarRabilloud, FranckDo Rego, Ana Maria P. L. R. BotelhoRabinovici, RaulDo, Thi-NgaRachwalski, MichałDobrowolski, PiotrRackauskas, SimasDocoslis, ArisRacles, CarmenDodero, AndreaRacovita, RaduDodi, GianinaRada, SimonaDoi, KentaroRadenovic, StojanDoktorova, MilkaRaditoiu, ValentinDolai, SubhashishRadnik, J.Dolci, MarcoRadtke, AleksandraDolenec, RokRadu (Balas), MihaelaDolenko, Tatiana A.Radu, MihaiDolgoborodov, Aleksander YuRadulovic, JovanaDoll, KlausRafailov, Peter MetodievDolle, MickaelRafatullah, MohdDolnik, BystrikRafiee, AtaDomaradzki, JaroslawRafique, SubrinaDomashevskaya, Evelina P.Raghu, Anjanapura V.Domek, GrzegorzRagusa, AndreaDoménech-Carbó, AntonioRahdar, AbbasDomínguez-Reyes, R.Rahimi-Gorji, MohammadDonat, FelixRahman Rashid, Rizwan AbdulDonati, GretaRahman, MahbuburDong, BoweiRahman, Md.Dong, DongmeiRahman, Mohammad MansurDong, HuilongRahman, Mohammed M.Dong, MingdongRahman, MuhibUrDong, QingshunRai, AkhileshDong, ShunRai, Mahendra KumarDong, ShuqingRai, Shyam BahadurDong, WeiRaida, ZbynekDong, XingchenRailean-Plugaru, VioricaDong, XinglongRaimo, MariaDong, YuanRaimondo, MarialuigiaDong, YuanyuanRaizada, PankajDong, ZhaogangRaj, EldonDongare, PratikshaRaj, VinitDongil, Ana BelénRajaji, UmamaheswariDonnadio, AnnaRajan, KalavathyDonnermeyer, DavidRaji, AtchudanDonno, DarioRajnak, MichalDoranehgard, Mohammad HosseinRajput, ShailendraDordevic, Sasa V.Rajput, Vishnu D.Dorofeev, Sergey G.Rajta, IstvanDoroftei, CorneliuRaju, NandhakumarDorozhkin, Sergey V.Ramalingam, S.Dorożyński, PrzemysławRamamurthy, Sai SathishDossi, CarloRamana, E.VenkataDou, HongjingRamanavicius, ArunasDoudin, BernardRamanavičius, SimonasDoutch, JamesRamazani, AliDowon, BaeRamirez Rodriguez, Gloria BelénDrabczyk, AnnaRamírez Sánchez, IsraelDrabowicz, JozefRamirez-Castellanos, JulioDräger, GeraldRamos, DanielDragoman, DanielaRamos, JavierDragoman, MirceaRamos, ManuelDrazic, GoranRan, YangDressel, MartinRana, DipakDrevenšek-Olenik, IrenaRana, MasudDrevet, RichardRanc, VáclavDrewniak, SabinaRangelov, Stanislav M.Drillet, Jean-FrançoisRanger, Christopher MDrmosh, Qasem AhmedRanieri, AntonioDrochioiu, GabiRankin, SteveDror, IshaiRao, AshitDrozdov, AndreyRao, Doddamane Sreenivasamurthy ShankarDrozhzhin, Oleg A.Rao, HengDrygała, AleksandraRao, RahulDu, ChaoLingRaouafi, NoureddineDu, Cheng-FengRapone, BiagioDu, ChunbaoRaposo, MariaDu, ChunfangRaptis, IoannisDu, GuangqingRaptis, Ioannis A.Du, HuihuiRasale, DnyaneshwarDu, MingdeRashad, A.M.Du, PengRashad, Ahmed M.Du, QingyangRasheed, TahirDu, RanRashid, MuhammadDu, WeiRashid, UmerDu, XuRashidi Nodeh, HamidDu, XushengRasmita, AbdullahDuan, ChaoRasoga, OanaDuan, GaigaiRasool, GhulamDuan, JianRasras, MahmoudDuan, QianRasskazov, IliaDuan, XiongboRatautaite, VilmaDuan, ZaihuaRatso, SanderDubey, PoornimaRatto, FulvioDubois, MarcRaty, Jean-YvesDubowik, JanuszRauscher, HubertDuboz, Jean-YvesRauwel, ErwanDubrovskii, VladimirRauwel, ProtimaDubrovskii, Vladimir G.Ravariu, CristianDubrovskiy, Andrey A.Ravichandiran, PalanisamyDucroquet, FrédériqueRavindra, Nuggehalli M.Duda, PiotrRavindranadh, KoutavarapuDudek, MariuszRawtani, DeepakDudek, MirosławRay, ApurbaDudhipala, NarendarRay, VishvaDudin, PavelRayan, Diaa El-RahmanDudina, DinaRayaroth, ManojDuggan, Brendan MRaza, AunDulebenets, Maxim A.Razjivin, AndreiDuliu, Octavian G.Razzak, TowhidurDulski, MateuszRazzaq, Muhammad YasarDumbrava, AncaRebis, TomaszDumitrache, FlorianRebrov, EvgenyDumitru, Andra-CristinaRecchia, SandroDumur, FrédéricRečnik, AleksanderDunaevskiy, MikhailReda, RodolfoDunyushkina, LiliyaReddicherla, UmapathiDuong, Dinh LocReddy, Benjaram Mahipal VijayasimhaDupont, LoicReddy, I. NeelakantaDuraković, BenjaminReddy, Patakota SudarsanaDurand, Jean-OlivierRedkin, ArkadyDurazzo, AlessandraRegel-Rosocka, MagdalenaDurka, KrzysztofReggiani, SusannaDussarrat, DussarratRegmi, BishnuDuta, LiviuRegulacio, Michelle D.Dutta, BiswanathRehberg, IngoDuy, Le ThaiRehman, Muhammad MuqeetDvoranova, DanaReichhardt, CharlesDvurechenskii, AnatolyReineks, EdmundsDwivedi, NeerajReis, Glaydson Simões DosDyck, OndrejReis, Patrícia M.Dymond, MarcusReis, PauloDymshits, OlgaReiss, PeterDzhardimalieva, GulzhianRej, SouravDziadak, BogdanRella, RobertoDzido, GrzegorzRelucenti, MichelaDziedzic, AndrzejRemes, ZdenekDziubek, KamilRen, JiawenDziugan, PiotrRen, JunkaiE., JiaqiangRen, WenEamens, Andrew L.Ren, YataoEbothé, JeanRen, YurongEccles, DavidRen, ZeweiEdeleva, Mariya V.Renedo, Carlos J.Edgar, James H.Rengaraj, ArunkumarEersels, KasperRennhofer, HaraldEfimov, AlexanderReshetenko, TatyanaEfimov, Mikhail NikolaevichReshetilov, Anatoly N.Efimov, Nikolay N.Restaino, Odile FrancescaEfremenko, ElenaReverberi, Andrea P.Efremov, AlexanderReverchon, ErnestoEfthimiopoulos, IliasRevnic, CorneliaEftimie Totu, EugeniaRey, AlejandroEgberts, PhilipReyes Mateo, CarmenEglitis, RobertsReyes, Pavel IvanoffEgorkin, VladimirReyes-López, Simón YobannyEgorov, Alexander V.Reyes-Ortega, FelisaEhrling, SebastianReyhani, AliEhrmann, AndreaRezaee, AbbasEid, Basma M.Rezaei Gomari, SinaEid, KamelRezaei, PejmanEid, Mohamed RabeaRezania, ShahabaldinEkimov, Evgeny ARezvani, Ali RezaEkren, NazmiRhazi, MohammedElaissari, AbdelhamidRhéaume, ÉricElalouf, AmirRhiger, DavidElanthamilan, ElaiyappillaiRho, Won-YeopElashnikov, RomanRhouati, AminaEl-Bana, MohammedRibeiro, Paulo A.El-Bindary, AshrafRibeiro, SusanaEl-Boubbou, KheireddineRicardo, Lopez AntonElemans, Johannes A.A.W.Ricci, Pier CarloEliseev, Andrei A.Richard, CyrilleElizalde-González, María P.Rider, Andrew N.Elkady, MarwaRigosi, AlbertEl-Kemary, Maged AbdeltawabRiha, ShannonEllahi, RahmatRiha, Shannon C.El-Maghrabi, HebaRijckaert, HannesEl-Nadi, YasserRikmann, ErgoElsaid, KhaledRinaldi, AntonioEl-Sayyad, Gharieb S.Rincon Joya, MiryamElshemey, Ashour M.Rincón, LuisElsts, EdgarsRinkevich, AnatoliyElwakeel, KhalidRinkevich, AnatolyElzey, Bennett D.Rinoldi, ChiaraEl-Zohry, Ahmed M.Ríos, FranciscoEmelyanov, Andrey V.Ristau, DetlevEmilio, SardiniRitter, UweEmin, Saim M.Rivero, Pedro J.Emran, MohammedRizal, ConradEndo, TatsuroRizzi, Gian AndreaEnesca, AlexandruRizzo, PaolaEnikeev, NarimanRobert, NewcombEnnen, IngaRoberto Giannuzzi, RobertoEnrichi, FrancescoRobles, EduardoEnríquez Pérez, EstherRocai Cabarrocas, PereEnsafi, Ali A.Rocco Antonio, MontoneEnyashin, AndreyRocco, DavideEom, JonghwaRocco, LuciaErazo, TatianaRoccuzzo, AndreaErcolani, D.Rocha, MarianaErdem, EmreRocha, Thiago LopesEremenko, IgorRochat, SebastienEremeyev, VictorRodehorst, Uta C.Eremin, MikhailRodenbücher, ChristianErmakov, AlexeyRodionova, ValeriaErné, Ben H.Rodrigues, Alisson MendesErrandonea, DanielRodrigues, JoãoErshov, BorisRodrigues, Marco S.Escobar-Alarcón, LuisRodrigues, RaquelEscola, José MaríaRodrigues, SérgioEscorcia, Freddy E.Rodriguez, AngelEscudero, AlbertoRodríguez, José AntonioEscudero, CarlosRodriguez, Paul E.D.SotoEslami, HosseinRodriguez, Roberto GonzalezEsmaeil, Doustkhah HeraghRodríguez-Archilla, AlbertoEsmerian, KarekinRodríguez-Lorenzo, LuisEsperança, José Manuel Da Silva SimõesRodríguez-Lozano, Francisco JavierEspinosa, AnaRodríguez-Pérez, LauraEspinoza-González, RodrigoRodríguez-Yoldi, MaríajesúsEsposito Corcione, CarolaRodygin, Konstantin S.Esposito, RobertoRogachev, AndreyEsposito, SerenaRogovina, S.Z.Espro, ClaudiaRogoziński, TomaszEspuelas, SocorroRogulska, MagdalenaEsquivel-Sirvent, R.Roh, ChanghyunEsquivel-Upshaw, Josephine F.Roh, Kyung-HoEssa, F.A.Roh, TaehyunEs-Souni, MohammedRoizin, YakovEstellé, PatriceRoldán, Juan BautistaEsteves Da Silva, Joaquim C.G.Rolfe, PeterEsteves, TeresaRolo, DoraEstévez, LauraRoma, JoanaEstrany, FrancescRoman, Raul CristianEudald, CasalsRomanato, FilippoEufemi, MargheritaRomano Espinosa, Denise CrocceEugene, FeldshteinRomano, LuciaEugenio, FazioRomanov, AlexeyEum, KiwonRomanovski, ValentinEvans, JulianRomán-Ramírez, Luis A.Evans, KervinRomanyuk, KonstantinEvtugyn, GennadyRomanyuk, Yaroslav E.Evtushinsky, DaniilRombi, ElisabettaEwe, AlexanderRömer, FriedhardExindari, MariaRomero, AlfonsoExposito, Antonio JoseRomero, OscarFaber, KatherineRomero, PedroFabián Gualdrón Reyes, AndrésRomero, PilarFábián, IstvánRomero-Gómez, PabloFabisiak, KazimierzRónavári, AndreaFaccio, GretaRondinella, AlfredoFagadar-Cosma, EugeniaRoose, BartFagiolari, LuciaRoqan, ImanFaia, PedroRosa, Luis GuerraFajín, José L.C.Rosa, PawełFakhrullin, RawilRosania, GustavoFakhrullina, GölnurRosenkranz, AndreasFal, JacekRoshchupkin, Dmitry V.Falabella, EduardoRosi, AntonellaFales, AndrewRosi, MarzioFalkinham, Joseph O.Roslyakov, Ilya V.Fan, Ching-LinRossella, FrancescoFan, HaosenRossi, FedericoFan, JunRossi, LorenzoFan, LiangRossi, MarcoFan, XiaofengRost, ChristinaFan, YuanchengRostovtsev, YuriFan, Yu-JuiRoszak, JoannaFan, YuweiRoszek, KatarzynaFan, ZhaoyangRotaru, AndreiFanciulli, CarloRoth, JohannesFanciulli, MarcoRoth, Wieslaw J.Fang, ChangmingRoualdès, StéphanieFang, GuozhaoRousar, TomasFang, KegongRoussey, MatthieuFang, WenjianRoussigné, YvesFang, YanboRoux, Jean FrançoisFang, Yi-PingRovida, CostanzaFang, ZhiweiRoy, AnuragFangueiro, JoanaRoy, KrishanuFanizza, ElisabettaRoy, RenèFanourakis, DimitriosRoy, SagarFanourgakis, GeorgiosRoy, SwarupFanto, MichaelRoy, Utpal N.Farahani, AliRoychowdhury, SubhajitFaraldos, MarisolRoyo, MiquelFaran, EilonRóżański, Stanisław AndrzejFarfan, NorbertoRozga, PawelFarias, DanielRozhina, ElviraFarina, MarcoRozynek, ZbigniewFarooq, WasifRtimi, SamiFashedemi, OmobosedeRubini, SilviaFasoli, MauroRubio Alonso, FaustoFateixa, SaraRubio Alonso, JuanFattahi, A.M.Rubio-Hernández, Francisco J.Faupel, FranzRuda, HarryFavalli, MicheleRudić, SvemirFavia, GianfrancoRudko, ViacheslavFavre-Réguillon, AlainRudnitskaya, AlisaFavvas, Evangelos P.Rudolf, PetraFayaz, MohammadrezaRudolf, RebekaFecher, Gerhard H.Rudziński, WojciechFederici, SaraRuello, Maria LetiziaFederico, AntonioRüffer, TobiasFedin, MatveyRuffin, PaulFedin, VladimirRuguang, MaFedkiw, Peter S.Rui, KunFedorov, AnatolyRui, XianhongFedorov, Anatoly V.Rui, YukuiFedorov, GeorgyRuiz-Rosas, Ramiro RafaelFei, JinboRukhlenko, IvanFeichtner, ThorstenRumyantsev, SergeyFelba, JanRumyantseva, MarinaFelde, ImreRunka, TomaszFelgueiras, HelenaRuotolo, RobertaFelicetti, SimoneRupani, Parveen FatemehFelip, EudaldRupp, FrankFélix, LuisRusen, EdinaFeliz, MartaRusso Krauss, IreneFelner, IsraelRusso, DaniloFemoni, CristinaRusso, Mario VincenzoFeng, ChuangRusso, PietroFeng, DonghaiRusu, Radu-DanFeng, JiangshanRutkowska-Zbik, DorotaFeng, JianguoRuysschaert, Jean MarieFeng, JijunRuzgas, TautgirdasFeng, JinkuiRyabchikova, Elena I.Feng, LiangliangRyabova, AnastasiaFeng, LiangzhuRyazantsev, MikhailFeng, LiliRybak, AleksandraFeng, QichengRybak, AndrzejFeng, QingliangRybak, ZbigniewFeng, WeiRybolt, TomFeng, WeiyueRydzek, GaulthierFeng, XiangRyms, MichalFeng, XunRysz, JakubFeng, YingRyu, HwaFeng, YixuanRyu, Jae-HongFeng, YongjunRyu, SangwooFeng, YongqiangRyu, Seung YoonFerdov, StanislavRzodkiewicz, WitoldFernandes, Ana CristinaRzymowska, JolantaFernandes, DianaS K Raju, ChakravarthulaFernandes, Mariana Sofia PeixotoSa, BaishengFernandez, CarlosSá, MariaFernández, PalomaSá, PauloFernández-Gutiérrez, MarSabbagh, FarzanehFernandez-Prieto, ArmandoSabban, AlbertFerrando, RiccardoSabirov, DenisFerrara, ElisabettaSabo, RonaldFerrara, Maria AntoniettaSabri, AlamriFerrari, MicheleSachar, Harnoor SinghFerraro, AntonioSacher, EdwardFerraz, Maria PiaSada, CinziaFerreira, Diana P.Sadeghi, FarshidFerreira, Paula M.Sadovnikov, AlexandrFershtat, LeonidSadowska, KamilaFiáth, RichárdSadowski, Jerzy T.Ficai, AntonSadykov, VladislavFicek, MateuszSaeed, MuhammadFierascu, IrinaSaeed, Muhammad AhsanFierascu, Radu ClaudiuSaeid, VafaeiFifere, AdrianSaenko, V.S.Figà, VivianaSafaei, BabakFigueira, RitaSafaei, M.R.Figueira, Rita BacelarSafaei, Mohammad RezaFigueiredo, JoséSafonov, Alexander AleksandrovichFilgueira, CarlySaha, BiswajitFilice, SimonaSahoo, Dipak KumarFilip, AdrianaSahoo, Nanda GopalFilip, JaroslavSahoo, SumantaFilip, PetrSahu, D.R.Filipecki, JacekSahu, RajibFilipescu, MihaelaSaifutdinov, AlmazFilippos, FarmakisSaifutdinov, BulatFilippov, AnatolySaikat, Sinha RayFilippov, AndreySaini, NaurangFilippov, SergeySaito, YouheiFilonova, ElenaSaito, YuikaFink, AlkeSaitoh, HiroyukiFinkel, PeterSajid, MuhhamadFinkler, AmitSajjad, UzairFioravanti, GiuliaSakač, NikolaFiorenza, RobertoSakai, HiromiFiori, FabrizioSakai, JoeFiori, LucaSakai, MasamichiFiorica, CalogeroSakai, ShigekiFiorito, SilvanaSakharova, NataliyaFirestein, KonstantinSakon, TakuoFischer, GeorgSaladino, Maria LuisaFischer, Inga AnitaSalahub, DennisFischer, SzabolcsSalam, Mohamed AbdelFischetti, Massimo VSalameh, ChrystelleFisicaro, GiuseppeSalamon, AndreaFlandin, LionelSalarizadeh, ParisaFleaca, C.T.Salas, GorkaFlieger, JolantaSalauddin, MdFlorea, AdrianSalaün, MathieuFlorea, IleanaSaleh, SayedFlorek, JustynaSalem, Mohamed Z.M.Flores, Ana I.Salerno, MarcoFlorian, CamiloSales, David LéridaFlorian, Paula EcaterinaSalgueiriño, VeronicaFloris, BarbaraSalifoglou, AthanasiosFogacci, FedericaSalimon, AlexeyFogassy, ElemérSalinas-Torres, DavidFohlerová, ZdenkaSalishchev, GennadyFoldes, IstvanSalles, VincentFollink, BartSalmas, Constantinos E.Fologea, DanielSalto-Gonzalez, RafaelFolpini, GiuliaSalur, EminFomin, AleksandrSalvador, Dueñas CarazoFomin, Alexander S.Salvato, MatteoFomin, Vladimir M.Salvatori, StefanoFomina, Marina AlekseevnaSalvestrini, StefanoFominski, V.Yu.Salvi, Anna M.Font, JosepSalzenstein, PatriceFontana, AntonellaSamanta, AvikFontanesi, ClaudioSamaras, IoannisFormánek, PetrSambandam, BalajiForment-Aliaga, AliciaSambath, KarthikForoutan, RaufSambri, LetiziaFortenberry, RyanSamfira, IonelFortmann, CharlesSamokhvalov, AlexanderFortuna, LuigiSamperi, FilippoFortunati, IlariaSan Fabian, EmilioForzan, MicheleSánchez García-Vacas, DanielFosso-kankeu, ElvisSánchez, ManuelFouda, AmrSanchez, Maria LuzFox, MalcolmSánchez-Fernández, Elena M.Fraenzle, StefanSánchez-García, DavidFragoso, AlexSanchez-Soto, LuisFraleoni-Morgera, AlessandroSancho-Garcia, Juan-CarlosFrancàs, LaiaSancini, GiulioFrancia, CarlottaSanctis, ShawnFranco, Camilo AndresSanderson, JosephFrançois Fréty, Roger ThomasSándor, KristyánFrangini, StefanoSandre, OliverFrank, IrmgardSang, YuanhuaFrank, RegineSangaletti, LuigiFrank-Kamenetskaya, Olga V.Sängerlaub, SvenFranz, GerhardSangiorgi, NicolaFranzoni, FerdinandoSangita, KaranjitFrasco, Manuela F.Sankiewicz, AnnaFratalocchi, AndreaSannino, DianaFrath, DenisSansone, FrancescoFrazer, LaszloSantaballa, J.A.Freire, Paulo De Tarso CavalcanteSantagata, AntonioFreitas, MariaSantana, GuillermoFrench, PaddySantoni, AntoninoFresch, BarbaraSantoni, Marie-PierreFresnadillo, DavidSantos, ElizabethFreudenberger, JensSantos, Fernando J.V.Fritsch, DanielSantos, RafaelFroelich, AnnaSantos-Gómez, Lucía DosFrogley, MarkSantoyo-Salazar, JaimeFröhlich, EleonoreSanyal, UdishnuFrontera, CarlosSapelkin, AndreiFrueh, JohannesSapkota, JanakFu, BiaoSaraev, AndreyFu, GengtaoSarafian, ViktoriaFu, LiSarafidis, CharalamposFu, LiansheSaraiva Morais, João PauloFu, ShaozhiSarantopoulou, EvangeliaFu, ShiyuSaravanakumar, KandasamyFu, YangyangSardari, Pouyan TalebizadehFu, YongmingSardo, CarlaFu, YucanŠarić, AnkicaFu, Zongwen.Sarkar, SwagatoFujii, TakenoriSarker, SubrataFujii, TatsuoSarkisov, SergeyFujimoto, KazushiSarkisov, Sergey YuFujita, Ken-ichiSarris, IoannisFujita, SatoshiSarris, Ioannis E.Fujita, ShizuoSartorel, AndreaFujita, ToyohisaSartori, BarbaraFujiwara, AkihikoSaruhan, BilgeFujiwara, YasufumiSarukura, NobuhikoFukami, KimioSarychev, AndreyFuks, LeonSasai, YasushiFulignati, SaraSasajima, YasushiFulvio, Pasquale F.Sasaki, MasahikoFuruta, MamoruSasaki, SatoshiFusco, LauraSastre-Santos, ÁngelaFuso, FrancescoSatender, KatariaGabriele, MagnaSathasivam, SanjayanGabrielli, AlessandroSathish, CIGaczynska, MariaSathish, Clastinrusselraj I.Gafurov, MaratŠatínský, DaliborGagnon, ChristianSato, KazunoriGaiardo, AndreaSato, KimiyasuGaidukovs, SergejsSato, Shin-ichiroGaikovich, Konstantin P.Sato, YukioGain, Asit KumarSatrapinskyy, LeonidGaizauskas, EugenijusSava, B.A.Gajski, GoranSavage, Paul B.Gajtko, OlgaSavilov, Serguei V.Galamboš, MichalSavin, AlexanderGalan-Vidal, Carlos AndresSavin, Alexander V.Galhano, Antonio CarlosSavkin, Konstantin PetrovichGalian, Raquel E.Sawai, JunGaliano, Antonio BernáSawicki, MaciejGalimzyanov, Bulat NSayago, IsabelGalisova, LuciaSayed, MurtazaGalizia, PietroSayyed, Mohammed IbrahimGallardo, IluminadaSazonov, S. V.Gallavardin, ThibaultScandurra, GraziellaGalli, CarloScardaci, VittorioGalliano, SimoneScavini, MarcoGallmeier, Franz X.Schäfer, HelmutGallo, JuanScharber, MarkusGallo, MartaScharff, PeterGallo, MonicaSchatz, JürgenGallo, SalvatoreSchatz, RichardGalvão, Adelino M.Schaufelberger, FredrikGamal Mohamed, MohamedScheibe, BlazejGamarra, LionelScheideler, WilliamGambardella, AlessandroScheiner, SteveGambier, NicolasSchepler, KennethGamiz, FranciscoSchito, Anna MariaGan, LihuaSchlettwein, DerckGan, LuSchlottmann, PedroGan, Yong X.Schmedake, Thomas A.Gan, ZhixingSchmidt, ClaudiaGanazzoli, FabioSchmidt, Wolf-geroGandhi, AshishSchmitt, KatrinGandolfi, MarcoSchmitt, MichaelGandolfi, Maria GiovannaSchmutz, MarcGanesh, RijutaSchneider, ChristofGangrade, AnkitSchneider, JennyGanguly, SayanSchneider, RaphaelGanikhanov, FeruzSchniepp, HannesGan’shina, Elena A.Schohn, HervéGao, ChengchengScholle, MarkusGao, DangliSchoonheydt, RobertGao, DongSchoubben, AurélieGao, FangliangSchreck, MatthiasGao, FengleiSchreiber, IgorGao, JieSchrempel, FrankGao, JinSchröder, SvenGao, JunkuoSchroen, KarinGao, KaixiongSchulz, FlorianGao, LiboSchürmann, UlrichGao, LizengSchutz, JurgGao, MengSchwaminger, Sebastian P.Gao, ShiqiaoSchwandt, CarstenGao, TongSchwarze, MichaelGao, WenchaoSchweizer, ThomasGao, XingchunSciortino, SilvioGao, XingsenScordo, AlessandroGao, XuanScotognella, FrancescoGao, YiScribante, AndreaGao, ZhiyongScrimin, PaoloGao, ZhuangqiangScutaru, Maria LuminitaGao, ZiheSebastián, DavidGaponenko, SergeyŠebestík, JaroslavGaravand, FarhadSecenji, AleksandarGarbarczyk, JerzySecor, Ethan B.Garbovskiy, YuriySecu, MihailGarcía Suárez, Víctor ManuelSecuianu, CatincaGarcía, AnaSedelnikova, OlgaGarcia, Diana VilelaSedlacek, TomasGarcia, GonzaloSedlák, PetrGarcía, HéctorSeenu, RaviGarcía, LucíaSegre, Carlo U.Garcia, Maria Isabel DiezSeibt, MichaelGarcia, RicardoSęk, GrzegorzGarcía-Antón, JordiSekar, KarthikeyanGarcia-Benett, AlfonsoSekar, SankarGarcia-Deibe, AnaSekatski, SergueiGarcía-Fresnadillo, DavidSekhar, JainageshGarcia-Garcia, Francisco J.Sekine, TomohitoGarcía-Giménez, RosarioSelim, SamyGarcia-Granada, Andrés AmadorSelishchev, DmitryGarcia-Lekue, AranSelke, MatthiasGarcía-Ramos, Juan CarlosSemaltianos, Nikolaos G.Garcia-Salaberri, Pablo A.Semenova, Irina V.Garcia-Sanchez, FelipeSementa, LucaGarcia-Torres, JoséSemeraro, PaolaGarcía-Vargas, Jesús ManuelSemisalova, AnnaGardelis, SpirosSemlitsch, BernhardGardner, James M.Sen Gupta, ArnabGareev, KamilSen, SujatGareeva, ZukhraSenel, MehmetGargiulo, ValentinaŞenilă, MarinGarifullin, RuslanSennato, SimonaGaroli, DenisSenthil, ChenrayanGaroufalis, ChristosSeo, Dae-ShikGarroni, SebastianoSeo, Hyung KeeGarrot, DamienSeo, HyunwoongGartia, Manas RanjanSeo, InseokGarzón Manjón, AlbaSeo, Sang-WooGatin, AndreySeol, Myeong-LokGatti, AntoniettaSeong, GimyeongGatti, GianlucaSepulveda, AbdonGatti, TeresaSepulveda, BorjaGatto, EmanuelaSequeira, César A.C.Gauden, PiotrSequino, LuigiGauden, Piotr A.Serbezeanu, DianaGaumet, Jean-JacquesSerda, RitaGautam, BhojSerdyukov, VladimirGautam, SiddharthSerebryannikov, Andriy E.Gavarri, Jean-RaymondSerenyi, MiklosGavrus, AdinelSeres, JozsefGawinkowski, SylwesterSergeyev, VladimirGawlik, WojciechSerikov, ArkadyGazda, MariaSerov, PavelGazzoli, DeliaSerpilli, MicheleGbadamosi, AfeezSerpooshan, VahidGe, GangSerrà, AlbertGe, Jun CongSerrano, AngelGe, MingzhengSerrano, GiuliaGe, ShengboSerša, IgorGębara, PiotrSevilla, MartaGebeshuber, Ille C.Sgarbossa, PaoloGeletii, YuriiSgreccia, EmanuelaGenç, AzizSgroi, Mauro FrancescoGennari, OriellaShaban, MohamedGenovese, CarloShaban, SamyGenovese, Kenneth J.Shabatina, TatyanaGeorge, GibinShabbir, BabarGeorge, Sajan DanielShah, Nehad AliGeorgii, RobertShah, ZahirGeorgin, JordanaShahabuddin, SyedGeorgiou, EmmanuelShahid, MuhammadGeorgiou, Emmanuel P.Shahid, Muhammad KashifGer, Tzong-RongShahin, VictorGerasimov, Evgeniy Yu.Shahzad, Muhammad WakilGerasymchuk, Yuriy S.Shaik, Gouse PeeraGerislioglu, BurakShaik, Mohammed RafiGeso, MoshiShaikh, ShoyebGesuele, FeliceShaikh, Shoyebmohamad FGeta, David A.Shainsky, JannaGevorgian, AshotShalev, GilGhalambaz, MohammadShan, YangGhamsari, Morteza SasaniShang, JunGhareeb, MosadShang, ZhihaoGhasali, EhsanShanmukaraj, DevarajGhashang, MajidShao, ChangyuGheorghiu, EugenShao, DongGheorghiu, FeliciaShao, LongquanGherlone, EnricoShao, PanlinGhibaudo, GérardShao, TaoGhica, DanielaShapovalov, VictorGhidelli, MatteoShapter, JoeGhilane, JalalSharifuzzaman, MdGhislandi, Marcos GomesSharma, AshutoshGhisu, TizianoSharma, BharatGhodake, GajananSharma, DineshGhodsi, MojtabaSharma, GauravGhofraniha, NedaSharma, Piyush SindhuGholami, PeymanSharma, SandeepGholampour, SeifollahSharma, Shiv K.Ghomi, MahmoudSharma, Suchinder K.Ghorbani-Asl, MahdiSharma, Tata Sanjay KannaGhosh, BiplabSharpe, PaulGhosh, SabyasachiShaula, AliaksandrGhoshal, SushantaShavaleev, Nail M.Ghuge, SandipShaw, SachinGhyngazov, Sergey AnatolievichShcharbin, DzmitryGiancane, GabrieleShcherbakov, AlexanderGiannakas, ArisShchetinin, IgorGiannazzo, FilippoShchyglo, OlegGiannopoulos, Georgios Ι.Shearer, CameronGianolio, ElianaShehzad, KhurramGiapintzakis, John (Ioannis)Shellaiah, MuthaiahGierczyk, BłażejShen, FangxiaGierszewska, MagdalenaShen, HaoGietl, Hanns A.Shen, JialongGîfu, Ioana CătălinaShen, JunyuGil, BarbaraShen, RuiqingGil, BernardShen, WenfeiGil, IgnacioShen, XiaochenGil’denburg, VladimirShen, XiaopingGilarranz, Miguel AngelShen, YanbaiGil-Castell, OscarShen, Yong-miaoGiner-Tarrida, LuísShen, YuxiaGingaşu, DanaShenashen, Mohamed A.Ginzburg, PavelShende, SudhirGiorgini, LorisShenderovich, Ilya G.Giovanela, MarceloShendrik, RomanGiovannetti, RitaSheng, JiangGiovannini, TommasoSheng, LiyuanGirard-Egrot, AgnesSheng, ZonghaiGireesha, Bijjanal JayannaSheremet, MikhailGirolami, MarcoSherman, EvgenyGironi, LucaSherrell, PeterGirtan, MihaelaShestakov, MikhailGîrțu, Mihai A.Shestopalov, Michael A.Gitsas, AntonisSheta, Sheta M.Gitsov, IvanShevchuk, Igor V.Giudici, MauroShevelkov, Andrei V.Gizdavic-Nikolaidis, MarijaShi, BingyangGładysz-Płaska, AgnieszkaShi, De-LiGlahn, RayShi, DongliangGlasovac, ZoranShi, KewenGlatter, OttoShi, LingyanGlavina, DomagojShi, MiusiGlei, MichaelShi, Shih-ChenGlotov, AleksandrShi, Wei‐LongGłuchowski, PawełShi, WenGlukhova, Olga E.Shi, XifengGlushkov, Alexander V.Shi, YanshuGnade, Bruce E.Shi, YongqiangGnyba, MarcinShi, YujunGobbo, MarinaShi, ZhichengGoda, Emad S.Shi, ZhiqingGodinho, Maria HelenaShi, ZujinGodoy, AndresShieh, Tzong-MingGogos, AlexanderShih, PohsinGogova, DanielaShima, HiroyukiGoh, Kheng-LimShimada, ToshihiroGökce, BilalShimizu, TakahisaGöktaş, AbdullahShin, Chae-HoGoldberg, Margarita A.Shin, Eun JuGoldfarb, IlanShin, HyeyoungGoldhawk, Donna E.Shin, Jun-HwanGoldmann, MichelShin, MyunghunGoldthorpe, Irene A.Shin, NaechulGolewski, Grzegorz LudwikShin, SunmiGolgovici, FlorentinaShinde, Sandip S.Golub, IgorShinde, Surendra KrushnaGolubina, Elena V.Shiotari, AkitoshiGolubkina, Nadezhda A.Shirakawa, NaokiGómara, María JoséShiryaev, Andrey A.Gombár, MiroslavShirzadi, AmirGomes, Ana C.Shkir, MohdGomes, Ana TeresaShkirin, AlexeyGomès, SéverineShklover, JenyGómez García, Carlos J.Shlimas, DmitriyGómez Villalba, Luz StellaShlyakhtin, Oleg A.Gómez, MontserratShoji, YuyaGómez-Cerezo, NatividadShokouhimehr, MohammadrezaGomez-Sainero, Luisa MariaShorokhov, AlexanderGómez-Soberón, Jose ManuelShorstkii, IvanGoncalves, AlexandreShorubalko, IvanGonçalves, Helena M.R.Shpacovitch, VictoriaGonçalves, M.Sameiro T.Shrestha, LokGondek, LukaszShrestha, Nabeen KGong, BowenShtepliuk, IvanGong, HaiboShu, XuefengGong, PengjianShu, YangGong, WanbingShuba, Mikhail V.Gong, YinyanShubhakar, KalyaGontrani, LorenzoShuck, ChristopherGonzález, Florenci V.Shuguang, BiGonzález-Alonso, DavidShuklov, IvanGonzález-Calbet, José M.Shulga, YuriyGonzález-Curbelo, Miguel ÁngelShun, YaoGonzález-Díaz, BenjamínShurpik, DmitriyGonzález-Díaz, OscarShvartsman, VladimirGonzalez-Garcia, JorgeShvidchenko, Aleksandr V.Gonzalez-Gutierrez, JoaminShyichuk, AleksanderGonzalez-Leal, J.M.Si, Ping-ZhanGonzález-López, Tomás JoséSibatov, Renat T.González-Vila, ÁlvaroSicard-Roselli, CécileGoodarzi, MarjanSiddiqui, Farooq RiazGorb, Leonid G.Siebert, JulienGorczynski, AdamSiekierka, AnnaGordano, AmaliaSieni, ElisabettaGordon, JeffreySieradzki, AdamGórecki, KrzysztofŠiffalović, PeterGorjanc, MarijaSigaev, VladimirGorji, Nima E.Sigalapalli, Dilep KumarGorkunov, Maxim V.Sigle, WilfriedGórny, KrzysztofSignore, GiovanniGorshkov, NikolaySignorini, RaffaellaGorte, Raymond J.Sigusch, Bernd W.Goryacheva, IrinaSikora, JanuszGościańska, JoannaSilina, YuliyaGośliński, TomaszSilkin, Viatcheslav M.Goswami, NirmalSilva Filho, EdsonGotić, MarijanSilva, AnaGoto, YosukeSilva, BrunoGottwald, AlexanderSilva, Maria JoaoGoula, Maria A.Silva, Nuno HélderGoyal, AmitSilva, SóniaGozu, ShinichirouSilva, Teresa Moura EGraceffa, RitaSilva, Tiago AlmeidaGracia Lostao, Ana IsabelSilvain, Jean-FrançoisGracia Pinilla, Miguel AngelSilveira, Nádya P.DaGracin, DavorSilvestre, Samuel M.Gradov, Oleg M.Silvestri, BrigidaGradov, Oleg V.Silvestri, DanieleGraf, MarkusSilyukov, OlegGraham, JosephSim, SangwanGrajewski, JakubSimanjuntak, Firman MangasaGrande Tovar, Carlos DavidSimari, CataldoGrande, AntonioŠimek, MilanGrasset, FabienSimeone, Felice CarloGrassi, AlfonsoSimeonov, VasilGrassia, VincenzoSimha Martynkova, GrazynaGrätz, SvenSimion, CristianGravel, EdmondSimion, MonicaGray, StephenSimmonds, Paul JGraziano, GiuseppeSimões, RogérioGrazioli, CesareSimoes, SandraGrenzer, JörgSimončič, BarbaraGrgur, BranimirSimonenko, E.P.Gric, TatjanaSimonin, LoicGriffiths, MalcolmSimonsen, IngveGrijalvo, SantiagoSimserides, ConstantinosGrill, RomanSing, Swee LeongGrillo, AlessandroSingh, Ajaya KumarGriol, AmadeuSingh, Arun K.Grishin, MaximSingh, CharanjeetGrisolia, JeremieSingh, DavidGristina, RobertoSingh, Deepak PratapGrivel, Jean-ClaudeSingh, GurvinderGrob, KoniSingh, JaiGróf, GyulaSingh, Jaspal B.Gromov, Dmitry G.Singh, KeharGrosdidier, ThierrySingh, MahiGross, Andrew JamesSingh, NeenuGrosseau, Philippe H.Singh, PardeepGrubel, KatarzynaSingh, PriyankaGrueso, EliaSingh, RaginiGrugeon, SylvieSingh, RahulGrumezescu, Alexandru MihaiSingh, RanbirGrumezescu, ValentinaSingh, RituGruttadauria, MichelangeloSingh, SandeepGrybos, JoannaSingh, SimaGrym, JanSingh, SomnathGrynko, OleksandrSingh, SubhashGrza̧dka, ElzbietaSingh, SurenderGrzegorz, Nałȩcz-JaweckiSingh, VeenaGrzesiak, JakubSingh, Vidya NandGrzyb, JoannaSingh, YashpalGrzyb, TomaszSinha-Ray, SumanGu, BingSinyukov, AlexanderGu, ChengSipos, PálGu, GuoqiangSiska, FilipGu, JiangjiangSivaramapanicker, SreejithGuan, BinSivashanmugan, KundanGuan, JingqiSiwińska-Ciesielczyk, KatarzynaGuangjiu, ZhaoSkillen, NathanGubaidullin, Aidar T.Skládal, PetrGubarev, Fedor AlexandrovichSkotak, MaciejGubitosa, JenniferSkotnicki, TomaszGuda, Alexander A.Skouras, Eugene D.Gudkov, SergeySkunik-Nuckowska, MagdalenaGudmundsson, Jon TomasSkwarek, EwaGudmundsson, VidarSlama, PetrGueorguiev, Gueorgui KostovSlimani, YassineGuerrero-Pérez, M.OlgaSlodek, AnetaGuerrero-Sanchez, CarlosSlomkowski, StanislawGugliuzza, AnnarosaSlugeň, VladimírGui, XuchunSmarandache, AdrianaGui, YingangSmått, Jan-HenrikGuidi, PatriziaSmet, PhilippeGuidotti, MatteoSmiljanić, MilutinGuilemany, Josep MariaSmirnov, Alex I.Guillon, MarcSmirnov, VladimirGuimarães, Freddy FernandesSmith, Paul F.Guizard, StephaneSmolyaninov, IgorGul, TazaSmułek, WojciechGulbinas, VidmantasSmulko, JanuszGulino, AntoninoSmutok, OlehGull, MaheenSnow, Nicholas H.Guller, AnnaSo, HongyunGultom, Noto SusantoSoares, JulioGundlach, LarsSoares, NelsonGuney, DurduSoares, SofiaGunji, TakaoSoavi, GiancarloGun’ko, Volodymyr MoiseevichSobczak, IzabelaGuo, BaolinSobkowiak, AndrzejGuo, ChangfaSobola, DinaraGuo, DabinSobolev, AlexanderGuo, DandanSobolev, Nikolai AndreevitchGuo, Dong-ShengSobolev, VladimirGuo, HuSobotta, ŁukaszGuo, JiminSocaci, CrinaGuo, JingSocol, MarcelaGuo, JinxinSocoliuc, VladGuo, JunSodhro, Ali HassanGuo, KaiSofos, FilliposGuo, LeiSofronov, Anton N.Guo, LinSoga, TetsuoGuo, RuiqianSohn, Jeong-SooGuo, WeihongSoifer, Viktor A.Guo, XiaoyangSoin, NavneetGuo, XinSokolov, MaximGuo, XingmingSokolov, PetrGuo, XingzhongSolanki, RajGuo, YaoSolcova, OlgaGuo, YongSolin, NiclasGuo, YongshengSolis, JavierGuo, YufengSolodovnik, MaximGuo, Zhan-HuSołoducho, JadwigaGupta, GajendraSolovieva, AnastasiyaGupta, ‪NikeshSome, SurajitGupta, SanjuSommer, SylwesterGupta, TusharSomu, PrathapGurban, Ana-MariaSon, Jong YeogGusev, AlexanderSon, Seung UkGuskova, OlgaSonaimuthu, MohandossGut, KazimierzSong, Can-LiGuthold, MartinSong, HyungjunGutiérrez, MarioSong, JizhongGutierrez-Sanchez, CristinaSong, KeweiGuttmann, PeterSong, LiangGutzov, StoyanSong, QinghuaGuyot, BorisSong, WeijieGuzmán Ruiz, RocíoSong, XuepingGwon, Jae-GyoungSong, XuezhiHa, Chang-SikSong, YuHabaue, ShigekiSong, YujunHadjadj, AomarSoni, VishalHagège, AgnèsSood, AnkurHahm, EunilSoon, Jong JeongHahm, Sung HoSoran, Maria-LoredanaHahn, EricSorg, HeikoHahn, Jae RyangSorrentino, AndreaHahn, Yoon-BongSorrentino, LucaHaidar, SajjadSotgiu, GiovanniHajra, SugatoSotillo, BelénHakeem, Deshmukh AbdulSotiropoulos, SotirisHaldolaarachchige, NeelSoto, PaulHaldorai, YuvarajSou, KeitaroHaleem, AbdulSoukup, KarelHall, SteveSoultati, AnastasiaHamam, YskandarSpadaccini, RobertaHamamoto, YujiSpadaro, Maria ChiaraHamawand, IhsanSpanu, DavideHammad, SeddikSparnacci, KatiaHampp, NorbertSpeck, James.S.Hampp, Norbert A.Speliotis, ThanassisHan, AijunSperanza, GiorgioHan, ChaoSpieß, LotharHan, DandanSpina, MassimoHan, Dong-PyoSpiridigliozzi, LucaHan, Dong-WookSpirk, StefanHan, GenquanSpivak, YuliaHan, GuangSpizzo, FedericoHan, Guo ZhiSportelli, Maria ChiaraHan, HeyouSrdic´, VladimirHan, Jae-HeeSreekanth, Kandammathe ValiyaveeduHan, Jae-HoonSridharan, KishoreHan, JingquanSriram, BalasubramanianHan, Joong TarkSrivastava, G.P.Han, WeiSrivastava, VarshaHan, WeizhongSrivastava, Yogesh KumarHan, WenboStaal, JensHan, XiaolongStadler, FlorianHan, YingchaoStagi, LuigiHan, ZhanghuaStamate, EugenHanada, NobukoStamatin, IoanHanaor, DorianStamatis, HaralambosHanawa, TakehisaStamopoulos, DimosthenisHandke, BartoszStamplecoskie, KevinHang, ChunjinStampor, WaldemarHaniu, HisaoStan, CristinaHanna, HarantStan, MirunaHannachi, EssiaStanisz, BeataHansen, WolfgangStanton, Eric J.Hantanasirisakul, KanitStaroń, PawełHanusa, TimothyStarovoitov, PavelHao, DerekStary, ZdenekHao, GaziStashkevich, Andrey A.Hao, LiStasiak, AnnaHao, QingStaszak, KatarzynaHao, ShuweiStaszewski, TomaszHao, YongStathatos, EliasHao, ZhifengStathi, PanagiotaHaponiuk, Józef T.Stathopoulos, SpyrosHaque, FaiazulStavarache, IonelHarale, Namdev ShankarStavitskaya, AnnaHaramus, Vasyl M.Stawicka, KatarzynaHarcharras, MohamedStedman, KennethHarhay, KhrystynaStefan, MariusHarja, MariaStefan-Andrei, IrimiciucHarker, AnthonyStefaniuk, IreneuszHarmandaris, VagelisStefano, Vecchio CipriotiHarničárová, MartaStefanowicz, PiotrHaro, MartaStehlik, StepanHarrison, Nicholas M.Stejskal, VaclavHarrison, Roger GStellato, FrancescoHartmann, UweStenzel, OlafHartnagel, HansStepanov, Gennady V.Haruyama, JunStepanov, SergeyHarzandi, Ahmad M.Stephanovich, VladimirHasal, PavelStergiopoulos, ThomasHasan, MurtazaStergiou, AnastasiosHasegawa, MakotoStetsyshyn, YurijHaselmayr, WernerStiemer, MarcusHashemi, Seyyed AlirezaStiharu, IonHashemi-Dezaki, HamedStine, Keith J.Hashidzume, AkihitoStipa, PierluigiHashimoto, MakotoStirner, ThomasHashimoto, ShunsukeStiufiuc, RaresHashimoto, TakeshiStobiecka, MagdalenaHasnat, Mohammad A.Stoddart, PaulHassan, Haes AlhelouStodolak, EwaHassanien, AbdouStogniy, Marina Yu.Hassanzadeh-Aghdam, Mohammad KazemStoikov, Ivan IvanovichHatada, RurikoStoilova, OlyaHathout, RaniaStolarczyk, AgnieszkaHattori, Azusa N.Stolarczyk, KrzysztofHattori, ToshiakiStoleriu, StefaniaHatzikraniotis, EvripidisStoller, MarcoHausmann, MichaelStolojan, VladHawash, ZaferStoof, H.T.C.Hayakawa, YasushiStornaiuolo, MarianoHayashi, FumitakaStorsberg, JoachimHayashi, TakuyaStoukatch, SergueiHayashi, YoshihitoStoyanova, AntoniaHayat, AsifSt-Pierre, JeanHayrapetyan, D.B.Strakosas, XenofonHazer, BakiStrambini, LucanosHe, Chun-HuiStratakis, EmmanuelHe, DelongStraumal, Alexander B.He, FengŠtrbac, SvetlanaHe, GangStrehle, SteffenHe, GuanjieStreimikiene, DaliaHe, HaishengStriani, RaffaellaHe, HaiyongStrikhanov, MikhailHe, HeyongStringaro, AnnaritaHe, HongStrojny, BarbaraHe, HongmingStrout, Douglas L.He, JieStruis, RudolfHe, JihuanStrunskus, ThomasHe, LiangcanStrzałkowski, KarolHe, LingfengStueckle, Todd A.He, MaoshuaiSturini, MichelaHe, MengStürup, StefanHe, ShuaimingStylianakis, Minas M.He, ShuijianSu, HanquanHe, TingchaoSu, MingHe, XuexiaSu, XinHe, YongSu, YuHe, ZhengranSu, YuanjieHegedűs, CsabaSu, YueHegedűs, LászlóSu, YunlanHeimann, RobertSu, Yu-ShengHeinrich, BenoîtSu, Yu-WeiHeiß, AlexanderSu, ZhaohongHeiss, WolfgangSu, ZijianHeitzmann, MarieSuárez, IsaacHelal, AasifSuarez, TeresaHeljak, MarcinSuárez-García, SalvioHeller, William T.Suaria, GiuseppeHelseth, Lars EgilSubbotin, KirillHemmati, ShohrehSubhan, Md AbdusHendrix, JelleSubrahmanyam, ChallapalliHeng, NicholasSubramanian, PalaniappanHeo, JunseokŠubrt, JanHeremans, JeanSuchaneck, GunnarHermawan, AnggaSuchikova, YanaHermida, Jesús BarrioSuffczyński, JanHernández Rodríguez, Miguel AndrésSuffritti, Giuseppe BaldovinoHernandez, AgileoSugahara, TakuyaHernández, IgnacioSugihara, AtsushiHernandez, Jaime J.Suh, Jun MinHernández-Cifre, José GinésSukumaran, VijayHernández-Pérez, IvánSüle, PéterHernando, A.Suleiman, Rami K.Herrmann-Geppert, IrisSulejczak, DorotaHerth, EtienneSulka, GrzegorzHerynek, VítSulka, Grzegorz DariuszHettiarachchi, SajiniSultan, Mohamed ThariqHeuskin, Anne-CatherineSumboja, AfriyantiHevus, IvanSumiya, MasatomoHeydari Gharahcheshmeh, MeysamSun, BaiHidechi, UsuiSun, BinHildner, RichardSun, BingHill, JonathanSun, BoHimly, MartinSun, Chong-DeHincapie, RafaelSun, HaidingHinterstoisser, BarbaraSun, HainanHippler, RainerSun, HanjunHiroyukuki, HaradaSun, HuiHirtz, MichaelSun, JiaHitoshi, HabukaSun, JianHix, GarySun, JiazhenHng, Huey HoonSun, JieHo, Wen-JengSun, JihongHoang, Tuan K.A. (Tom)Sun, JingyaoHobbie, Erik K.Sun, JunweiHoch, ConstantinSun, Jun-yiHock Ng, SoonSun, Kien-WenHöckner, MartinaSun, LemingHodoroaba, Vasile-DanSun, LichengHoenders, Bernhard J.Sun, LingHoffmann-Eifert, SusanneSun, LipingHöfft, OliverSun, MengtaoHoflich, KatjaSun, QiHofmann, RalfSun, QianHofsäss, HansSun, TaoHogan, ConorSun, TingHoller, StephenSun, WenhongHolst, BodilSun, XiankaiHolze, RudolfSun, Xue-FeiHolzer, ChristofSun, XupingHolzman, Jonathan F.Sun, YanmeiHomaeigohar, ShahinSun, YiHomocianu, MihaelaSun, YilinHoncová, PavlaSun, YuanyuanHonda, YosukeSun, Yun-LuHong, BinSun, ZhaoqiHong, Bo-YoungSun, ZhaoxiHong, JiwooSun, ZhiweiHong, JohnSunada, KayanoHong, LiangSundarrajan, SubramanianHooper, Douglas C.Sundmacher, KaiHooper, KatherineSundqvist, BertilHoover, BrianSung, WonmoHorakova, JanaSünkel, KarlheinzHörning, MarcelSurblé, SuzyHorszczaruk, ElżbietaSurdo, SalvatoreHorvat, GabrijelaSurdu, Vasile-AdrianHorwat, DavidSurmenev, RomanHoseinzadeh, SiamakSuryanarayana, PhanishHoskins, ClareSutka, AndrisHosono, NobuhikoSuturin, SergeyHossain, Md FarukSuwardi, AdyHossain, Md. KhalidSuzuki, KenHosseinaei, OmidSuzuki, MadokaHosseini-Bandegharaei, AhmadSuzuki, NorihiroHotovy, IvanSuzuki, ToyofumiHou, JunSvancara, IvanHou, JungangSverdlov, ViktorHou, XianghuiSvetlichnyi, Valery AHou, XiaojiangSvetogorov, RomanHou, YuchenSvetovoy, VitalyHou, ZhilingSvirko, YuriHou, ZhipengŠvirskis, ŠimonsHouska, JiriSvoboda, JanHousley, Robert M.Svoboda, LadislavHowell, BobSwaraj, SufalHowell, Mark C.Swiderek, PetraHoye, Robert L.Z.Świercz, RafałHrenar, TomicaŚwietlicka, IzabelaHristov, Jordan YankovŚwietlik, RomanHristu, RaduSybachin, Andrey V.Hryniewicz, TadeuszSydoruk, OleksiyHseinat, Emad AlSyed, NituHsiang, Hsing-ISyllaios, A.J.Hsiao, Fu LiSylvestre, AlainHsiao, Hui-HsinSynoradzki, KarolHsiao, I-LunSyrgannis, ZoisHsiao, Kai LongSyrrokostas, GeorgeHsiao, Wen-TseSysoev, VictorHsiao, Yu-ChengSyzgantseva, Olga A.Hsiao, Yu-JenSzabó, GyörgyHsieh, Chih-ChunSzabó, TamásHsieh, ShuchenSzabó-Bárdos, ErzsébetHsu, FuyinSzałowski, KarolHsu, Fu-YinSzczepanowicz, KrzysztofHsu, Heng-TungSzcześ, AleksandraHsu, Jin-CherngSzczęśniak, BarbaraHsu, Julia W.P.Szczęśniak, DominikHsu, Shih-ChiehSzéchenyi, AleksandarHsu, Yung-JungSzedlak, RolfHu, AnmingSzekely, GyorgyHu, DaodaoSzekeres, Anna MariaHu, GuobiaoSzeleszczuk, ŁukaszHu, GuoqingSzerb, Elisabeta IHu, HanSzewczyk, Piotr KrzysztofHu, HuangSzewczyk, RomanHu, JiangtaoSzindler, MarekHu, JiansheSzłyk, EdwardHu, JieSzmytkowski, JȩdrzejHu, JingtianSznitko, LechHu, KeqiangSzoboszlai, NorbertHu, KuanSzperlich, PiotrHu, LiangSzukalski, AdamHu, LiangshengSzultka-Młyńska, MałgorzataHu, LiuyongSzybowicz, MiroslawHu, Lun-HuiSzychowski, KonradHu, NaSzyk-Warszyńska, LiliannaHu, NantaoSzymborski, TomaszHu, QiongboSzymczyk, AnnaHu, WeiguoT. Hahn, NathanHu, YangTabari, TaymazHu, YiTabil, Lope G.Hu, YueTabish, Tanveer AhmadHu, YufangTabrizi, Mahmoud AmouzadehHu, ZhuofengTaccola, SilviaHuang, CaoxingTada, RuiHuang, ChangjinTada, ShigeruHuang, Chao-MingTaddei, FabioHuang, Cheng-LiangTadeusz, WosinskiHuang, ChiayiTadic, Nenad B.Huang, Chun-YingTadyszak, KrzysztofHuang, Chun-YuanTaffa, Dereje HailuHuang, DaliTagami, TatsuakiHuang, GangliangTaghinejad, HosseinHuang, GuoshengTaghinejad, MohammadHuang, Haw-MingTaghizadeh, AliHuang, Hsin-HuiTaghizadeh, MohsenHuang, HuiTagliacozzo, ArturoHuang, JinliangTaguet, AurélieHuang, Jung-JieTaha, JoseHuang, KeTahara, YoshiroHuang, LeiTaha-Tijerina, JaimeHuang, LunjieTaha-Tijerina, José JaimeHuang, MengTak, EunyoungHuang, PeipeiTakacs-Novak, KrisztinaHuang, PengTakahashi, MasatoshiHuang, SonglingTakahashi, YukikoHuang, TaizhongTakano, YuHuang, Tzu-YenTakashi, YanagisawaHuang, WeichunTakaya, TomohisaHuang, Wen ChangTakei, HiroyukiHuang, XianheTakemoto, ShinjiHuang, XiaohuaTakemura, KenshinHuang, XiaojunTakemura, YasushiHuang, XuriTakeuchi, TomonariHuang, YanyuTakoudis, Christos G.Huang, YaxinTakshi, ArashHuang, YongchaoTaladriz-Blanco, PatriciaHuang, YuTaleb, AbdelhafedHuang, Yuan MingTalneau, AnneHuang, ZengguangŢălu, ŞtefanHuang, ZhanfengTamanoi, FuyuhikoHuang, ZhengweiTamboli, MohaseenHuang, ZhiyuanTameev, AlexeyHuang, ZhulinTammana, Sree Rama ChandraHuc, VincentTammaro, LoredanaHueso, Jose L.Tampa, MirceaHüger, ErwinTamus, Zoltán ÁdámHughes, Robert A.Tan, Bee KangHuguet, Silvia SoriaTan, ChihshanHuh, ChunTan, JunjunHuh, Do SungTan, LinHuignard, Jean-PierreTan, ShaunHumayun, MuhammadTan, Wai KianHumbert, ChristopheTan, XiaopingHumelnicu, DoinaTan, ZhanaoHumelnicu, IonelTanabe, KatsuakiHuminic, AngelTanaka, YoshiakiHuminic, GabrielaTanaka, YuyaHung, Nguyen TuanTandecka, KatarzynaHung, Sung-FuTaneera, JalalHung, Tai-FengTang, BaokunHung, Yung-TseTang, ChaojunHunge, Yuvaraj M.Tang, ChristinaHunge, Yuvraj M.Tang, ChunHunter, Wayne BrianTang, LiangguangHuntosova, VeronikaTang, LihongHuo, QunTang, LinHuppertz, ThomTang, Ren-ChengHur, JaeTang, ShiHur, JaehyunTang, ShoufengHusen, AzamalTang, ShunqingHussain Ibhupoto, ZafarTang, XiaoningHussain, AqeelTang, XiuzhiHussain, FidaTang, YongjianHussain, ManwarTaniguchi, JunHussain, ShahidTao, PengHussain, Syed ModassirTao, WeiHussainova, IrinaTao, YunwenHussein, MahmoudTarabella, GiuseppeHutter, TanyaTaralli, EmanueleHuynh, Thanh-CanhTarasov, AlexeyHwang, Hyeon JunTarasov, MichaelHwu, Jenn-GwoTashima, DaisukeHytönen, VesaTashiro, ShoheiHyun Hur, SeungTasis, DimitriosIaccino, EnricoTasolamprou, AnnaIacob, Bogdan-CezarTassaddiq, AsifaIannazzo, DanielaTassinari, RobertaIanoul, A.Tatarchuk, TetianaIasiello, MarcelloTateno, HiroyukiIatridi, ZacharoulaTaufik, ArdiansyahIatsunskyi, IgorTaurino, Antonietta M.Ibarra, AlfonsoTavares, Jason RobertIbrahim, AbdelhameedTavares, PedroIbrahim, IslamTawfik, Wael Z.Ichikawa, MasakazuTaya, Sofyan A.Ichikawa, YoTayebi, MeysamIchimura, MasayaTayebi, TaharIgglessi-Markopoulou, OlgaTaylor, CianIglesias, Guillermo RTcherdyntsev, Victor V.Iglesias, OscarTealdi, CristinaIglesias-Juez, AnaTebyetekerwa, MikeIglesias-Martínez, EmiliaTeghil, RobertoIglic, AlesTeixeira, João PauloIgnat, MariaTeixeira, PilarIgnjatovic, NenadTeixidor, FrancescIhlemann, JürgenTelegdi, JuditIida, DaisukeTemleitner, LászlóIimori, ToshifumiTena, AlbertoIimura, SoshiTenchov, BorisIkegami, MasashiTeng, BotaoIkemoto, YukaTeng, Qian YongIkuno, TakashiTeodorescu, FlorinaIlahi, BouraouiTerakawa, MitsuhiroIlanchezhiyan, PugazhendiTeramoto, NaozumiIlchibaeva, TatianaTercjak, AgnieszkaIlg, PatrickTereshchenko, OlegIlia, GheorgheTermine, RobertoIlias, MiroslavTerraza, Claudio A.Iliescu, CiprianTertis, MihaelaIl’ina, Marina V.Testino, AndreaIliopoulos, IliasTeterycz, HelenaIlkhani, HodaThakre, AtulIlnicka, AnnaThakur, Vijay KumarIlyas, R.A.Thalhammer, GregorIm, Hyeon-GyunThamaraiselvan, ChidambaramIm, HyunsikThamburaja, PrakashImam, Syed SarimThang, Tran QuyetImani, JafargholiThangavel, GurunathanImasato, KazukiThangjam, Ibomcha SinghImperatore, RobertaThavasi, VelmuruganImpéror, MarianneThebo, Akbar AliIn, Su-ilTheerthagiri, JayaramanInada, RyojiTheodorakis, PanagiotisInbaraj, Baskaran StephenThersleff, ThomasInce, Ikbal AgahThiebault, ThomasInchingolo, FrancescoThirumal, VediyappanInclán, MarioThirumurugan, ArunInguanta, RosalindaThiruvengadam, MuthuInoue, JunichiThomas, NoelInui, NorioThomas, OlivierIoannides, TheophilosThomas, RenjithIoannis, K. KarabagiasThomas, SibyIoannis, KonidakisThompson, MichaelIoannis, SarrisThompson, Richard L.Ioele, GiuseppinaThorogood, Gordon JamesIoelovich, Michael JacobThrinath, Reddy RamireddyIon, Rodica-MarianaTian, BingbingIon, ValentinTian, FurongIonescu, Elena RodicaTian, Gui YunIoni, Yu.V.Tian, MingliangIonita, DanielaTian, MingweiIonita, PetreTian, ShouqinIonut, Ioana A.Tian, ShuangshuangIordache, FlorinTian, YanqingIordanova, EkaterinaTian, YeIordanskii, Alexey L.Tian, YuanIorio, Carlo SaverioTian, YuanxinIovieno, MicheleTibbetts, Katharine MooreIppili, SwathiTiberto, PaolaIqbal, JavedTie-Gang, WangIqbal, M.ZubairTiemann, MichaelIqbal, MudassirTien, Ching-HoIqbal, MunawarTien, Chuen-LinIqbal, ShahidTiggelaar, Roald M.Iqbal, ShahzadTihkonov, VladimirIqbal, TahirTimrov, IuriiIronside, Charles N.Tiribilli, BrunoIrusta, SilviaTirillo, JacopoIsaenko, L.I.Tiseanu, CarmenIsaeva, Vera I.Tishkevich, DaryaIsaiev, Mykola V.Titov, Aleksei A.Ishak, AnuarTittonen, IlkkaIshida, NobuhiroTivadar, LohnerIshida, TamaoTiwari, Arjun PrasadIshihara, OsamuTiwari, SantoshIshii, HaruyukiTjong, Sie ChinIslam, MohammadTkach, OleksandrIsmail, MuhammadTkalec, MirtaIsopescu, RalucaTobaldi, David MariaIssa, Shams A.M.Többens, DanielIstomin, Sergey YaTodd, Kathryn G.István, HornyákTodorova, SilviaItaliani, PaolaTodorović-Marković, BiljanaItina, Tatiana E.Tofan, LaviniaIto, AkitakaToffoli, DanieleIto, SeigoTokatly, IlyaIvakin, Yu.D.Toko, KiyoshiIvanauskas, RemigijusTokoroyama, TakayukiIvanov, MaximTokumoto, YukiIvanov, VictorTokunaga, EijiIvanov, Victor GenchevTokura, YasuhiroIvanov, Vladimir K.Toliński, TomaszIvanova, BojidarkaToloman, DanaIvanova, MariyaTolstopjatova, Elena G.Ivanova, T.Tomar, VartikaIvask, AngelaTomasic, VesnaIwami, KentaroTomasiunas, RolandasIwamoto, MitsumasaTomašovičová, NatáliaIwan, AgnieszkaTomasulo, StephanieIwanaga, MasanobuTomaszewicz, Elz̀bietaIwata, YukoTomaszewska, EmiliaIzadifar, MohammadrezaTombácz, E.Izyumskaya, NataliaTomčík, PeterJaan, AarikTomer, Vijay K.Jabbour, GhassanTomita, KojiJaber, José RaduanTomm, JensJacak, JanuszTommasi, Immacolata ConcettaJacak, LucjanTomšič, BrigitaJachimska, BarbaraTomsik, ElenaJackowski, KarolTondi, GianlucaJackson, DavidTong, DongshenJackson, Mark JamesTong, XinJadczak, Joanna N.Tong, YueyuJadoun, SapanaTong, ZhengrongJadwicienczak, WojciechTonti, DinoJaegers, Nicholas R.Tonucci, LuciaJafar, MakramTopchiy, MaximJaffrezic, NicoleTopoglidis, EmmanuelJagadeesh Kumar, AlagarasanToprak, Muhammet S.Jagminas, ArunasTopuz, FuatJahns, JuergenTorati, Sri RamuluJain, ShubhendraTorii, ShuichiJakhmola, AnshumanTornabene, FrancescoJakka, Suresh KumarToro, RobertaJakovenko, JiříTorokhtii, KostiantynJakubik, Wieslaw PawelToropov, NikitaJakubowski, WitoldTorrens-Serra, JoanJaleh, BabakTorrent-Burgues, JuanJałochowski, MieczysławTorres, IvanJamon, DamienTorres-Giner, SergioJampani, VSRTorres-Mendieta, R.Jamting, AsaTorres-Torres, CarlosJan, Jeng-ShiungTorsello, DanieleJana, AtanuTorsten, GranzowJana, JayasmitaTortolini, CristinaJanczarek, MarcinTotan, Alexandra RipszkyJanda, PavelTóth, ÁgotaJang, DaeIkToth, Andras JozsefJang, HoukTóth, GergőJang, JaewonToth-Katona, TiborJang, SoohwanTotu, EugeniaJanghorban, MaziarTouraud, DidierJankauskaite, VirginijaTräbert, ElmarJanner, DavideTrafny, ElżbietaJanovák, LászlóTraini, ToninoJanowska, IzabelaTrammell, Scott A.Jansto, Steven G.Tran, Anh VyJäntschi, LorentzTran, Ngoc QuyenJanus, WladyslawTran, Quang HungJapip, SusiloTran, Quang TrungJarząbek, BożenaTran, Tuan SangJarzabek, DariuszTrapani, MariachiaraJarzebski, MaciejTrenczek-Zajac, AnitaJasinski, RadomirTrens, PhilippeJáuregui-Vázquez, DanielTretiakov, KonstantinJavadian, HamedrezaTribelsky, MichaelJavaid, RahatTrifiletti, VaniraJaved, Muhammad SufyanTrindle, CarlJaworski, SławomirTringe, JosephJayaraman, TheerthagiriTrinh, ThuatJedrkiewicz, OttaviaTrinkler, LaimaJedrzejczak, AnnaTriolo, ClaudiaJeglic, PeterTrioni, Mario ItaloJeleń, PiotrTripathi, Kumud MalikaJelliss, PaulTripathi, NishantJelski, WojciechTripodi, AntonioJena, Himanshu SekharTriscone, Jean MarcJenkins, DavidTrník, AntonJenkins, Samir V.Trocino, StefanoJenny, Titus A.Trontelj, JanezJeon, HojeongTroyano, JavierJeon, JongwookTruccato, MarcoJeon, SangminTrucillo, PaoloJeon, Seog-JinTrujillano, RaquelJeon, SeokwooTrujillo-Cayado, Luis AlfonsoJeon, YukwonTrukhanov, Alex V.Jeong, Goo-HwanTrukhanova, Ekaterina L.Jeong, JeeyoonTrumble, John T.Jeong, Keun-YeongTruong, Vi-KhanhJeong, Seok-HwanTrusek, AnnaJeong, SunhoTrusek-Holownia, AnnaJeong, Young IlTrusov, Lev AJeong, Young-HunTrusso, SebastianoJergel, MatejTrybuła, ZbigniewJesionek, MarcinTryk, DonaldJesionowski, TeofilTrykowski, GrzegorzJha, RajveerTryznowski, MariuszJhon, Young-MinTsadilas, ChristosJi, JunhuiTsai, Cheng-MuJi, ShuxinTsai, Shiao-WenJi, WeiTsai, Tsung-HengJi, YongqiangTsakiridis, Petros E.Jia, JinTsakiroglou, ChristosJia, JinfengTsakmakidis, Kosmas L.Jia, XiaodongTsao, Chung ChenJia, XudongTsao, Hoi NokJian, ShunyiTsarev, AndreiJian, XianTsatsulnikov, AndreiJiang, Bang-PingTsay, CHien-YieJiang, ChengminTscheliessing, RupertJiang, DeliTschulik, KristinaJiang, GuoTseluikin, VitalyJiang, Hai-taoTseng, Wei-TauJiang, HaoTserkezis, ChristosJiang, HongchuanTsiakaras, PanagiotisJiang, LongboTsilipakos, OdysseasJiang, QingTsirigotis-Maniecka, MartaJiang, ShaTsivileva, OlgaJiang, ShaohuaTsivintzelis, IoannisJiang, WuguiTso, EdwinJiang, XiaoningTsogkas, GeorgeJiang, XuchuanTsou, Chi-HuiJiang, YongTsouknidas, AlexanderJiang, YueTsubaki, KazunoriJiang, YulongTsubaki, ShuntaroJiang, YuyanTsuji, HidetoJiang, ZhangTsuji, YasuhideJiao, TifengTsuji, YutaJicsinszky, LászlóTsuno, TakashiJijie, RoxanaTsutsui, MakusuJilani, AsimTsvetkov, NikolaiJiménez Blanco, José L.Tsyganenko, Alexey A.Jiménez, JuanTsyntsarski, Boyko G.Jiménez-Lamana, JavierTu, YonggangJiménez-Martí, ElenaTucci, VincenzoJiménez-Pérez, Jose LuisTuccitto, NunzioJin, Congrui GraceTucki, KarolJin, HellenTufa, Ramato AshuJin, HuaTulyaganov, DilshatJin, HuanyuTumarkin, AndreyJin, JunchengTunc, CemilJin, MingliangTunesi, MartaJin, SilaTung, Nguyen ThanhJin, YifeiTuranek, JaroslavJin, YingxueTurchenko, VitaliiJin, YoungeupTurco, RosaJin, ZhichengTurczyn, RomanJindal, V.K.Turkyilmazoglu, MustafaJinesh, Kochupurackal B.Turos, EdwardJing, DaleiTurovskaya, Maria V.Jing, LiqiangTuróvsky, Egor A.Jmerik, ValentinTuttle, BlairJnawali, GirirajTutuncuoglu, GozdeJo, Jae-HyeonTüzün, BurakJogaiah, SudishaTverdokhlebov, SergeiJohannes, Hans-HermannTyliszczak, BożenaJohansson, ChristerTylus, WłodzimierzJohari, Seyed AliTzeli, DemeterJohn, Rohit AbrahamTziveleka, Leto-AikateriniJohnson, EricTzoneva, RumianaJohnson, Matthew StanleyUbaidullah, MohdJohnsson, MatsUchacz, TomaszJohnston, Linda J.Uchaikin, SergeyJokar, EfatUchiyama, SusumuJoly, NicolasUdalov, OlegJones, MarjorieUddin, Md. NizamJönsson-Niedziolka, MartinUddin, Mohammad AfsarJordi-Roger, Riba RuizUddin, Mohammed JashimJose, Sanchez Del Rio SaezUddin, Prof. JamalJoshi, Hem ChandraUen, Wu-YihJoshi, NiravUeno, KoheiJoshi-Imre, AlexandraUesugi, KenjiroJouffret, LaurentUgarova, NataliaJouiad, MustaphaUjihara, MasakiJovanovic, JelenaUjjain, Sanjeev KumarJovanovski, VaskoUkrainczyk, NevenJózefczak, ArkadiuszUllah Khan, SamiJu, HeongkyuUllah, AzeemJucius, DaliusUllah, HabibJudzentiene, AstaUllah, Malik ZakaJue, Melinda L.Ullah, NabiJuhász, ÁdámUllah, SanaJulien, Christian M.Ulloa, Sergio E.Jun, Sang BeomUllrich, BrunoJung, DaehwanUllrich, CarstenJung, Gang SeobUm, Doo-SeungJung, In HwaUm, Han-DonJung, JaehanUm, In-WoongJung, Kyeong YoulUmair Ali, MuhammadJung, Kyung-HyeUmapathi, ReddicherlaJung, NamgeeUmar, KhalidJung, SeunhoUmer, MuhammadJung, SokheeUn, Ieng WaiJung, Yong ChaeUnfried, KlausJungnickel, ChristianUngor, DittaJuodkazis, SauliusUnterreiner, Andreas-NeilJuodkazytė, JurgaUrakawa, OsamuJuranić, Pavle N.Urban, FrancescaJurasekova, ZuzanaUrbanczyk-Lipkowska, ZofiaJuskenas, RemigijusUrbanek, PavelK.Mishra, RajneeshUrbina, AntonioKabadi, ShrutiUrsaki, VeaceslavKabanov, ViktorUshida, AkiomiKabel, AhmedUtgikar, VivekKabutey, AbrahamUthappa, Uluvangada ThammaiahKačiulis, SauliusUtochnikova, ValentinaKadam, AvinashUtsumi, ShigenoriKaddoumi, AmalUykur, EceKado, YuyaV. Povolotskiy, AlexeyKadokawa, Jun-ichiVacca, AnnalisaKador, LotharVacková, TaťanaKadyrzhanov, KayratVadahanambi, SridharKaganer, Vladimir M.Vadrucci, MoniaKaichev, V.V.Vafaei, SaeidKailasa, Suresh KumarVagin, MikhailKaiping, TaiVajeeston, PonniahKajetanowicz, AnnaVakros, JohnKakaraparthi, KranthirajaVaks, VladimirKako, TetsuyaValachová, KatarínaKalanur, Shankara SharanappaValagiannopoulos, ConstantinosKalbáčová, Marie HubálekValdés-Ramírez, GabrielaKalinowski, SlawomirValdez-Nava, ZarelKalish, AndreyValente, Artur J.M.Kalkan, A.KaanValentini, FedericaKalmár, JózsefValérie, MaravalKalogianni, DespinaValerini, DanieleKalozoumis, PanayotisValero-Romero, María JoséKaluderovic, GoranValicherla, Guru RaghavendraKaluža, LuděkValiev, DamirKalvachev, YuriValkai, SándorKamali, Ali RezaVallée, ChristopheKamali, SaeedVallejos, SaúlKamanina, NataliaValli, LudovicoKamaszewski, MaciejValoti, MassimoKameda, HidetoVamanu, EmanuelKamedulski, PiotrVan Der Heggen, DavidKamel, SamirVan Hoecke, KarenKameneva, SvetlanaVan Horn, Ryan M.Kameyama, TatsuyaVan Loef, Edgar V.D.Kaminski, Daniel M.Van Nostrum, Cornelus F.Kaminski, KamilVan Renterghem, WouterKamiński, MarcinVan Schepdael, AnnKaminski, PiotrVan Staden, RalucaKaminsky, WernerVan Thor, Jasper J.Kamışlıoğlu, MiraçVan Zalinge, HarmKamyar, Shirvani MoghaddamVančik, HrvojKamyshny, AlexVanek, PremyslKamysz, ElżbietaVanetsev, AlexanderKandasamy, GaneshleninVannet, Bart VandeKanduc, MatejVannini, AndreaKandula, SyamVáradi, CsabaKaneti, Yusuf ValentinoVaradwaj, ArpitaKang, HongkiVaradwaj, Pradeep R.Kang, HyeonggonVarani, LucaKang, HyoVarga, GáborKang, JungwonVarga, MariánKang, Moon-sungVarga, ZoltanKang, Sung BumVarin, Robert A.Kang, Sung MinVarma, Rajender S.Kang, YihuaVarna-Pannerec, MarianaKang, Young SooVarzi, AlbertoKang, YoungsooVaseashta, AshokKang, ZhenyeVashurin, ArturKangas, HeliVasi, SebastianoKannan, KarthikVasić, BorislavKanoun, Mohammed BenaliVasil’Eva, M.S.Kanoun, OlfaVasile, Bogdan StefanKantarelou, VasilikiVasilescu, AlinaKantartzis, Nikolaos V.Vasilevskaya, ValentinaKao, Ming-ChengVasilevskiy, Mikhail I.Kapcia, Konrad JerzyVasiliadis, EliasKaplan, AndreyVasu, DhananjayanKaptay, GeorgeVatsadze, SergeyKaradjova, IrinaVattikuti, S.V.PrabhakarKarahan, MehmetVattikuti, Surya Veerendra PrabhakarKarakashev, Stoyan IvanovVaz, FilipeKarakasidis, TheodorosVaz, Pedro D.Karamanis, PanaghiotisVazquez, ManuelKarami, BehrouzVazquez-Moreno, LuzKaran, SumantaVedhanarayanan, BalaramanKaranikolas, VasiliosVedyagin, Aleksey A.Karapantsios, ThodorisVeeramani, VediyappanKarapetrov, GoranVeeramuthu, LoganathanKarashanova, DanielaVeeraraghavan, VenkateshKaratrantos, ArgyriosVeiga-Suárez, FernandoKarbovnyk, IvanVekinis, GeorgeKarbownik, IwonaVekinis, GeorgiosKarczewski, GrzegorzVelasco, Aitor V.Kärenlampi, Petri P.Velasco-Lozano, SusanaKargar, FariborzVelazquez-Perez, Jesus E.Kargarzadeh, HaniehVelea, AlinKarimi, DanialVeleva, LucienKarimi-Maleh, HassanVelimirovic, MilicaKarimipour, ArashVelluto, DianaKarimov, DenisVelmurugan, SethupathiKarimullin, KamilVelmuzhov, AlexanderKariuki, BensonVelonia, KellyKarlina, A.I.Venditti, IoleKarlsson, HannaVenediktov, VladimirKärnfelt, CamillaVener, Mikhail V.Károly, ZoltánVeniaminov, AndreyKarpicz, RenataVenkateshwarlu, KattaKarpov, SergeyVenkateswaran, VivekananthanKarpushkin, EvgenyVennemann, AntjeKarushev, MikhailVercelli, BarbaraKasala, Prabhakar ReddyVerchenko, ValeriyKashapov, Ruslan R.Vercher Pérez, Rosa FranciscaKashif, MuhammadVereda Alonso, Elisa IsabelKasperski, AndrzejVereschaka, AlexeyKassab, Luciana Reyes PiresVereshchagin, AnatolyKassavetis, SpyrosVereshchagin, Anatoly N.Kasten, Benjamin B.Vereshchagin, SergeyKatančić, ZvonimirVereshchaka, Alexey A.Kataria, MonikaVergara, AlessandroKatayama, MasaoVergaro, VivianaKatelnikovas, ArturasVergaz, RicardoKaterski, AtanasVerheijen, Marcel AKatin, KonstantinVerho, OscarKatin, Konstantin P.Verna, AlessioKato, ShinyaVernardou, DimitraKatryniok, BenjaminVeronesi, StefanoKatsouras, AthanasiosVerros, George D.Katz, Jonathan I.Versaci, DanieleKaun, Chao-ChengVerucchi, RobertoKaushik, AjeetVeselova, AnnaKautek, WolfgangVetcher, AlexandreKavan, LadislavVia, BrianKavaz, DoğaViana Neto, BartolomeuKawaguchi, YukiViappiani, CristianoKawakita, HidetakaVicente, Miguel A.Kawamura, GoVictor J., RicoKawawaki, TokuhisaVidakis, NectariosKayumov, AiratVidmar, JanjaKazachenko, AleksandrVidu, RuxandraKazachenko, AlexandrViegas, CarlosKazakevicius, EdvardasViela, FelipeKazanskiy, NikolayVigdorowitsch, MichaelKazantseva, Nataliya V.Vijaya Anand, Mariadoss ArokiaKazimierski, PawełVijayakumar, SekarKazuma, HigashisakaVijayalakshmi, U.Keawsawasvong, SuraparbVijayaraghavan, VenkateshKeçili, RüstemVikraman, DhanasekaranKee, SeyoungVikrant, KumarKei Lung, Christie YingVikulina, AnnaKelarakis, AntoniosVikulova, EvgeniiaKelemen, MichalVikulova, MariaKeller, ArturoVilà, AnnaKenanakis, GeorgeVilanova, XavierKenawy, SayedVilardi, GiorgioKennedy, David C.Vilas-Boas, VâniaKenry, K.Vileno, BertrandKepinska, MartaVilhena, J.G.Kermene, VincentVilla, ElenaKern, FrankVilla, IreneKersten, GideonVillacampa Naverac, María BelénKeshavarz, MeysamVillasante, AranzazuKessler, Vadim G.Villeneuve-Faure, ChristinaKetoja, JukkaVinardell, Maria PilarKeyvaninia, ShahramVinayagam, RamachandranKhabashesku, Valery N.Vinnik, DenisKhachatryan, KarenVinogradov, Alexander S.Khade, RahulVioleta, PurcarKhadka, Dhruba B.Vipulanandan, CumaraswamyKhadtare, ShubhangiVirgili, TersillaKhaitouni, MohamedVirte, MartinKhajotia, Sharukh SVisa, AureliaKhajuria, Deepak KumarVisconti, PaoloKhalil, Ahmed S.GVishnyakov, Aleksey M.Khalil, AshrafViskadourakis, ZachariasKhalilov, LeonardVitale, StefaniaKhan, Abbas Z.Vitkalov, S.A.Khan, Abdul SamadVitorino, RuiKhan, AfrasayabVitrik, OlegKhan, Aftab Aslam ParwazVizureanu PetricaKhan, AfzalVlachos, DimitriosKhan, AjmalVlachos, NicholasKhan, ArifVlad-Bubulac, TachitaKhan, AslamVlaicu, Ioana DorinaKhan, FirozVlase, SorinKhan, IlyasVlcek, JaroslavKhan, Imran MahmoodVogelsang, JanKhan, M.IjazVohlídal, JiříKhan, MerajuddinVokoun, DavidKhan, Mohammad EhtishamVoliani, ValerioKhan, MuhammadVollbrecht, JoachimKhan, Muhammad FarooqVøllestad, EinarKhan, Muhammad KashifVolobujeva, OlgaKhan, MujeebVolodin, Alexander M.Khan, Sadaf BashirVolodin, VladimirKhan, Salah Ud-DinVolodkin, DmitryKhan, Sayed AliVolodymyr, TkachenkoKhan, SovannVon Freymann, GeorgKhan, UsmanVorberger, JanKhan, ZiauddingVoronenkov, VladislavKharaghani, DavoodVorotnikov, Yuri A.Kharissov, Boris IldusovichVoshavar, ChandrashekharKharlamova, Marianna V.Voshkin, AndreyKharlamova, Tamara S.Vostrikova, Kira E.Khatri, MuzamilVoué, MichelKhattab, TawfikVouvoudi, EvangeliaKhatua, Dipak KumarVoz, CristobalKhaydukov, YuryVrsalović-Presečki, AnaKhemthong, PongtanawatVuluga, ZinaKhezri, BaharehVygranenko, YuriKhlebnikov, Andrei I.Vyvenko, OlegKhlebtsov, Boris N.Wada, TakahiroKhodaparast, GitiWada, ToruKhodov, IlyaWadati, HirokiKhomich, AlexWagata, HajimeKhondakar, Kamil RezaWågberg, ThomasKhonina, SvetlanaWager, JohnKhonina, Svetlana N.Wagner, Jakob BirkedalKhorasani, Amir MahyarWagner-Wysiecka, EwaKhosravani, Mohammad RezaWahab, EssamKhudyakov, Igor VWahab, Mohammad AbdulKhusid, BorisWahab, RizwanKia, RezaWainstein, DmitryKidalov, SergeyWal, R.L.VanderKieninger, MartinaWałajtys-Rode, ElżbietaKierzek, KrzysztofWałęsa-Chorab, MonikaKietzig, Anne-MarieWalker, GregKiiamov, AiratWalkowiak, BogdanKiisk, ValterWalter, ThomasKijenska, EwaWalther, ThomasKijewska, MonikaWan, ChangjinKikugawa, NaokiWan, ChaoKilic, Mehmet EminWan, GangKim, Ae RhanWan, KaiKim, Baik-HoWan, YatingKim, Byoung SuhkWan, YiKim, Byung HyoWan, ZhiliKim, Chang-WooWandelt, KlausKim, Cheol GiWang, BenKim, ChoonsooWang, BenxinKim, DaeGwiWang, BinKim, DaehwanWang, BinghaoKim, DaewonWang, BoxiangKim, DaeyoungWang, ChanKim, DahyeWang, ChangdongKim, Dai SikWang, ChanghongKim, Dai-SikWang, ChenguoKim, Deok-keeWang, Cheng-YuKim, Dong YeongWang, ChenxiKim, Dong-HoWang, ChongwenKim, Dong-JooWang, ChongwuKim, Dong-ShikWang, Chun-KaiKim, Dong-WookWang, CongKim, DooryWang, CongweiKim, Hae-JinWang, DapengKim, Han JooWang, DaweiKim, Hee-JoonWang, DenanKim, HojunWang, DengxuKim, Hwa-JungWang, DongKim, HyeminWang, DongruiKim, Hyeong-UWang, ErkangKim, Hyun JaeWang, FajunKim, Hyun TakWang, FaxingKim, Hyun WooWang, FeiKim, HyunseokWang, FengKim, Hyun-SukWang, FengyunKim, IL TaeWang, FuKim, In SooWang, FukeKim, JeonghunWang, GuangzhaoKim, Jeong-HwanWang, GuigengKim, Ji-HeeWang, GuofengKim, JihoonWang, GuoqingKim, Jin SikWang, HaiboKim, JincheolWang, HaidongKim, Jung HoWang, HaiouKim, Jung YongWang, HaiyangKim, JungwonWang, HaoKim, Jun-HyunWang, HongsuKim, JuyoungWang, HuaKim, KeunsooWang, Hua PingKim, Kwang SWang, HuanKim, Kyong HonWang, HuiKim, Kyoung SubWang, JianKim, Kyoung-HoWang, JiangKim, KyoungtaeWang, Jian-GanKim, Kyoung-WhanWang, JiangtaoKim, Kyung-MinWang, JianjunKim, MinkookWang, Jian-WenKim, Minsoo P.Wang, JianyongKim, Moon DeockWang, JiaqiangKim, Moon IlWang, JinKim, MoonyongWang, JingKim, Myung-KiWang, JinguoKim, MyungwoongWang, JingweiKim, Na YoungWang, JinkeKim, Nam-HoonWang, JinlongKim, Patrick JoohyunWang, JinpingKim, Sang MoWang, JunKim, SangkilWang, KaiKim, SangmoWang, KaijunKim, SejungWang, KaiyingKim, Seong YunWang, KaiyouKim, ShinWang, KuaibingKim, SoaramWang, KunleiKim, Sung-HoonWang, LaiKim, SungjunWang, LeiKim, Sung-KonWang, LiangKim, SungminWang, LichangKim, Sun-JeWang, LifengKim, Tae-ilWang, LihuaKim, Won-KyungWang, LiqiangKim, Woo YoungWang, LiweiKim, Woo-HeeWang, LonggangKim, WookWang, LongluKim, Y.D.Wang, MingKim, YeonginWang, MingsongKim, Yong TaeWang, Ming-XiKim, YonghunWang, Mu-chunKim, Yong-SangWang, NannanKim, Yoong AhmWang, PengKim, Young-HoonWang, PengfeiKim, Young-KiWang, PingKim, YoungminWang, QiKimata, MasafumiWang, QingshengKimura, MasanariWang, QiuyiKimura, MutsumiWang, QizhaoKimura, YasuoWang, QunKing, GrahamWang, RenhengKioseoglou, GeorgeWang, RenxinKirchner, UlfWang, RidongKireev, DmitryWang, RuiKirihara, SoshuWang, ShanKirilenko, DemidWang, ShangfengKirk, AndrewWang, ShaokaiKirsch, Gilbert H.Wang, ShaoqingKirubasankar, BalakrishnanWang, ShenKiryukhantsev-Korneev, Ph.V.Wang, ShengKiselev, AntonWang, ShifaKish, LaszloWang, ShuaiKiskinova, MayaWang, ShuangpengKiss, AttilaWang, SisiKiss, JánosWang, SongcanKisslinger, RyanWang, WeiKistanov, AndreyWang, WeihongKita, HidekiWang, Wei-NingKitahama, YasutakaWang, WenxinKitta, MitsunoriWang, XiadongKlajnert-Maculewicz, BarbaraWang, XianwenKlapiszewski, ŁukaszWang, XiaodongKlauk, HagenWang, XiaofengKlavins, MārisWang, XiaojuKlébert, SzilviaWang, XiaoqianKleinke, HolgerWang, XinlongKlekotka, UrszulaWang, XiupengKlementieva, OxanaWang, XizuKlenk, ReinerWang, XudongKling, AndreasWang, Xue-LunKliros, George SWang, XushengKlontzas, E.Wang, YanKlos, Jaroslaw W.Wang, Yan-HsiungKlyamkin, Semen N.Wang, Yan-jieKmita, AngelikaWang, YanlingKnap, VaclavWang, YanqingKnápek, AlexandrWang, YanxiangKnerelman, Evgeniya I.Wang, YaoleiKnopf, GeorgeWang, YawenKnyazev, Yu.V.Wang, Yeong-HerKnyazev, Yuriy V.Wang, YiKo, Jong WanWang, YifeiKo, JunghyukWang, YinKo, SeunghwanWang, YingKo, Tsung-ShineWang, YinghuiKo, Young-HoWang, YongKobayashi, MotoyoshiWang, YouqingKobayashi, RikaWang, YuKobayashi, ShunsukeWang, YuanhaoKoblischka, MichaelWang, Yu-DeKoblischka, Michael R.Wang, YudiKobtsev, SergeyWang, Yun-CheKoch, MartinWang, YunmingKoch, UeliWang, YuxinKodali, MounikaWang, ZengboKoduru, JanardhanWang, ZepingKoduru, MallikarjunaWang, ZhenKoech, PhillipWang, ZhenxinKoel, MihkelWang, ZhifengKoepke, CzesławWang, ZhiqiangKogan, EugeneWang, ZhiyuanKoh, Yee RuiWang, ZhongruiKöhler, KlausWang, ZhuangKokkoris, GeorgeWang, ZiluKoksharova, Olga A.Wark, Alastair W.Kokubu, EitoyoWark, MichaelKokulnathan, ThangaveluWasiak, AndrzejKolar, MitjaWasserman, DanielKolaric, BrankoWatanabe, FumiyaKolasinski, Kurt W.Watanabe, MasashiKolencik, MarekWatarai, HitoshiKolishetti, NageshWatkins, Anthony NealKolmakov, Alexey G.Weatherley, Laurence R.Kolmas, JoannaWeber, BartKolmychek, Irina A.Weber, DanielKolodiazhnyi, Oleg IvanovWeber, LudgerKolotilov, Sergey V.Weber, MatthieuKolpakov, A.G.Weber, RobertKolsi, LiouaWegner, Karl DavidKołtunowicz, Tomasz NorbertWei, Da-HuaKolya, HaradhanWei, GangKomada, PawełWei, HuigeKomaguchi, KenjiWei, HuiyunKomanický, VladimírWei, JingKomarov, PavelWei, JingxuanKomarov, Pavel V.Wei, JunchaoKomogortsev, SergeiWei, LiKomolov, AlexeyWei, QiulongKomsa, Hannu-PekkaWei, WeiKondinski, AleksandarWei, XiangruKondo, TakahiroWei, XiankuiKondratyev, V.Wei, YuechangKonefaŁ, AdamWei, ZengxiKonev, AlexanderWei, ZhishunKong, HoyoulWeidlich, TomášKong, Kien VoonWeigand, RosaKong, Ling BingWeinberger, ChristianKong, LingbingWeinstein, Ilya A.Kong, WeiguangWeis, MartinKong, XiangdongWeisensee, PatriciaKong, XianmingWen, HuiminKongi, NadezdaWen, JianmingKönig, MarkusWen, LeiKonishi, KuniakiWen, ShihuiKonnova, SvetlanaWen, XinglinKononenko, Oleg V.Wen, YangpingKononevich, Yuriy N.Wen, YouhaiKonopatsky, Anton S.Wenderoth, MartinKonovalov, SergeyWhite, Jason C.Konstantaki, MariaWia̧cek, Agnieszka EwaKonstas, KristinaWiak, SlawomirKonteles, SpyrosWie, Jeong JaeKontonasaki, EleanaWieczorek, Władysław G.Kontos, Athanassios G.Wiedmann, SteffenKontziampasis, DimitriosWiekhorst, FrankKoo, Kevin M.Wierzbicka, EwaKoo, Kyo-inWiesława, MariaKoo, SangmoWiesner, MaciejKoodlur Sannegowda, LokeshWieszczycka, KarolinaKoos, Antal A.Wiglusz, Rafał J.Kopyl, SvitlanaWijayanta, Agung TriKordás, KrisztianWilkins, Richard TKordesch, MartinWilk-Kołodziejczyk, DorotaKoren, GadWilliams, Quinton L.Korepanov, VitalyWiltshire, Benjamin DanielKorobeynikov, S.M.Winiarski, Maciej J.Korolev, Dmitry S.Winkler, RobertKorolkov, Ilya VWinter, Jessica O.Korona, KrzysztofWiśniewski, KrzysztofKoroteev, Pavel S.Wisniewski, MarekKorshunov, Maxim M.Wisniewski, PiotrKörstgens, VolkerWissman, James P.Korzeniewska, EwaWitzel, BerndKorzhik, MikhailWłodarczyk, RenataKorzhikova-Vlakh, EvgeniaWojciechowski, SzymonKorzhikov-Vlakh, ViktorWojcieszak, RobertKosak, AljosaWójcik-Pszczoła, KatarzynaKosasih, Felix UtamaWojnar, PiotrKose, RyotaWojnarowicz, JacekKoseva, NeliWojnicki, MarekKosheleva, Ramonna I.Wojtkowska, MałgorzataKosinova, Marina L.Wojtyczka, RobertKosionis, SpyridonWojtyniak, MarcinKoskinen, PekkaWolarz, ErykKoslowski, ThorstenWolniak, RadosławKostevšek, NinaWolny, Juliusz A.Kostin, Gennadiy A.Wołowicz, AnnaKostopoulou, AthanasiaWołowiec, StanisławKostruba, AndriiWolski, LukaszKostyniuk, AndriiWong, Bryan M.Kotadiya, NareshWong, Chik HimKótai, LászlóWong, Roong JienKotarba, AndrzejWong, Shwin-ChungKotaro, MakinoWong, Siu HongKotek, JanWongchoosuk, ChatchawalKoter, StanislawWon-Yeop, RhoKothandaraman, JotheeswariWoo, Jong-ChangKotkowiak, MichałWoodfield, PeterKotlyar, VictorWoon, Wei-YenKotowska, UrszulaWorzakowska, MartaKotula-Balak, MałgorzataWróbel, PiotrKoubiti, MohammedWróbel, Rafał J.Kounatidis, IliasWrześniok, DorotaKoutavarapu, RavindranadhWu, BinKoutselas, IoannisWu, Chung-HsinKováč, JaroslavWu, ChunruiKovačević, BorislavWu, DiKovačević, DavorWu, FengKovacheva, DanielaWu, FugenKovačić, MarinWu, GuangfuKovacik, JaroslavWu, GuangleiKovalchuk, VolodjaWu, HaoKovalenko, SergeyWu, HuangKovalev, AnatoliyWu, J.M.Kovalev, Valeri I.Wu, JiaguiKovalev, ValeryWu, JianKovalevsky, Andrei V.Wu, Jiang-BinKovanis, VassiliosWu, JingKovaříček, PetrWu, JingjingKovrugin, Vadim M.Wu, JinmingKowalczyk, AgataWu, JunziKowalewska, AnnaWu, KaiKowalewska-Kudłaszyk, AnnaWu, LidongKowalik, PawełWu, LongKowalska, EwaWu, PinghuiKowalski, BogdanWu, QibaiKowalski, KazimierzWu, QingshengKowerdziej, RafalWu, Ren-JangKozak, MartinWu, ShangfeiKozhaev, MikhailWu, ShaohuaKozielski, Kristen LynnWu, TaoKozlov, GlebWu, WangsuoKozlov, Sergey M.Wu, WeiKozlova, Ekaterina A.Wu, WeiminKozlova, TaisiyaWu, Wei-TaoKozlovskiy, ArtemWu, WenjunKozma, GáborWu, XiangKrakowiak, StefanWu, XingKralj, SamoWu, YuKrálovec, KarelWu, Yung-ChunKramberger, ChristianWu, YupingKrapf, DiegoWu, ZhennanKrasikova, Raisa N.Wu, ZhibinKrasilin, AndreiWu, ZhongKrasnikov, AlekseiWunderlich, WilfriedKrasnikov, DmitryWyman, IanKrasnikov, Dmitry V.Wyrwas, BogdanKrasnoslobodtsev, Alexey V.Wysokinski, Karol IzydorKrasnov, PavelWyszkowski, MirosławKrasnov, Vladimir MXi, JunKrasovskii, EugeneXi, YonglanKrastev, RumenXia, ChangleiKrasteva, NataliaXia, GuangmeiKratochvilova, IrenaXia, MinggangKrauklis, AndreyXia, QiuyingKrause, Hans-JoachimXia, ShengjieKrause, MatthiasXia, ShengxuanKraut, ManfredXia, XinhuiKravchenko, Sergey V.Xia, YangjunKrawczyk, JoannaXiang, LanKrawczynska, Agnieszka TeresaXiang, PingKrawiec, MariuszXiang, QuanjunKrebsz, MelindaXiang, RongKrehula, StjepkoXiao, BingKremer, ReinhardXiao, BoqiKremieniewski, MarcinXiao, DengpanKrętowski, RafałXiao, MingKretschmer, SilvanXiao, MuKrichevtsov, BorisXiao, PengKriegel, IlkaXiao, ShuyuanKrishna, Mamidipudi GhanashyamXiao, TangxinKrishnamoorthy, RajavelXiao, Ting-HuiKrishnamoorthy, SivashankarXiao, WeiKrišťák, ĽubošXiao, XinKristóf, TamásXiao, YangKrisyuk, VladislavXie, ChenKrisztina, LászlóXie, HaifengKrivenkov, VictorXie, HongfengKrivetskiy, ValeriyXie, HuaqingKrivosudský, LukášXie, JijiaKrjuchkova, MarinaXie, JingKrólicka, AgnieszkaXie, JingyaKroll, PeterXie, KefengKrotkus, ArunasXie, LianwuKručaitė, GintarėXie, LinhuaKruczala, KrzysztofXie, MinghaoKruczała, KrzysztofXie, PeitaoKruczek, BoguslawXie, QingshuiKrüger, JörgXie, QinxingKrüger, PeterXie, RuosenKruit, PieterXie, ShaohuaKruk, AndrzejXie, TaipingKrumeich, FrankXie, YongjunKrupa, Kristen A.Xie, YuliangKrupinski, MichalXie, YunchaoKrylov, Alexander S.Xin, XiayingKrylov, IgorXing, BaolinKrystofiak, TomaszXing, FeiKrzywanski, JaroslawXing, GuichuanKrzywiecki, MaciejXing, LiliKrzyzanek, VladislavXing, ZhicaiKseniia, Baryshnikova V.Xiong, ChuanyinKshirsagar, AnurajXiong, FeiKubarev, VitalyXiong, HaoKubiak, Władysław W.Xiong, RanhuaKubiak-Ossowska, KarinaXiong, TianyingKucharska, EdytaXiong, ZhuoKüçüköz, BetülXiu, Fu-RongKudelski, AndrzejXiu, PengyuanKudryasheva, Nadezhda SXu, Ben B.Kudryashov, SergeyXu, BinKudryashov, Sergey I.Xu, ChunjianKudryashova, OlgaXu, GangKudryavtsev, KonstantinXu, HaipingKugel, K. I.Xu, HenghuiKuhn, RamonaXu, HengyiKuila, TapasXu, HongboKujawa, JoannaXu, HuKujawski, WojciechXu, HuapingKukla, ChristianXu, JianKukli, KaupoXu, JianxiongKukulka, David J.Xu, JiaqiangKulaots, IndrekXu, Jin-ShiKulinich, SergeiXu, JinyangKulova, TatianaXu, KaiKumagai, ShoheiXu, KaichenKumar R, NaveenXu, KaikaiKumar, AniketXu, LeileiKumar, ArunXu, LuKumar, Govindan SureshXu, PengchengKumar, ManishXu, QiKumar, ManojXu, QuanKumar, NaveenXu, RongguangKumar, PankajXu, RuijieKumar, PawanXu, SaiKumar, PraveenXu, ShoujunKumar, RajeshXu, ShuKumar, SanjeevXu, TaoKumar, SantoshXu, TianhuaKumar, SatyendraXu, TingtingKumar, ShailabhXu, WeiKumar, SudhirXu, WenzhanKumar, VanishXu, XiaojieKumar, Vijay BhooshanXu, XiaominKumar, VineetXu, XiaoxinKumar, VinodXu, XiaoxueKumar, Yedluri AnilXu, XijunKumaresan, YogeenthXu, XingtaoKümmell, TilmarXu, XinhuaKunal, PranawXu, XuhuiKung, Chung-WeiXu, Yan-BinKung, Yu-RueiXu, YangyangKunsagi-Mate, SandorXu, Yi-JunKuntsevich, Alexander YuXu, YongKunugi, TomoakiXu, ZhenboKunwar, SundarXu, ZhenglongKuo, Chi-ChingXu, ZhihengKuo, Chil-ChyuanXuan, ShouhuKuo, Po-ChihXuan, TongtongKuo, ShiaoweiXuan, XingKuo, Tsung-RongXue, BailiangKuo, Wen-ChengXue, MinKüpferling, MichaelaXue, XingjianKuppusamy, PalaniselvamXue, ZhaoguoKurajica, StanislavXue, ZhaohuiKurc, BeataXuehui, ZhangKurcok, PiotrXu-Monette, Zijun Y.Kurlyandskaya, Galina V.Yabuki, SoichiKurpaska, LukaszYabuuchi, NaoakiKusano, YoshihiroYacaman, Miguel JoseKusior, AnnaYadav, AnujaKuskov, AndreyYadav, HemrajKuśmierek, KrzysztofYaïci, WahibaKuss, ChristianYakimov, Andrew I.Kustov, Leonid M.Yakimov, Evgeniy B.Kuśtrowski, PiotrYakimova, LuidmilaKuthati, YaswanthYakoumis, IakovosKuyukina, Maria S.Yakunin, AlexanderKuzhir, PolinaYalikun, YaxiaerKuźma, ŁukaszYamada, ShigeyukiKuzmicheva, GalinaYamaguchi, HiroyasuKuzmin, AlexeiYamaguchi, MasafumiKuznetsov, AlekseyYamaguchi, MasahikoKuznetsov, DenisYamaguchi, SoichiroKuznetsov, SergeyYamamoto, GoKuznetsov, Vladimir L.Yamamoto, KaoruKuznetsova, Iren E.Yamamoto, MasanoriKuznetsova, LyubaYamamoto, YohKuznetsova, YuliaYamasaki, MasaoKuźniarska-Biernacka, IwonaYamato, TakehikoKuźnik, NikodemYamawaki-Ogata, AikaKuzyk, AntonYan, AidongKvítek, OndřejYan, BinKvon, Ze DonYan, DayunKweinor Tetteh, EmmanuelYan, DongpengKwon, Dong-JunYan, JiahaoKwon, KideokYan, JianhuaKwon, Ki-YoungYan, MingKwon, Oh JoongYan, ShanchengKwon, Oh SeokYan, ShuoKwon, Seong JungYan, XinlinKyaw, Aung Ko KoYan, YonghuaKyriakopoulos, Grigorios L.Yan, ZhenhuaKyriakou, GeorgeYan, ZhiKyzas, GeorgeYandouzi, MohammedLa Mantia, Francesco PaoloYáñez-Sedeño, PalomaLa Rubia, M.DoloresYang, BinLa Spada, LuigiYang, BingLa, Duc DuongYang, BoLachman-Senesh, NoaYang, BoboLadero, MiguelYang, CaoLaGrow, Alec P.Yang, ChanghongLai, Chin WeiYang, Chang-TongLai, JianchengYang, ChaoLai, Jui-YangYang, Cheng-FuLai, MinliangYang, ChenhuiLai, Ping-ShanYang, Chia-MinLai, SarahYang, Chi-YuanLai, Wei-ChihYang, DantingLai, Yi ShengYang, DapengLaity, Peter RYang, DongfangLak, AidinYang, DongliangLalbakhsh, AliYang, FanLaloy, JulieYang, GuangzeLamberti, FrancescoYang, HaiguiLambin, PhilippeYang, Hang-ShengLambrecht, WalterYang, HaoLamiel, CharmaineYang, HongjiuLampart, PiotrYang, HongtaLamperti, AlessioYang, HuLamponi, StefaniaYang, HuachaoLan, ChangyongYang, HyunikLan, LinfengYang, In-SangLan, Wen-HowYang, JianjunLančok, JánYang, JingxiuLanda-Medrano, ImanolYang, JunLanfredi, SilvaniaYang, KaiLang, Gyözö G.Yang, LeLanzi, MassimilianoYang, LijunLaoutid, FouadYang, LimingŁapiński, AndrzejYang, LingweiLapushkina, TatianaYang, LiniLarichev, YuriiYang, LiuLarin, SergeyYang, LixiaLarionov, Vladimir A.Yang, Min YangLarsson, HenrikYang, Min-QuanLaszlo, KotaiYang, PinganLászló, KrisztinaYang, Po-ChihLászló, ZsuzsannaYang, QingLatal, JanYang, ShengLathiotakis, Nektarios N.Yang, SongLatorrata, SaverioYang, TieLatouche, CamilleYang, TingtingLattuada, MarcoYang, XiaofeiLatychevskaia, TatianaYang, XiaoyuLäubli, Nino F.Yang, XinglinLaudadio, EmilianoYang, XinpingLaudyn, Urszula A.Yang, Xiu-LiLauto, AntonioYang, YanbingLavoine, NathalieYang, YangLavrinenko, AndreiYang, YongLaw, Adrian W.K.Yang, YongzhenLaw, StephanieYang, YouwenLázár, IstvánYang, YuminLázár, KárolyYang, ZaixingLazarević-Pašti, TamaraYang, ZhengchunLazarides, N.Yang, ZhengpengLazarova, HristinaYang, ZhiLazarus, NathanYang, ZhujinLazău, CarmenYanguas-Casás, NataliaLazzara, GiuseppeYanilmaz, MeltemLe Faou, Alain E.Yano, MasafumiLe Floch, SylvieYao, JiandongLe Gall, TonyYao, JianhuaLe Moyne, LuisYao, JiyongLe Ouay, BenjaminYao, ShanshanLe Thai, DuyYao, ShuhuaLe Tilly, VéroniqueYao, WeirongLe, Thanh-HaiYao, YaoLe, Thi ThuYao, ZhichengLe, Thien AnYao, ZhikanLeal, João PauloYaqoob, Asim AliLeal-Junior, ArnaldoYaraki, Mohammad TavakkoliLebedev, Alexander AlexandrovichYarmohammadi, MohsenLeBlanc, Roger M.Yaroslavtsev, AndreyLeca, VictorYaroslavtsev, Andrey B.Lecaros, Rumwald LeoYashchenok, AlexeyLeclère, PhilippeYasin, GhulamLedari, Reza TaheriYasin, SohailLee, Chan WooYassar, AbderrahimLee, Chang YoungYastrebov, SergeyLee, Chang-WonYasui, KyuichiLee, Chia-HungYasui, TakaoLee, Chih-HaoYasuzuka, SyumaLee, Chuan-PeiYazdanmehr, MaryamLee, Chung-SungYazdimamaghani, MostafaLee, DasolYe, Cai-ChaoLee, DongguYe, EnyiLee, DongkyuYe, HanLee, DoojinYe, Jing YongLee, Eun YoungYe, JunweiLee, Eun-CheolYe, LindaLee, EunhoYe, MeidanLee, Gang HoYe, QingLee, Geon JoonYe, Run-PingLee, GibaekYe, ShuaiLee, Gi-JaYe, TenglingLee, Han EolYe, XiaofengLee, Hee ChulYedluri, Anil KumarLee, Hiang KweeYeh, Chao-HuiLee, Hong-KiYeh, Chun-LiangLee, Hsien-MingYen, Yee-wenLee, IlkeunYenugu, Veera Manohara ReddyLee, InheeYeo, Min-KyeongLee, I-TaYeom, JunghoonLee, Jae SungYerushalmi, LalehLee, Jae-chulYerushalmi-Rosen, RachelLee, JaehanYeum, JeongLee, Jae-SeungYeung, Andy Wai KanLee, JaewookYi, JunLee, Jea UkYi, Dong KeeLee, JechanYi, HaiboLee, Jennifer D.Yi, YougenLee, Ji HoonYi, ZaoLee, Jin HeeYim, ChangyongLee, JinhoYin, ChaoLee, Jong-ChulYin, FengLee, Jong-KwonYin, HanLee, Jong-SookYin, HongfengLee, Ju-HanYin, HongranLee, Jung-JaeYin, JianLee, JunseopYin, KaiLee, KangtaekYin, LiangjunLee, Kee-SunYin, ZhenLee, Kun-MuYin, ZhigangLee, Kyoung G.Yiu, CynthiaLee, KyungminYokochi, AlexandreLee, Man-JongYokose, SatoshiLee, Mei-HwaYokota, ShingoLee, NaesungYong, Kar WeyLee, Sang BongYong, XueLee, Sang HunYoo, Dong JinLee, Seok JaeYoo, HocheonLee, Seung GooYoo, HyundeogLee, Seung HeeYoo, Ik-KeunLee, SeunghyunYoo, JeeyoungLee, Seung-JaeYoo, JinkyoungLee, Shyi-LongYoo, KeunjeLee, TaewooYoo, KicheonLee, UnggooYoo, Tae JinLee, Wook JinYoo, Yong KyoungLee, Y.S.Yoo, YoungdongLee, Ying-ChiehYoon, Dae-HoLee, Young WookYoon, Do YeungLee, YoungkwanYoon, Hyeun JoongLee, Yu-HsuanYoon, Hyo JaeLeem, Jung WooYoon, JungjinLegut, DominikYoon, MinyoungLehocký, MariánYoon, YeojoonLehr, Claus-MichaelYoshida, EriLehtonen, AriYoshida, KentaroLei, MingYoshida, YoshikazuLei, Xiao-WenYoshihara, ToshitadaLei, YanhuaYoshimatsu, KoheiLeinweber, PeterYou, BoLeitão, Jorge H.You, HongjunLeite, Edson RobertoYou, XiangchengLeitner, MichaelYoun, Duck-hyunLekakou, ConstantinaYounes, HammadLelidis, IoannisYoung, Howard A.Lelièvre, BénédicteYoung, Kyu JeongLelis, MartynasYounis, AdnanLelli, MarcoYousefi, RaminLemercier, GillesYousefinejad, SaeedLemordant, DanielYperman, JanLeng, YuxinYu, ChunleiLenzi, MoniaYu, DaquanLeo, AntonioYu, DeipingLeo, ElianaYu, Deng-GuangLeón, Judith Martín-DeYu, DongliangLéonard, EstelleYu, DuanLeonardi, AlbertoYu, FabiaoLeonat, LuciaYu, FangLeong, JiayuYu, FengLeong, Jik-ChangYu, HaifengLeonidas, PalilisYu, HuiminLeonov, AndreyYu, Ing-SongLeonowicz, ZbigniewYu, Jae-WoongLeonski, WiesławYu, JiayuanLepadatu, Ana-MariaYu, JingangŁepkowski, Sławomir P.Yu, LeLeporatti, StefanoYu, LixinLeprince-Wang, YaminYu, MiaorongLerer, Alexander M.Yu, PengLesiak-Orłowska, BeataYu, PingpingLesnyak, VladimirYu, QilinLesov, IvanYu, ShihuiLessard, BenoitYu, ShudongLeszczynski, MikeYu, TaekyungLettieri, MariateresaYu, XiaowenLettieri, StefanoYu, XuLeu, Ching-ChichYu, YangxinLeung, KenYu, Yeon-TaeLeung, KevinYu, YongshengLévêque, PatrickYu, YouhaiLevin, Aleksandr A.Yu, YunLevin, Oleg VladislavovichYu, ZebinLevine, Mindy S.Yu, ZuyuanLevy, MiguelYuan, BaiqingLewenkopf, CaioYuan, Cadmus AnnLewinska, AnnaYuan, ChangzhouLewis, David A.Yuan, Judy Chia-ChunLhuillier, EmmanuelYuan, MengweiLi Voti, RobertoYuan, ShaojunLi, AihuaYuan, YeLi, AngYuan, ZhangfuLi, BaichangYuan, ZhenLi, BinYuan, ZhiqinLi, Bing-ShengYue, PengfeiLi, BingxiYue, TongtaoLi, BingzhiYue, YinghongLi, ChengYuen, Jonathan D.Li, ChengpengYugami, HirooLi, ChenyangYulin, AlexeyLi, ChunhuYun, ChanghunLi, Cui-PingYun, Hyung JoongLi, DanYun, RuiruiLi, DepingYuryevich, Gerasimenko AlexanderLi, DongshengYusenko, Kirill VLi, DongxiangYusoff, Rashid Bin MohdLi, FeiYuvaraj M., HungeLi, FengZabeida, OlegLi, FenghuaZach, RyszardLi, FengmingZada, AmirLi, GangZadin, VeronikaLi, GuangliZaharescu, TraianLi, GuangyiZahariev, FedericoLi, GuangyuanZaidi, Shabi AbbasLi, GuanyingZairov, RustemLi, Gui-caiZaitsev, VladimirLi, GuodongZaitsev, Vladimir B.Li, GuoLingZaka Ullah, MalikLi, GuoqiangZakaly, Hesham MHLi, HaiZakaria, Mohamed B.Li, Hai BoZakaria, Mohamed BarakatLi, HanyangZakharov, Alex V.Li, HaoZakharova, LuciaLi, HebinZako, TamotsuLi, HongdaZakowski, KrzysztofLi, HongguangZakrzewska, KatarzynaLi, HongyanZalas, MaciejLi, HuZalden, PeterLi, HuanglongZalewski, PrzemysŁaw ł.Li, HuiZaltariov, Mirela-FernandaLi, HuiliZaman, MohammadLi, JiabaoZaman, ShahidLi, JianlinZambito, YleniaLi, JianzhaoZambon, DanielLi, JinZambrano, Maria De La LuzLi, JinganZamocky, MarcelLi, JingchaoŻamojć, KrzysztofLi, JingleiZamora-Ledezma, CamiloLi, JinhuaZampetti, EmilianoLi, JinliangZan, GuangtaoLi, JizhenZanacchi, Francesca CellaLi, JunZandvliet, HaroldLi, JunhuiZang, ZhigangLi, JunjieZangari, GiovanniLi, JunruiZangeneh-Nejad, FarzadLi, Jun-XiaZannier, ValentinaLi, KaitaoZannotti, MarcoLi, LanZanotto, SimoneLi, LiangZaragoza Contreras, Erasto ArmandoLi, LianzhiZarrabi, AliLi, LingweiZarrabi, Ferdows B.Li, LinnanZarzycki, ArkadiuszLi, MeichengZatsepin, Anatoly F.Li, MeichunZauscher, StefanLi, MengZavoianu, RodicaLi, MingxingZdanowicz, MagdalenaLi, Ming-YuZdorovets, MaximLi, MoZdunek, KrzysztofLi, PengZebrev, GennadyLi, PingZec, NebojšaLi, QiZegaoui, OmarLi, QiangZeilinger, CarstenLi, QianqianZeinert, AndreasLi, RenxianZekker, IvarLi, RuiZekry, AbdelhalimLi, RukangZelenovskiy, Pavel S.Li, ShandongZelepukin, Ivan V.Li, ShengquanZelkó, RománaLi, ShiqiZeng, BirongLi, SongsongZeng, GuangyongLi, TaoZeng, JianrongLi, TianlongZeng, KaifangLi, WanjunZeng, ShuwenLi, WanlongZeng, TaoLi, WeiZeng, XianLinLi, WeitaoZeng, YikaiLi, WenshengZeng, YujiaLi, Wen-TyngZeng, ZhihuiLi, XiaoganZeng, ZhipingLi, XiaohongZeng, ZiqianLi, XiaohuiZenin, Vladimir A.Li, XiaomingZerti, DarinLi, XiaopengZerulla, DominicLi, XiaoyanZghal, SouhirLi, XiaoyiZglobicka, IzabelaLi, Xing’aoZhai, HuifeiLi, XingyunZhai, LeiLi, XiuqiangZhai, ShumeiLi, XueyuZhai, TianruiLi, XuningZhan, ZaijiLi, YafengZhang, BaitaoLi, YaminZhang, Bao YueLi, Yan-BoZhang, BingsenLi, YangguangZhang, CailiLi, YiwenZhang, ChaocanLi, YongfengZhang, ChengLi, YonggangZhang, DaojunLi, YongjinZhang, DongLi, YuanzuoZhang, DongshiLi, YueZhang, DongyangLi, YuhaoZhang, Duan Z.Li, ZhelingZhang, ErlinLi, ZhengZhang, FanyongLi, ZhengqiangZhang, Feng-JunLi, ZhixiongZhang, FujunLi, ZhonghaoZhang, GuigangLi, ZhongshuiZhang, GuobinLi, ZhouZhang, GuofengLi, ZifuZhang, HaichangLi, ZiweiZhang, HaifengLi, ZiyuanZhang, HaijunLian, ChengZhang, HaoLian, XiaojuanZhang, HaochunLian, YongfuZhang, HongguangLiang, BoZhang, HongliangLiang, ChaofengZhang, HuangLiang, Chi-TeZhang, JianLiang, Fang-ChengZhang, JianbingLiang, GangZhang, JianyongLiang, JiZhang, JieLiang, JianwenZhang, JincanLiang, LijunZhang, JingdongLiang, RuijingZhang, JinxinLiang, RuowenZhang, JitaoLiang, Y.F.Zhang, JunLiang, YaoZhang, JunjieLiang, YongpingZhang, LanLiang, YongriZhang, LeiLiang, Yuan-ChangZhang, LiLiang, YucangZhang, LiangLianos, PanagiotisZhang, LingLiao, JiaxuanZhang, LuLiao, MeiyongZhang, MinfangLiao, Ming HanZhang, NaLiao, XiaozhouZhang, NingLiao, XinqinZhang, PengLiao, Ying-ChihZhang, PengboLibra, MartinZhang, QifengLi-Destri, GiovanniZhang, QingfengLieder, MarekZhang, RongfaLiedienov, NikitaZhang, RongjunLikodimos, VlassiosZhang, RufanLikozar, BlažZhang, SenLim, DaewoonZhang, ShuleLim, Dong ChanZhang, ShushengLim, EunhoZhang, SonglinLim, HyunseobZhang, TianyueLim, HyunsooZhang, TieruiLim, Jae-HanZhang, TingLim, Seong ChuZhang, TongLim, Yee-FunZhang, WanLima, MarioZhang, WeiLimbu, TejZhang, WeibingLin, Cheng-TeZhang, WeidongLin, Chia-HerZhang, WenLin, Chin-FengZhang, WengangLin, Chu-HsuanZhang, WenliLin, Chun-HoZhang, WenmingLin, Chun-RongZhang, XiangLin, DabinZhang, XiaodongLin, DaohuiZhang, XiaoshuanLin, Der-YuhZhang, XiaoyanLin, HaoshengZhang, XinghongLin, HsinchihZhang, XinpingLin, Hung-YinZhang, XiutangLin, Jia-HorngZhang, XiyueLin, JianzhongZhang, XuLin, JieZhang, XuanLin, JinghuangZhang, Xuan-junLin, Jui-CheZhang, XueaoLin, Kuang-IZhang, XuejinLin, LinhanZhang, XunLin, Meng-FangZhang, YaGangLin, MingZhang, YanfengLin, ShenghuangZhang, YanpengLin, Shuo ParkZhang, YapeiLin, SijieZhang, Ye-WangLin, WangZhang, YiLin, WeifengZhang, YifuLin, WenjingZhang, YijunLin, XiaochengZhang, YingLin, XiuyiZhang, YingjieLin, YangZhang, YingmingLin, Yang-weiZhang, YingyiLin, Ying-WuZhang, YiyunLin, YuankunZhang, YongkangLin, Yu-CheZhang, YongtaiLin, ZhaoyangZhang, YuLin, ZhiZhang, YuanhangLin, ZhibinZhang, YuanweiLinares, MaríaZhang, YueLinda, CattinZhang, YunLindner, ThomasZhang, YundongLing, JianZhang, YunyanLing, Yong-ChienZhang, ZaoliLing, YunZhang, ZekaiLingvay, IosifZhang, ZeminLinklater, DenverZhang, ZhangLinul, EmanoilZhang, ZhaofuLio, GiuseppeZhang, ZhenbinLionetto, FrancescaZhang, ZhengLiotta, LeonardaZhang, ZhenyiLipiński, MarekZhang, ZhidongLipiński, StanisławZhang, ZhijieLiras, MartaZhang, ZhiliLis Arias, Manuel J.Zhang, ZhiyongLis, Jose Vicente RosZhang, ZidanLisa, GabrielaZhang, Zi-YangLisi, NicolaZhang, ZongtaoLisowska-Oleksiak, AnnaZhao, BinList, ManuelaZhao, BingLittler, ChrisZhao, BoLitvin, Aleksandr P.Zhao, DaweiLitwinienko, GrzegorzZhao, DielingLiu, BaijunZhao, FeipingLiu, BaiquanZhao, GuixiaLiu, BinZhao, GuopingLiu, BiwuZhao, HengLiu, Bo-TauZhao, HongliLiu, BoyaZhao, HongyuanLiu, CailongZhao, JiangLiu, ChangkunZhao, JianlingLiu, ChaoZhao, JiminLiu, Chih-YiZhao, JingtaiLiu, ChongZhao, KaiLiu, ChongyangZhao, LeiLiu, ChuanZhao, MengqiangLiu, Chung-ChiunZhao, MingchunLiu, DaojunZhao, MinghaoLiu, DejunZhao, QianLiu, Der-ZenZhao, QinglanLiu, DongZhao, ShaoyuLiu, FangzeZhao, ShenlongLiu, FengZhao, ShuangyiLiu, FupinZhao, SikaiLiu, GuanlinZhao, TianmingLiu, GuichengZhao, Tian-ShengLiu, GuodongZhao, WeiLiu, GuokunZhao, WeiweiLiu, HaoZhao, XiaoningLiu, HongyuZhao, XuLiu, HuanZhao, YanLiu, Hua-pingZhao, YiLiu, HuiZhao, Yong-BiaoLiu, HuiliZhao, YudaLiu, JiZhao, YuzhouLiu, JianqiangZhao, ZhanyongLiu, Jian-WeiZhao, ZhenghuanLiu, JingZhao, ZongshanLiu, JingangZharkov, SergeyLiu, JingchengZharnikov, MichaelLiu, JinlongZhbanov, AlexanderLiu, JinmingZhdanov, Andrey P.Liu, JinyaoZheng, ChangchengLiu, JixingZheng, DafengLiu, JixuanZheng, DingLiu, JiyangZheng, GaigeLiu, JunZheng, GaofengLiu, Jun-JenZheng, GongLiu, JunjieZheng, GuoquLiu, JunqiuZheng, HongyuLiu, KaiZheng, KunLiu, KaikaiZheng, LuyaoLiu, LijunZheng, MengLiu, LizheZheng, QiyeLiu, ManingZheng, XintingLiu, MengZheng, YiqunLiu, PeijiangZheng, YudongLiu, QianZheng, YunLiu, QihanZhong, DonglaiLiu, QingchangZhong, WenwuLiu, RenbaoZhong, ZhaoxiangLiu, RuixiaZhou, ChaobiaoLiu, RuiyuanZhou, ChunLiu, Shi-YongZhou, GuoweiLiu, ShuangZhou, HaiboLiu, ShudeZhou, HaolongLiu, ShuliZhou, HongLiu, SiqiZhou, HongjianLiu, TaihongZhou, HongjunLiu, TaoZhou, JiangLiu, TianlongZhou, JianhuaLiu, TianqingZhou, JingyuanLiu, WeiZhou, JiongLiu, WeidiZhou, KunLiu, Wei-RenZhou, LiLiu, WeishuZhou, LongLiu, WeiweiZhou, MingjiongLiu, WenboZhou, QingLiu, WenjunZhou, QixingLiu, WumingZhou, RuLiu, XiangdongZhou, ShaoboLiu, XiangleiZhou, ShengjunLiu, XianjieZhou, TianhuaLiu, XiaoZhou, WenyingLiu, XiaobingZhou, XiangLiu, XinZhou, XiaoyuanLiu, XinhuaZhou, XuebingLiu, XinkeZhou, YeLiu, XinleiZhou, Ying-GuoLiu, XinmeiZhou, YiqunLiu, XinyuZhou, YongLiu, XiuhuaZhou, YoufuLiu, Xue-QingZhou, ZhangkaiLiu, YanZhou, ZhuxianLiu, YanghuiZhou, ZuowanLiu, YanjuZhu, BinLiu, YaoranZhu, ChengLiu, YaowenZhu, ChuhongLiu, YiZhu, ChunxiangLiu, YingZhu, HaoLiu, Ying DanZhu, HuaLiu, YingliangZhu, Hugh LuLiu, YingtaoZhu, HuiLiu, YingyaZhu, JiangongLiu, YipuZhu, JianxiongLiu, YongZhu, JinLiu, YongshengZhu, LeiLiu, YuZhu, LihuaLiu, YuchengZhu, LongfengLiu, YuelianZhu, MaiyongLiu, YuxiangZhu, MiaomiaoLiu, ZekeZhu, MinLiu, ZhaopingZhu, MingqiangLiu, ZhijunZhu, MinminLiu, ZhongyunZhu, QiLiu, ZhuangjianZhu, SidiLiu, ZihangZhu, SuiyiLiyanage, PiumiZhu, TingLjubas, DavorZhu, TongheLlena Puy, Maria Del CarmenZhu, WenliangLo Faro, Maria JosèZhu, XiaosongLo, Kuang YaoZhu, XuFeiLobato, JustoZhu, YifanLobato, KillianZhu, YonggangLocci, AntonioZhu, YuefengLocci, Antonio MarioZhu, YusongLoddo, VittorioZhuang, JianleLofland, SamZhuang, QixinLokteva, Ekaterina S.Zhuang, QuanchaoLomenzo, Patrick D.Zhuang, ZheLone, SaifullahZhuo, LongchaoLong, BeiZhuo, YueLong, Didier R.Ziani, Niccolò TraversoLong, GuankuiZielinski, AndrzejLong, LingliangZielinski, PiotrLong, Nicholas J.Ziemkowska, WandaLong, XuZier, TobiasLonghi, MariangelaZierkiewicz, WiktorLongo, AngelaZierold, RobertLongo, MassimoZille, AndreaLongo, PasqualeZimina, AnnaLongo, SandroZimmermann, IwanLopes, AndréZimnyakov, DmitryLopes, Carla MartinsZinatloo-Ajabshir, SaharLopes, CláudiaZinchenko, AnatolyLopes, LuiseZinicovscaia, IngaLópez Martínez, JuanZinin, Pavel V.López Salinas, José LuisZiółkowski, PawełLópez, ConcepciónZiora, Zyta MariaLópez, Miguel ÁngelZissis, GeorgesLopez, ReneZivieri, RobertoLopez-Garzon, RafaelZiyatdinova, GuzelLopez-Jornet, PiaŻołądek, SylwiaLopez-Linares, FranciscoZolali, AliLópez-López, ManuelZoltán, PásztiLopez-Luke, TzararaZong, ChengLópez-Ortega, AlbertoŻórawski, WojciechLópez-Periago, Ana M.Zornoza, EmilioLopez-Ramon, Maria VictoriaZota, Cezar B.Lorena, Garcia HeviaZotti, AldobenedettoLorenz, MichaelZou, ChangjunLorenzini, MarcoZou, GuoqiangLorenzo, JuliaZou, HaiyangLorusso, FeliceZou, HongyanLorusso, MassimoZou, QuanleLosada-Perez, PatriciaZou, YanLosada-Pérez, PatriciaZozulya, AlexeyLoschenov, Victor B.Zsuzsanna, MártonLosurdo, MariaZubar, TatianaLou, ZhichaoZubar, Tatiana I.Lou, ZimoZubavichus, Ya.V.Loureiro, Francisco J.A.Zubavichus, Yan V.Loureiro, Joana A.Zubrik, AntonLow, Sze ShinZuccari, GuendalinaLoya, J.A.Zukalová, MarkétaLozano, AdriánZukauskaite, AgneLozhkomoev, Aleksandr S.Zuliani, AlessioLozovoy, KirillZulkarnaen Bisri, SatriaLu, BaoyangZuo, JianLu, Guo-PingZuo, YingfengLu, HanfengZuorro, AntonioLu, HongŽupanič, AnžeLu, HongliangZuverza-Mena, NubiaLu, JianguoZverev, Vladimir I.Lu, JianmeiZvereva, Irina A.Lu, Kuo-ChangZych, DawidLu, LehuiZydney, AndrewLu, LiZypman, FredyLu, Ming-ChangZyryanov, Grigory V.Lu, Minghui



